# Synthesis and Structure-Affinity Relationships of Receptor Ligands with 1,3-Dioxane Structure

**DOI:** 10.3390/ph18091300

**Published:** 2025-08-29

**Authors:** Elisabeth Quick, Dirk Schepmann, Bernhard Wünsch

**Affiliations:** Institut für Pharmazeutische und Medizinische Chemie, Universität Münster, Corrensstraße 48, D-48149 Münster, Germany; quick@mailbox.org (E.Q.); dirk.schepmann@uni-muenster.de (D.S.)

**Keywords:** σ_1_ receptor, 2,4-disubstituted 1,3-dioxanes, butan-4-amines, synthesis, homologation, receptor affinity, selectivity, structure affinity relationships, opioid receptor affinity, NMDA receptor affinity, PCP binding site, ifenprodil binding site, lipophilicity, ligand-lipophilicity efficiency (LLE), metabolic stability

## Abstract

**Background/Objectives**: Ligands blocking σ_1_ receptors or NMDA receptors show promising pharmacological properties, such as analgesia or neuroprotection. It had been shown that depending on the stereochemistry and substitution pattern, 1,3-dioxnaes can selectively interact with either σ_1_ receptors or the phencyclidine binding site of NMDA receptors. Herein, systematic modifications of homologous aminobutyl substituted 1,3-dioxanes were conducted in order to identify ligands selectively addressing σ receptors or NMDA receptors. **Methods**: The first step of the synthesis*,* i.e., the acetalization of benzaldehyde (**7a**) or propiophenone (**7b**) with pentane-1,3,5-triol (**6**), determined the relative configuration of the envisaged 1,3-dioxanes bearing 4-aminobutyl substituents in 4-position. Multi-step homologation of ethanols **8** provided various primary, secondary and tertiary amines **14**, **16**–**19**, and **24**–**27**. The affinity towards σ_1_ and σ_2_ receptors as well as the PCP and ifenprodil binding sites of the NMDA receptor was systematically evaluated in radioligand receptor binding studies. **Results**: Only the primary amines **14b** and **24b** derived from propiophenone interacted moderately with the PCP binding site of the NMDA receptor. Within this class of compounds, the *N*-benzylamines **17** and **18** showed the highest σ_1_ affinity with high selectivity over the PCP binding site and at least preference over the σ_2_ receptor. The benzylamine **17a** (*K*_i_(σ_1_) = 31 nM, LLE = 6.19) and the pyrrolidine **19a** (*K*_i_(σ_1_) = 154 nM, LLE = 6.72) represent the most promising σ_1_ ligands of this compound series, when taking the lipophilicity and receptor selectivity into account. **Conclusions**: Both compounds showed medium metabolic stability in vitro rendering them promising candidates for further studies.

## 1. Introduction

Different activity profiles resulting from treatment of non-dependent and morphine-dependent chronic spinal dogs with different benzomorphans led to sub-differentiation of the opioid receptor into three subtypes, which were termed according to their prototypical ligands: µ-opioid receptor (morphine), κ-opioid receptor (ketocyclazocine) and σ-opioid receptor (SKF-10,047, *N*-allylnormetazocine) [1]. Later it was shown that the effects caused by racemic SKF-10,047 could not be antagonized by the opioid antagonist naloxone [2]. Therefore, the σ receptor was removed from the class of opioid receptors. Due to the similar activity profiles of SKF-10,047 and phencyclidine (PCP, 1-(1-phenylcyclohexyl)piperidine), the σ receptor was regarded to be identical with the PCP binding site of the *N*-methyl-d-aspartate (NMDA) receptor [3]. However, this hypothesis was also discarded, since the σ ligand haloperidol did not interact with the PCP binding site of the NMDA receptor. Moreover, the distribution of both binding sites in the central nervous system differs from each other [4]. Today, σ receptors are accepted as unique class of receptors containing σ_1_ and σ_2_ subtypes [4].

The confusion regarding the classification of σ_1_ receptors resulted from the use of racemic SKF-10,047 in the original experiments. Dextrorotatory benzomorphans, such as (+)-SKF-10,047 (*N*-allylnormetazocine) and (+)-pentazocine bind with high affinity and selectivity at σ_1_ receptors, whereas levorotatory benzomorphans, e.g., (−)-SKF-10,047 and (−)-pentazocine interact with opioid receptors and the PCP binding site of NMDA receptors. Small substituents at the N-atom (e.g.*,* H or CH_3_) lead to a preference for the PCP binding site over opioid receptors [5].

In 2016, the first X-ray crystal structures of the σ_1_ receptor in complex with atypical ligands 4-IBP and PD144418 were reported [6]^.^ In contrast to previous reports [7], these structures consisted of only one transmembrane helix, a short extracellular *N*-terminal part and a large cytosolic carboxy terminal domain containing the ligand binding site. The X-ray crystal structures of the σ_1_ receptor in complex with prototypical agonist (+)-pentazocine and prototypical antagonists NE-1001 and haloperidol appeared in 2018 [8]. These structures are characterized by an important ionic interaction between glutamate172 of the σ_1_ receptor and an ammonium moiety (protonated amino group) of the ligands. The human σ_2_ receptor has not yet been crystalized. However, it has been identified as the transmembrane protein 97 (TMEM97) located in the endoplasmic reticulum [9].

σ_1_ Receptors are located in various regions of the central nervous system. Among other neuropsychiatric and neurodegenerative diseases [10,11], σ_1_ receptors are involved in depression and neuropathic pain. σ_1_ Receptor knock-out mice showed an inconspicuous phenotype, but in the forced swimming test a depression-like behavior was observed [12,13]. Several antidepressants from different compound classes (e.g., fluoxetine, fluvoxamine, imipramine) interact strongly with the σ_1_ receptor [14]. In PET studies with the selective σ_1_ receptor ligand [^18^F]fluspidine, modified σ_1_ receptor expression in the central nervous system of patients suffering from major depression was observed [15]. Additionally, σ_1_ receptor antagonists represent promising candidates for the treatment of neuropathic pain, a special form of pain, which is difficult to treat with standard analgesic drugs. In the capsaicin mouse model of neuropathic pain [16], attenuation of pain-like effects was shown with σ_1_ receptor knock-out mice and with mice treated with σ_1_ receptor antagonists [17,18]. Recently, the analgesic drug, S1RA antagonizing σ_1_ receptors completed successfully phase II clinical trials for the treatment of neuropathic pain [17]. In general, σ_1_ ligands triggering pain-relieving effects in the capsaicin mouse model of neuropathic pain are regarded as σ_1_ antagonists [17].

In addition to the presence of σ_1_ receptors in the central nervous system, expression in different organs in the periphery (e.g., lung, liver, kidney, retina, and heart [19]) and several human tumors (e.g., breast, lung, prostate tumors) has been reported. A correlation between high σ_1_ receptor expression and strong metastasis of tumors (high aggressiveness) was observed resulting in an unfavorable prognosis [7,20]. Antagonists at σ_1_ receptors are able to reduce proliferation and survival of tumors [20].

**Figure 1:**
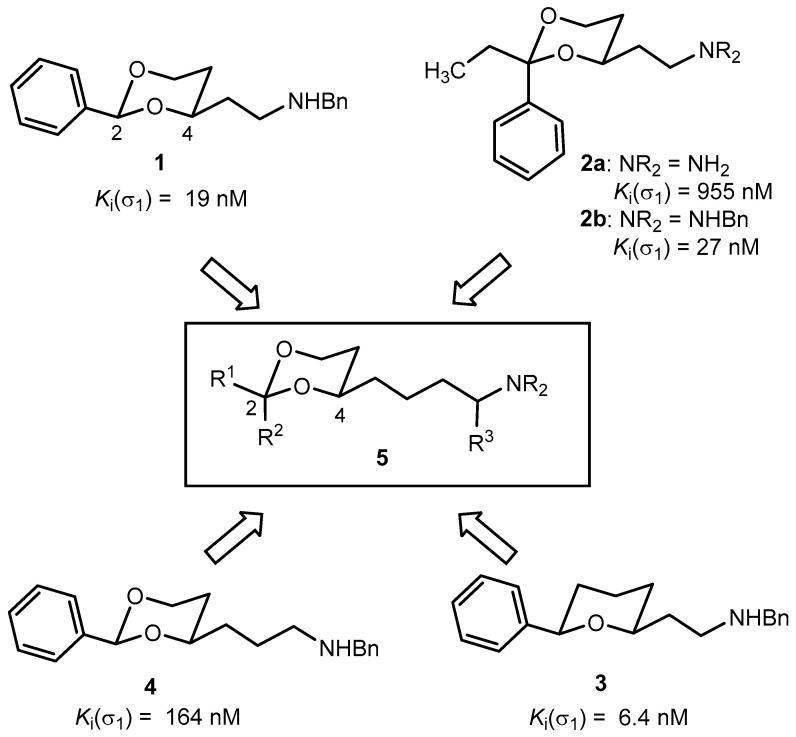
Design of potential σ_1_ ligands **5** with 1,3-dioxane scaffold and homologous butan-1-amine side chain.

In a previous study, we have investigated 1,3-dioxanes **1** bearing an aminoethyl moiety in 4-position. The racemic benzylamine **1** exhibited high σ_1_ affinity (*K*_i_ = 19 nM) and high selectivity over related σ_2_ receptors and the PCP binding site of the NMDA receptor. In the capsaicin assay of neuropathic pain, **1** showed 70% analgesic activity at the very low dose of 0.25 mg/kg body weight [21,22]. The (2*S*,4*R*)-configured enantiomer represents the eutomer (*K*_i_ = 6.0 nM) [22] (Figure 1).

The corresponding propiophenone derivative **2b** with a benzylamino moiety displayed comparable σ_1_ affinity (*K*_i_ = 27 nM), whereas the corresponding primary amine **2a** exhibited only low σ_1_ affinity (*K*_i_ = 955 nM). Instead of σ_1_ affinity, high affinity at the PCP binding site of the NMDA receptor was observed for **2a** (*K*_i_ = 19 nM) [22]. At the PCP binding site, the (*R*,*R*)-configured enantiomer represents the eutomer with a *K*_i_ value of 13 nM [22]. Replacement of the primary NH_2_ moiety of **2** by a benzylamino group brought back the high σ_1_ affinity (*K*_i_ = 27 nM [21] (Figure 1).

Replacement of one O-atom of the 1,3-dioxane ring by a CH_2_ moiety led to tetrahydropyran derivative **3**. Both enantiomers (2*S*,6*R*)-**3** and (2*R*,6*S*)-**3** revealed high σ_1_ affinity (*K*_i_ = 1.6 nM and 6.4 nM, respectively), were analgesically active in the mouse capsaicin assay and inhibited the growth of the human prostate cancer cell line DU145 [23] (Figure 1).

Homologation of ethan-1-amine **1** by one CH_2_ moiety provided propan-1-amine **4** with remarkable but reduced σ_1_ affinity (*K*_i_ = 164 nM). However, the selectivity over σ_2_ receptors and the PCP binding site of the NMDA receptor was retained. Even the corresponding primary amine (NH_2_ instead of NHBn) did not interact with the PCP binding site [21] (Figure 1).

This work was devoted to the next homologous butan-1-amines **5** bearing the 1,3-dioxane ring at 4-position. (Figure 1) At 2-position of the dioxane ring, the substitution pattern of the lead compounds **1**–**4** (benzaldehyde and propiophenone acetals) was preferred. In α-position of the amino moiety, an additional phenyl ring should be introduced. Finally, the amino group should be provided with various substituents. A series of differently substituted ligands should be prepared and pharmacologically evaluated in receptor binding studies.

## 2. Results and Discussion

### 2.1. Synthesis

**Scheme 1:**
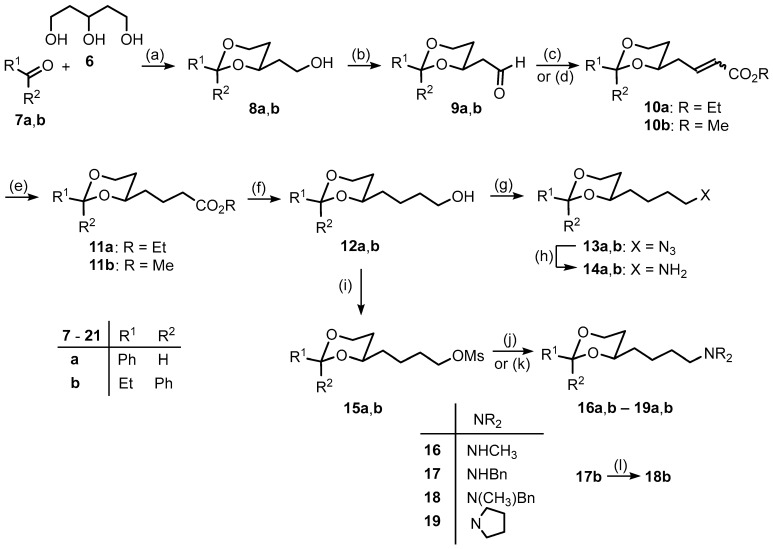
Synthesis of 4-substituted butan-1-amines **14** and **16**–**19**. Reagents and reaction conditions: (a) *p*TsOH, CH_2_Cl_2_ or toluene, reflux, 16 h or 1.5 h, 78% (**8a**), 90% (**8b**). (b) Oxalyl chloride, DMSO, CH_2_Cl_2_, −78 °C, 45 min, then NEt_3_, 91% (**9a**), 89% (**9b**). (c) Ph_3_P=CHCO_2_Et, THF, rt, 24 h, reflux, 2 h, 95% ((*E*)-**10a**: (*Z*)-**10a** = 10:1). (d) Ph_3_P=CHCO_2_CH_3_, THF, rt, 21 h, 95% ((*E*)-**10b**:(*Z*)-**10b** = 15:1). (e) NH_4_ HCO_2_, Pd/C, CH_3_OH, reflux, 2–3 h 92% (**11a**), 92% (**11b**). (f) LiBH_4_, THF, rt, 17 h, reflux, 2–3 h, 97% (**12a**), 96% (**12b**). (g) Zn(N_3_)_2_·2 pyridine, PPh_3_, DIAD (diisopropyl azodicarboxylate), toluene, rt, 5 h, 67% (**13a**), 58% (**13b**). (h) H_2_ (balloon), Pd/C, EtOAc, rt, 5 h, 84% (**14a**), 63% (**14b**). (i) CH_3_SO_2_Cl, CH_2_Cl_2_, NEt_3_, rt, 4 h, 96% (**15a**), 97% (**15b**). (j) CH_3_NH_2_, EtOH, reflux, 3–5 h, 70% (**16a**), 74% (**16b**). (k) HNR_2_, CH_3_CN, K_2_CO_3_, reflux, 14–25 h, 80–94%; (exception 32% for **17a**). (l) CH_2_=O, H_2_O, CH_3_OH, CH_2_Cl_2_, NaBH(OAc)_3_, rt, 16 h, 91% (**18b**).

The synthesis of butan-1-amines **5** started with acetalization of benzaldehyde (**7a**) or propiophenone (**7b**) with pentane-1,3,5-triol (**6**). Both reactions led to the thermodynamically favored diastereomer with an equatorially oriented 2-hydroxyethyl moiety in 4-position. With benzaldehyde, the *cis*-configured 1,3-dioxane **8a** was formed with the phenyl moiety adopting the energetically favored equatorial orientation. In case of propiophenone, an axial orientation of the ethyl moiety at 2-position would sterically interact with the 1,3-dioxane ring resulting in **8b** with equatorial orientation of the ethyl and axial orientation of the phenyl moiety [21,22]. All reaction products derived from **8a** and **8b** exhibit the same relative configuration as their corresponding parent primary alcohols **8a** and **8b** (Scheme 1).

*Swern* oxidation of the primary alcohols **8** led to aldehydes **9**, which reacted in a *Wittig* reaction with stabilized P-Ylides to afford the α,β-unsaturated esters **10a** and **10b**. As stabilized P-Ylides Ph_3_P=CHCO_2_R were employed, (*E*)-configured α,β-unsaturated esters (*E*)-**10** were produced predominantly (ratio (*E*):(*Z*) = 10:1 for **10a** and 15:1 for **10b**, respectively). For the hydrogenation of the α,β-unsaturated esters **10** ammonium formate and Pd/C were used to give the butanoates **11** in >90% yield. LiBH_4_ reduction in the esters **11** provided the primary alcohols **12**. The primary amines **14** were obtained by *Mitsunobu* reaction of the primary alcohols **12** with Zn(N_3_)_2_·2 pyridine, PPh_3_ and diisopropyl azodicarboxylate (DIAD) and subsequent hydrogenation of the resulting azides **13**. The secondary amines **16** and **17** and the tertiary amines **18** and **19** were prepared by nucleophilic substitution of the mesylates **15** with the corresponding primary and secondary amines. The tertiary amine **18b** was synthesized by reductive methylation of the secondary amine **17b** with formalin and NaBH(OAc)_3_ (Scheme 1).

**Scheme 2:**
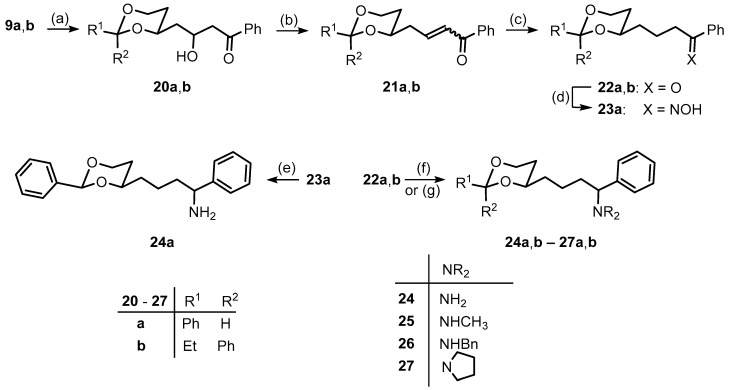
Synthesis of 4-substituted 1-phenylbutan-1-amines **24**–**27**. Reagents and reaction conditions: (a) Acetophenone, LDA, THF, −78 °C, 45–80 min, 69% (**20a**), 84% (**20b**). (b) CH_3_SO_2_Cl, NEt_3_, CH_2_Cl_2_, 0 °C, 2–2.5 h, 84% ((*E*)-**21a**), 93% ((*E*)- + (*Z*)-**21b**). (c) NH_4_ HCO_2_, Pd/C, CH_3_OH, reflux, 2 h, 82% (**22a**), 75% (**22b**). (d) NH_2_OH·HCl, NaOAc, EtOH, reflux, 7 h, 78% (**23a**). (e) LiAlH_4_, THF, rt, 19 h, then reflux, 4 h, 53%. (f) NH_4_OAc, CH_3_OH, NaBH_3_CN, reflux, 22–24 h, 56% (**24a**), 45% (**24b**). (g) HNR_2_, NaBH(OAc)_3_, CH_2_Cl_2_, reflux, 1–4 d, 33–83%.

For the synthesis of 1-phenyl substituted butan-1-amines **24**–**27**, an aldol addition of acetophenone to the aldehydes **9** was performed to afford the β-hydroxyketones **20**. Dehydration of β-hydroxyketones **20** with CH_3_SO_2_Cl and NEt_3_ led to the α,β-unsaturated ketones **21**. As for the α,β-unsaturated esters **10**, (*E*)-configured diastereomers were formed predominantly. Since hydrogenation of α,β-unsaturated ketones **21** with H_2_ and Pd/C led to various side products, the double bond of **21** was saturated by a transfer hydrogenation using ammonium formate (NH_4_HCO_2_) and Pd/C to obtain the ketones **22a** and **22b** in 82% and 75% yield, respectively. After conversion of ketone **22a** into its oxime **23a**, LiAlH_4_ reduction yielded the primary amine **24a**. Reductive amination of ketones **22a**,**b** with NH_4_OAc and NaBH_3_CN led to the primary amines **24a** and **24b**. For the reductive amination with primary and secondary amines, NaBH(OAc)_3_ was employed providing the methylamines **25**, benzylamines **26** and pyrrolidines **27** (Scheme 2).

### 2.2. Receptor Affinity

The affinity towards receptors was determined using tritium-labeled radioligands competing with the novel ligands for their binding sites. A low amount of bound radioactivity indicates high affinity of the test compounds to the respective receptor. The radioligands [^3^H](+)-pentazocine and [^3^H]di-*o*-tolylguanidine (in the presence of an excess of (+)-pentazocine) were used in the σ_1_ and σ_2_ assay, respectively. Membrane preparations from guinea pig brain (σ_1_ assay) and rat liver (σ_2_ assay) served as receptor material [24]. In addition to σ receptor affinity, the affinities towards the PCP [22,25] and ifenprodil binding sites [26] of the NMDA receptors were recorded. In Table 1, the receptor affinities of butan-1-amines **14**, **16**–**19** and **24**–**27** containing the 1,3-dioxane ring are summarized together with affinity data of some reference compounds.

The σ_1_ affinity of the linear primary amines **14a** and **14b** is rather low as they do not compete considerably with the radioligand [^3^H](+)-pentazocine at a concentration of 1 µM (IC_50_ > 1 µM). For the linear butan-1-amines **16**–**19,** a common trend was observed. Methylamines **16** showed lowest σ_1_ affinity, followed by the pyrrolidines **19** and the benzylamines **17**. An additional methyl moiety at the benzylamino moiety resulted in low nanomolar σ_1_ affinity of compounds **18a** (*K*_i_ = 6.3 nM) and **18b** (*K*_i_ = 8.7 nM). This observation is in good agreement with the pharmacophore models of σ_1_ receptor ligands postulating two lipophilic regions connected by a basic amino moiety [27]. The benzyl moiety at the N-atom of **17** and **18** represents the postulated second hydrophobic region. In this series of ligands, butanamines derived from propiophenone (**b**-series) display slightly higher σ_1_ affinity than their analogs derived from benzaldehyde (**a**-series). The tertiary *N*-benzyl-*N*-methylamines **18a** and **18b** are regarded as equipotent.

Butan-1-amines **24**–**27** already contains the second hydrophobic region in form of the additional phenyl moiety in 1-position of the side chain (compare σ_1_ pharmacophore models [27]). Due to the presence of this phenyl moiety, ligands **25** and **27** with small N-substituents show higher σ_1_ affinity than the corresponding ligands **16** and **19** with the same N-substituents. However, the introduction of an additional benzyl moiety at the amino group (**26a**, **26b**) appears to be unfavorable with respect to σ_1_ affinity. As observed for the linear butan-1-amines **15**–**19**, propiophenone derivatives **25b**–**27b** exhibit higher σ_1_ affinity than the analogous benzaldehyde derivatives **25a**–**27a**.

Homologation of the benzaldehyde-derived ethanamine **1** (*K*_i_ = 19 nM, Figure 1) and the corresponding propiophenone-derived ethanamine **2b** (*K*_i_ = 27 nM) [22] led to butanamines **17a** (*K*_i_ = 31 nM) and **17b** (*K*_i_ = 14 nM) with comparable σ_1_ affinity.

With exception of the propiophenone derivatives **19b** and **24b** all compounds reported herein exhibit selectivity or at least preference for the σ_1_ receptor over the σ_2_ subtype. The most potent σ_1_ ligands **18a** and **18b** show 5-fold and 3-fold σ_1_:σ_2_ receptor selectivity. The highest σ_1_:σ_2_ selectivity was observed for the benzylamine **17a** (approx. 7-fold).

For a short period of time, the σ_1_ receptor was regarded to be identical with the PCP binding site at the NMDA receptor. This hypothesis resulted from similar ligand profiles of both receptors. Therefore, the affinity of the ligands towards the PCP binding site was recorded using [^3^H](+)-MK-801 as competitive radioligand and pig brain preparations as receptor material [22,25]. A considerable affinity towards the PCP binding site of the NMDA receptor was detected only for the primary amines **14b** (*K*_i_ = 731 nM) and **24b** (*K*_i_ = 524 nM) derived from propiophenone. This observation correlates well with the high PCP affinity of the 1,3-dioxolane derivative etoxadrol (**28**, *K*_i_ = 22 nM) [22] (Figure S1) and the 1,3-dioxane **2a** (*K*_i_ = 13 nM (*R*,*R*)-enantiomer) [22]. (Figure 1) The moderate PCP affinity of the benzylamine **17b** (*K*_i_ = 569 nM) appears to be unexpected.

Due to similar pharmacophore models of σ ligands and ligands for the ifenprodil binding site of the NMDA receptor, the affinity towards the ifenprodil binding site was recorded in competitive receptor binding studies with tritium-labeled ifenprodil and membrane preparations from L(tk-) cells overexpressing NMDA receptors with GluN2B subunit [26]. The most potent σ_1_ ligands **17a**,**b** and **18a**,**b** showed affinity towards this binding site as well, but at least a preference for σ_1_ receptors. Whereas the benzaldehyde-derived 1,3-dioxanes **17a** and **18a** demonstrated high >30- and >40-fold selectivity for the σ_1_ receptor against the ifenprodil binding site, the propiophenone derivatives **17b** and **18b** showed comparable affinities towards both receptors.

As detailed in the introduction, σ receptors were originally classified as opioid receptor subtype. Therefore, the opioid receptor affinity of the high-affinity σ_1_ ligands **17** and **18** was also recorded in receptor binding studies [28]. (Table S1, Supporting Information) The benzaldehyde-derived benzylamine **17a** (*K*_i_(σ_1_) = 31 nM) showed high selectivity for the σ_1_ receptor over µ- and κ-opioid receptors, but only 9-fold selectivity over δ-opioid receptors. The more potent propiophenone-derived benzylamine **17b** and both tertiary amines **18a** and **18b** exhibited high selectivity over all three opioid receptor subtypes.

### 2.3. Lipophilicity and Metabolic Stability (In Vitro) of Selected σ_1_ Receptor Ligands

A balanced lipophilicity is crucial for the pharmacokinetics and pharmacodynamics of drugs. As measure for the lipophilicity, the logD_7.4_ value of selected compounds was recorded using the recently developed micro-shake flask method. In this method, a compound was distributed between a MOPS buffer pH 7.4 and *n*-octanol and the amount of compound in the buffer layer was determined by MS analysis [29] (Table 2).

The logD_7.4_ values of propiophenone derivatives (**b**-series) is approx. 1.2 log units higher than the logD_7.4_ value of benzaldehyde derivatives (**a**-series). The introduction of a methyl moiety increased the logD_7.4_ value by approx. 1 log unit (compare **17** and **18**). Replacement of the benzylamino moiety by a pyrrolidino moiety reduced the logD_7.4_ value by approx. 1.2 log units (compare **17** and **19**). The additional phenyl moiety in the side chain of **27** increased the logD_7.4_ value by approx. 2 log units (compare **19** and **27**).

The recorded logD_7.4_ values were used to calculate the ligand-lipophilicity efficiency (LLE) [30] of the σ_1_ ligands. The LLE value combines the affinity or activity of a ligand with its lipophilicity to avoid the development of very lipophilic biologically active compounds. It is assumed that compounds with high LLE values have favorable pharmacokinetics and high druglikeness [30]. Due to their high polarity, the benzylamine **17a** and the pyrrolidine **19a** show the highest LLE values despite their only moderate σ_1_ receptor affinity of 31 nM (**17a**) and 154 nM (**19a**). Although the tertiary amines **18a** and **18b** exhibit low nanomolar σ_1_ affinity, their high lipophilicity reduced their LLE values to 5.71 and 4.52.

In order to get an impression on the metabolic stability, selected butanamines were incubated with mouse liver microsomes and NADPH. After an incubation period of 90 min, the amount of unchanged parent compound was determined by LC-MS [29]. The metabolic stability of the investigated butanamines **17**–**19** and **27** is very similar, approx. 50–60% of the parent compounds were found unchanged after 90 min. As exceptions, the pyrrolidine **27a** was metabolically more stable (76% intact after 90 min) and the tertiary amine **18b** showed faster biotransformation (36% intact after 90 min).

## 3. Materials and Materials

### 3.1. Synthetic Procedures

**2-(*cis*-2-Phenyl-1,3-dioxan-4-yl)ethanol (8a).**

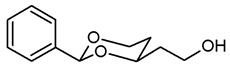


Pentane-1,3,5-triol (**6**, 1.69 g, 14 mmol), benzaldehyde (**7a**, 3.5 mL, 35 mmol) and *p*-toluenesulfonic acid (75 mg) were dissolved in CH_2_Cl_2_ abs. (60 mL) and heated to reflux for 16 h, using an inverse Dean-Stark apparatus. For workup, the reaction mixture was washed with saturated aqueous solution of NaHCO_3_ (3 × 20 mL), dried (K_2_CO_3_) and the solvent was removed in vacuo. The residue was purified by flash column chromatography (Ø 6 cm, cyclohexane:thyl acetate = 1:1, length 20 cm, fraction 65 mL, R_f_ = 0.28). Colorless oil, yield 2.27 g (78%). C_12_H_16_O_3_, M_r_ = 208.3. MS (EI): *m*/*z* [%] = 208 (M, 33), 207 (M-H, 100), 177 (M-CH_2_OH, 13), 105 (PhCO, 47), 77 (Ph, 42). IR (neat): $\tilde{\nu }$ [cm^−1^] = 3420 (O-H), 3066, 3034 (Ar-H), 2947, 2857 (C-H), 1098 (C-O-C), 749, 697 (arom. monosubst.). ^1^H NMR (CDCl_3_): δ [ppm] = 1.54 (dtd, J = 13.1/2.5/1.4 Hz, 1H, 5-H_eq_), 1.79–1.99 (m, 3H, C*H*_2_-CH_2_-OH and 5-H_ax_), 2.11 (dd, J = 6.1/4.7 Hz, 1H, CH_2_-O*H*), 3.79–3.90 (m, 2H, C*H*_2_-OH), 3.99 (td, J = 11.7/2.6 Hz, 1H, 6-H_ax_), 4.09–4.16 (m, 1H, 4-H_ax_), 4.28 (ddd, J = 11.4/5.0/1.1 Hz, 1H, 6-H_eq_), 5.54 (s, 1H, 2-H_ax_), 7.30–7.40 (m, 3H, Ar-H), 7.46–7.48 (m, 2H, Ar-H).HPLC (method MeOH): purity 96.8%, t_R_ = 12.27 min.

**2-(*trans*-2-Ethyl-2-phenyl-1,3-dioxan-4-yl)ethanol (8b).**

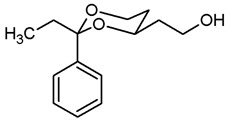


Pentane-1,3,5-triol (**6**, 2.01 g, 16.6 mmol), propiophenone (**7b** 4.4 mL, 33.2 mmol) and *p*-toluenesulfonic acid (75 mg) were dissolved in toluene (90 mL) and heated to reflux for 1.5 h, using a Dean-Stark apparatus filled with molecular sieves 4 Å. For workup, the reaction mixture was washed with saturated aqueous solution of NaHCO_3_ (3 × 30 mL) and with brine (1 × 30 mL) and dried (K_2_CO_3_). The solvent was removed in vacuo, and the residue was purified by flash column chromatography (Ø 8 cm, cyclohexane:ethyl acetate = 7:3, length 17 cm, fraction 65 mL, R_f_ = 0.24). Colorless oil, yield 3.51 g (90%). C_14_H_20_O_3_, M_r_ = 236.3. MS (EI): *m*/*z* [%] = 237 (M + H, 24), 207 (M-CH_3_-CH_2,_ 100), 105 (Ph-CO, 67). IR (neat): $\tilde{\nu }$ [cm^−1^] = 3420 (O-H), 2926, 2875 (C-H), 756, 703 (arom. monosubst.). ^1^H NMR (CDCl_3_): δ [ppm] = 0.80 (t, J = 7.5 Hz, 3 H, diox-CH_2_-C*H*_3_), 1.25–1.30 (m, 1 H, 5-H_eq_), 1.69–1.89 (m, 3 H, 5-H_ax_, C*H*_2_-CH_2_-OH), 1.75 (q, J = 7.5 Hz, 2 H, diox-C*H*_2_-CH_3_), 2.58 (s, 1 H, O-*H*), 3.81 (td, J = 12.0/2.5 Hz, 1 H, 6-H_ax_), 3.87–3.91 (m, 3 H, 6-H_eq_, C*H*_2_-OH), 3.93–4.00 (m, 1 H, 4-H_ax_), 7.29–7.42 (m, 5 H, Ar-H). HPLC (method ACN): purity 99.9%, tR = 17.54 min.

**2-(*cis*-2-Phenyl-1,3-dioxan-4-yl)acetaldehyde (9a).**

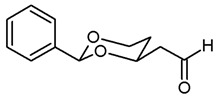


Under N_2_ atmosphere, oxalyl chloride (0.51 mL, 6 mmol) was dissolved in CH_2_Cl_2_ abs. (25 mL) and cooled down to −78 °C. At this temperature, a solution of DMSO (0.85 mL, 12 mmol) in CH_2_Cl_2_ abs. (7.5 mL) was added very slowly (using a syringe pump, 7 mL/h) and the mixture was stirred for 15 min at −78 °C. Then, a solution of the alcohol **8a** (1.043 g, 5 mmol) in CH_2_Cl_2_ abs. (7.5 mL) was added (using a syringe pump, 12 mL/h). The mixture was stirred for additional 45 min at −78 °C before NEt_3_ (3.5 mL, 25 mmol) was added. After warming to rt, *n*-hexane (40 mL) was added. The precipitate was filtered off using a glass suction filter and washed several times with Et_2_O. The filtrate was concentrated (600 mbar, 40 °C) and the filtration procedure was repeated once. The solvent was removed in vacuo, and the residue was purified by flash column chromatography (Ø 5.5 cm, cyclohexane:ethyl acetate = 8:2, length 20 cm, fraction 65 mL, R_f_ = 0.16). Colorless oil with sweet smell, yield 0.994 g (91%). C_12_H_14_O_3_, M_r_ = 206.3. MS (EI): *m*/*z* = 207 (M + H, 53), 205 (M-H, 76), 177 (M-CHO, 6), 105 (PhCO, 100), 77 (Ph, 81). IR (neat): $\tilde{\nu }$ [cm^−1^] = 2961, 2921, 2856 (C-H), 2736 (C-H_aldehyde_), 1722 (C=O), 1099 (C-O), 752, 698 (arom. monosubst.). **^1^**H NMR (CDCl_3_): δ [ppm] = 1.64 (dtd, J = 13.2/2.5/1.4 Hz, 1H, 5-H_eq_), 1.90 (dddd, J = 13.1/12.3/11.4/5.0 Hz, 1H, 5-H_ax_), 2.63 (ddd, J = 17.0/5.2/1.6 Hz, 1H, C*H*_2_-CHO), 2.83 (ddd, J = 17.0/7.3/2.1 Hz, 1H, C*H*_2_-CHO), 4.02 (td, J = 12.2/2.6 Hz, 1H, 6-H_ax_), 4.29 (ddd, J = 11.5/5.0/1.4 Hz, 1H, 6-H_eq_), 4.41–4.47 (m, 1 H, 4-H_ax_), 5.57 (s, 1H, 2-H_ax_), 7.33–7.38 (m, 3H, Ar-H), 7.45–7.48 (m, 2 H, Ar-H), 9.85 (t, J = 1.8 Hz, 1H, C*H*O). HPLC (method ACN): purity 90.4%, t_R_ = 14.05 min. The substance was rather unstable and always used within three days after purification.

***trans*-(2-Ethyl-2-phenyl-1,3-dioxan-4-yl)acetaldehyde (9b).**

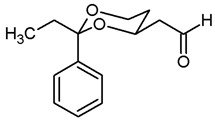


Under N_2_ atmosphere, oxalyl chloride (0.87 mL, 10.15 mmol) was dissolved in CH_2_Cl_2_ abs. (26 mL) and cooled down to −78 °C. At this temperature, a solution of DMSO (1.44 mL, 20.30 mmol) in CH_2_Cl_2_ abs. (12 mL) was added very slowly (using a syringe pump, 9 mL/h) and the mixture was stirred for 15 min at −78 °C. Then a solution of the alcohol **8b** (2.00 g, 8.46 mmol) in CH_2_Cl_2_ abs. (12 mL) was added (using a syringe pump, 18 mL/h). The mixture was stirred for additional 45 min at −78 °C before NEt_3_ (6.6 mL, 42.30 mmol) was added. After warming to rt, *n*-hexane (40 mL) was added. The precipitate was filtered off using a glass suction filter and washed several times with Et_2_O. The filtrate was concentrated (600 mbar, 40 °C) and the filtration procedure was repeated once. The solvent was removed in vacuo, and the residue was purified by flash column chromatography (6 cm, cyclohexane:ethyl acetate = 9.25:0.75, length 18 cm, fraction 65 mL, R_f_ = 0.09). Colorless solid, mp 55.8 °C, yield 1.76 g (89%). C_14_H_18_O_3_, M_r_ = 234.3. MS (EI): *m*/*z* [%] = 235 (M + H, 41), 205 (M-CH_3_-CH_2_, 100), 105 (Ph-CO, 52). IR (neat): $\tilde{\nu }$ [cm^−1^] = 2972, 2930, 2877 (C-H), 2730 (C-H_aldehyde_), 1725 (C=O), 758, 704 (arom. monosubst.). ^1^H NMR (CDCl_3_): δ [ppm] = 0.79 (t, J = 7.5 Hz, 3 H, diox-CH_2_-C*H*_3_), 1.34–1.39 (m, 1 H, 5-H_eq_), 1.73 (q, J = 7.5 Hz, 2 H, diox-C*H*_2_-CH_3_), 1.79 (qd, J = 12.3/5.4 Hz, 1 H, 5-H_ax_), 2.50 (ddd, J = 16.5/4.4/1.6 Hz, 1 H, C*H*_2_-CHO), 2.73 (ddd, J = 16.5/8.0/2.5 Hz, 1 H, C*H*_2_-CHO), 3.83 (td, J = 11.9/2.5 Hz, 1 H, 6-H_ax_), 3.90 (ddd, J = 11.5/5.4/1.5 Hz, 1 H, 6-H_eq_), 4.22–4.29 (m, 1 H, 4-H_ax_), 7.29–7.33 (m, 1 H, Ar-H), 7.36–7.43 (m, 4 H, Ar-H), 9.91 (dd, J = 2.3/1.7 Hz, 1 H, C*H*O). HPLC (method ACN): purity 98.7%, tR = 18.15 min.

**Ethyl (2*Z*)-4-(*cis*-2-phenyl-1,3-dioxan-4-yl)but-2-enoate ((*Z*)-10a) and ethyl (2*E*)-4-(*cis*-2-phenyl-1,3-dioxan-4-yl)but-2-enoate ((*E*)-10a).**

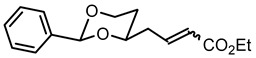


Under N_2_ atmosphere the commercially available P-ylide Ph_3_P=CH-CO_2_Et (1.23 g, 4.5 mmol) was dissolved in THF abs. (20 mL). A solution of the aldehyde **9a** (619 mg, 3 mmol) in THF abs. (8 mL) was added, and the solution was diluted with THF to a total volume of 40 mL. The mixture was stirred for 24 h at rt and then heated to reflux for 2 h. Water (30 mL) and brine (15 mL) were added, and the mixture was extracted with ethyl acetate (3 × 30 mL). The combined organic layers were washed with brine (1 × 40 mL) and dried (K_2_CO_3_). The solvent was removed in vacuo, and the residue was adsorbed on silica gel and loaded on the column (Ø 5.5 cm, cyclohexane:ethyl acetate = 9:1, length 18 cm, fraction 65 mL).

(*Z*)-**10a** (R_f_ = 0.15): Colorless oil, yield 76 mg (9.1%). C_16_H_20_O_4_, M_r_ = 276.3. IR (neat): $\tilde{\nu }$ [cm^−1^] = 2977, 2922, 2851 (C-H), 1714 (CO_2_R), 1646 (C=C), 1098 (C-O), 750, 697 (arom. monosubst). **^1^**H NMR (CDCl_3_): δ [ppm] = 1.29 (t, J = 7.1 Hz, 3 H, CO_2_CH_2_C*H*_3_), 1.56 (dtd, J = 13.3/3.8/1.4 Hz, 1 H, 5-H_eq_), 1.89 (dddd, J = 13.1/12.3/11.4/5.1 Hz, 1 H, 5-H_ax_), 2.87–2.95 (m, 1 H, diox-C*H*_2_-CH=CH), 3.10 (dddd, J = 15.7/7.6/4.5/1.7 Hz, 1 H, diox-C*H*_2_-CH=CH), 3.97 (ddd, J = 12.3/11.6/2.6 Hz, 1 H, 6-H_ax_), 3.96–4.02 (m, 1 H, 4-H_ax_), 4.18 (q, J = 7.1 Hz, 2 H, CO_2_C*H*_2_CH_3_), 4.27 (ddd, J = 11.3/4.9/1.1 Hz, 1 H, 6-H_eq_), 5.52 (s, 1 H, 2-H_ax_), 5.88 (dt, J = 11.6/1.8 Hz, 1 H, CH=C*H*-CO_2_R), 6.45 (ddd, J = 11.6/7.7/6.9 Hz, 1 H, C*H*=CH-CO_2_R), 7.31–7.39 (m, 3 H, Ar-H), 7.48–7.50 (m, 2 H, Ar-H). HPLC (method ACN): purity 93.0%, t_R_ = 20.90 min. Contamination with 2.9% of (*E*)-**10a** (t_R_ = 20.35 min) was observed.

(*E*)-**10a** (R_f_ = 0.12: Colorless oil, yield 712 mg (86%). C_16_H_20_O_4_, M_r_ = 276.3. MS (ESI): *m*/*z* [%] = 277 (M + H, 10), 294 (M + NH_4_, 22), 299 (M + Na, 26), 575 (2 × M + Na, 20), 577 (M + Ph_3_P = O + Na, 100). IR (neat): $\tilde{\nu }$ [cm^−1^] = 2979, 2853 (C-H), 1715 (CO_2_R), 1656 (C=C), 1099 (C-O), 751, 697 (arom. monosubst). 1H NMR (CDCl_3_): δ [ppm] = 1.29 (t, J = 7.1 Hz, 3 H, CO_2_CH_2_-C*H*_3_), 1.53–1.58 (m, 1 H, 5-H_eq_), 1.85 (dddd, J = 13.1/12.3/11.4/5.0 Hz, 1 H, 5-H_ax_), 2.46 (dddd, J = 14.7/7.4/5.7/1.4 Hz, 1 H, diox-C*H*_2_-CH=CH), 1.60 (dtd, J = 14.8/6.9/1.5 Hz, 1 H, diox-C*H*_2_-CH=CH), 3.96 (td, J = 12.0/2.6 Hz, 1 H, 6-H_ax_), 3.95–4.02 (m, 1 H, 4-H_ax_), 4.19 (q, J = 7.1 Hz, 2 H, CO_2_C*H*_2_-CH_3_), 4.28 (ddd, J = 11.4/5.0/1.2 Hz, 1 H, 6-H_eq_), 5.52 (s, 1 H, 2-H_ax_), 5.93 (dt, J = 15.7/1.4 Hz, 1 H, CH=C*H*-CO_2_R), 7.01 (dt, J = 15.6/7.3 Hz, 1 H, C*H*=CH-CO_2_R), 7.33–7.39 (m, 3 H, Ar-H), 7.48–7.50 (m, 2 H, Ar-H). HPLC (method ACN): purity 93.4%, t_R_ = 20.34 min. Contamination with 1.7% of (*Z*)-**10a** (t_R_ = 20.35 min) was observed.

**Methyl (2*Z*)-4-(*trans*-2-ethyl-2-phenyl-1,3-dioxan-4-yl)but-2-enoate ((*Z*)-10b) and methyl (2*E*)-4-(*trans*-2-ethyl-2-phenyl-1,3-dioxan-4-yl)but-2-enoate ((*E*)-10b).**

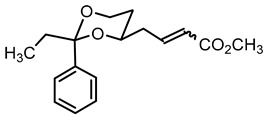


Under N_2_ atmosphere the commercially available P-ylide Ph_3_P=CH-CO_2_CH_3_ (2.10 g, 6.27 mmol) was dissolved in THF abs. (20 mL). A solution of the aldehyde **9b** (983 mg, 4.18 mmol) in THF abs. (10 mL) was added, and the solution was diluted with THF to a total volume of 65 mL. The mixture was stirred for 21 h at rt. Additional P-ylide (699 mg, 2.09 mmol) and THF abs. (10 mL) were added, and the mixture was stirred for another 2 h. Then, water (30 mL) and brine (15 mL) were added, and the mixture was extracted with ethyl acetate (3 × 30 mL). The combined organic layers were dried (K_2_CO_3_). The solvent was removed in vacuo, and the residue was adsorbed on silica gel and given on the column (Ø 5.5 cm, cyclohexane:ethyl acetate = 9.5:0.5, length 17 cm, fraction 65 mL).

(*Z*)**-10b** (R_f_ = 0.22): Colorless oil, yield 69 mg (5.7%). C_17_H_22_O_4_, M_r_ = 290.4. MS (ESI): *m*/*z* [%] = 313 (M + Na, 100). IR (neat): $\tilde{\nu }$ [cm^−1^] = 2946, 2873 (C-H), 1721 (CO_2_R), 1647 (C=C), 758, 705 (arom. monosubst.). ^1^H NMR (CDCl_3_): δ [ppm] = 0.81 (t, J = 7.5 Hz, 3 H, diox-CH_2_-C*H*_3_), 1.29–1.33 (m, 1 H, 5-H_eq_), 1.74 (q, J = 7.5 Hz, 2 H, diox-C*H*_2_-CH_3_), 1.71–1.82 (m, 1 H, 5-H_ax_), 2.84 (dtd, J = 15.4/7.3/1.8 Hz, 1 H, diox-C*H*_2_-CH=CH), 2.96 (dddd, J = 15.6/7.4/4.5/1.8 Hz, 1 H, diox-C*H*_2_-CH=CH), 3.71 (s, 3 H, CO_2_C*H*_3_), 3.74–3.81 (m, 1 H, 6-H_ax_), 3.75–3.84 (m, 1 H, 4-H_ax_), 3.88 (ddd, J = 11.4/5.1/1.3 Hz, 1 H, 6-H_eq_), 5.93 (dt, J = 11.6/1.7 Hz, 1 H, CH=C*H*-CO_2_CH_3_), 6.51 (dt, J = 11.6/7.3 Hz, 1 H, C*H*=CH-CO_2_CH_3_), 7.26–7.40 (m, 5 H, Ar-H). HPLC (method ACN): purity 99.0%, t_R_ = 22.07 min.

(*E*)-**10b** (R_f_ = 0.15): Colorless solid, mp 66.2 °C, yield 1.09 g (90%). C_17_H_22_O_4_, M_r_ = 290.4. MS (ESI): *m*/*z* [%] = 291 (M + H, 25), 313 (M + Na, 100). IR (neat): $\tilde{\nu }$ [cm^−1^] = 2980, 2943 (C-H), 1719 (CO_2_R), 1662 (C=C), 759, 702 (arom. monosubst.). ^1^H NMR (CDCl_3_): δ [ppm] = 0.81 (t, J = 7.5 Hz, 3 H, diox-CH_2_-C*H*_3_), 1.28–1.32 (m, 1 H, 5-H_eq_), 1.74 (q, J = 7.5 Hz, 2 H, diox-C*H*_2_-CH_3_), 1.67–1.77 (m, 1 H, 5-H_ax_), 2.33–2.40 (m, 1 H, C*H*_2_-CH=CH), 2.49 (dtd, J = 14.3/7.0/1.2 Hz, 1 H, C*H*_2_-CH=CH), 3.75 (s, 3 H, CO_2_C*H*_3_), 3.74–3.83 (m, 2 H, 6-H_ax_ + 4-H_ax_), 3.88 (ddd, J = 11.4/5.0/1.2 Hz, 1 H, 6-H_eq_), 5.92 (d broad, J = 15.7 Hz, 1 H, CH=C*H*-CO_2_CH_3_), 7.03 (dt, J = 15.7/7.4 Hz, 1 H, C*H*=CH-CO_2_CH_3_), 7.25–7.40 (m, 5 H, Ar-H). HPLC (method ACN): purity 99.0%, t_R_ = 21.68 min.

**Ethyl 4-(*cis*-2-phenyl-1,3-dioxan-4-yl)butanoate (11a).**

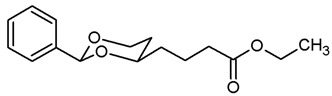


The α,β-unsaturated ester (*E*/*Z*)-**10a** (900 mg, 3.25 mmol) was dissolved in methanol abs. (75 mL). Pd/C (10% Pd, 90 mg, 10% *m*/*m*) and ammonium formate (1.01 g, 16.25 mmol) were added. After purging with N_2_, the mixture was heated to reflux for 2 h, then cooled down to rt and filtered through Celite^®^. The solvent was removed in vacuo. In order to remove excess ammonium formate, the residue was dissolved in water (80 mL) and extracted with ethyl acetate (3 × 30 mL). The combined organic layers were washed with brine and dried (K_2_CO_3_). The solvent was removed in vacuo, and the remaining oil was purified by flash column chromatography (Ø 4 cm, *n*-hexane:ethyl acetate = 9:1, length 18 cm, fraction 30 mL, R_f_ = 0.12). Colorless oil, yield 833 mg (92%). C_16_H_22_O_4_, M_r_ = 278.3. MS (EI): *m*/*z* [%] = 278 (M, 19), 155 (M-PhCHO-H_2_O + H, 100), 91 (Ph-CH_2_, 70). IR (neat): $\tilde{\nu }$ [cm^−1^] = 2952, 2849 (C-H), 1732 (C=O), 750, 698 (arom. monosubst.). ^1^H NMR (CDCl_3_): δ [ppm] = 1.25 (t, J = 7.1 Hz, 3 H, CO_2_CH_2_C*H*_3_), 1.51–1.91 (m, 6 H, 5-H_ax+eq_, diox-(C*H*_2_)_2_), 2.35 (t, J = 7.4 Hz, 2 H, C*H*_2_CO_2_Et), 3.82–3.88 (m, 1 H, 4-H_ax_), 3.96 (td, J = 11.9/2.5 Hz, 1 H, 6-H_ax_) 4.13 (q, J = 7.1 Hz, 2 H, CO_2_C*H*_2_CH_3_), 4.27 (dd broad, J = 11.4/4.9 Hz, 1 H, 6-H_eq_), 5.50 (s, 1 H, 2-H_ax_), 7.30–7.38 (m, 3 H, Ar-H), 7.48–7.50 (m, 2 H, Ar-H). HPLC (method ACN): purity 97.1%, t_R_ = 20.34 min.

**Methyl 4-(*trans*-2-ethyl-2-phenyl-1,3-dioxan-4-yl)butanoate (11b).**

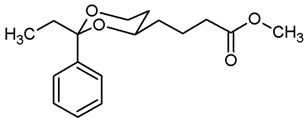


The α,β-unsaturated ester (*E*/*Z*)-**11b** (600 mg, 2.17 mmol) was dissolved in methanol abs. (50 mL). Pd/C (10% Pd, 60 mg, 10% *m*/*m*) and ammonium formate (808 mg, 13.02 mmol) were added. After purging with N_2_, the mixture was heated to reflux for 3 h (until the IR-band at 1648 cm^−1^ was gone), then cooled down to rt and filtered through Celite^®^. The solvent was removed in vacuo. In order to remove excess ammonium formate, the residue was dissolved in water (60 mL) and extracted with ethyl acetate (3 × 30 mL). The combined organic layers were washed with brine (1 × 30 mL) and dried (K_2_CO_3_). The solvent was removed in vacuo, and the residue was purified by flash column chromatography (Ø 4 cm, cyclohexane:ethyl acetate = 9:1, length 17 cm, fraction 30 mL, R_f_ = 0.20). Colorless oil, yield 580 mg (92%). C_17_H_24_O_4_, M_r_ = 292.4. MS (EI): *m*/*z* [%] = 292 (M, 18), 263 (M-CH_2_CH_3_, 18), 81 (100). IR (neat): $\tilde{\nu }$ [cm^−1^] = 2945, 2871 (C-H), 1738 (C=O), 758, 705 (arom. monosubst.). ^1^H NMR (CDCl_3_): δ [ppm] = 0.79 (t, J = 7.5 Hz, 3 H, diox-CH_2_-C*H*_3_), 1.24–1.29 (m, 1 H, 5-H_eq_), 1.41–1.50 (m, 1 H, diox-(C*H*_2_)_2_), 1.56–1.78 (m, 3 H, 5-H_ax_, diox-(C*H*_2_)_2_), 1.73 (q, J = 7.5 Hz, 2 H, diox-C*H*_2_-CH_3_), 1.85–1.96 (m, 1 H, diox-(C*H*_2_)_2_), 2.31–2.43 (m, 2 H, C*H*_2_CO_2_CH_3_), 3.61–3.70 (m, 1 H, 4-H_ax_), 3.68 (s, 3 H, CO_2_C*H*_3_), 3.77 (td, J = 11.6/2.6 Hz, 1 H, 6-H_ax_), 3.87 (ddd, J = 11.4/5.0/1.3 Hz, 1 H, 6-H_eq_), 7.27–7.40 (m, 5 H, Ar-H). HPLC (method ACN): purity > 99.9%, t_R_ = 21.67 min.

**4-(*cis*-2-Phenyl-1,3-dioxan-4-yl)butan-1-ol (12a).**

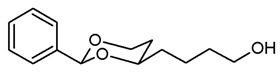


Under N_2_ atmosphere, a solution of the saturated ester **11a** (650 mg, 2.33 mmol) in THF abs. (40 mL) was cooled down to 0 °C. LiBH_4_ (4 M solution in THF, 2.3 mL, 9.2 mmol) was added slowly. The mixture was stirred at rt for 17 h and then heated to reflux for 2 h. For workup, the mixture was cooled down to rt and 0.1 M HCl (60 mL) was added dropwise. When H_2_ formation was finished, the mixture was saturated with NaCl and extracted with ethyl acetate (2 × 30 mL). The combined organic layers were washed with saturated aqueous solution of NaHCO_3_ (30 mL) and with brine (30 mL). After drying (K_2_CO_3_), the solvent was removed in vacuo, and the residue was purified by flash column chromatography (Ø 4 cm, cyclohexane:ethyl acetate = 7:3, length 17 cm, fraction 30 mL, R_f_ = 0.12). Colorless oil, yield 532 mg (97%). C_14_H_20_O_3_, M_r_ = 236.3. MS (EI): *m*/*z* [%] = 235 (M-H, 17), 219 (M-H_2_O + H, 27), 105 (PhCO, 73), 91 (PhCH_2_, 57). IR (neat): $\tilde{\nu }$ [cm^−1^] = 3410 (O-H), 2938, 2860 (C-H), 1103 (C-O), 750, 697 (arom. monosubst.) ^1^H NMR (CDCl_3_): δ [ppm] = 1.42–1.74 (m, 7 H, 5-H_eq_, diox-(C*H*_2_)_3_), 1.82 (tdd, J = 12.5/11.3/5.0 Hz, 1 H, 5-H_ax_), 3.66 (t, J = 6.3 Hz, 2 H, C*H*_2_-OH), 3.81–3.87 (m, 1 H, 4-H_ax_), 3.96 (td, J = 11.9/2.6 Hz, 1 H, 6-H_ax_), 4.27 (ddd, J = 11.4/5.0/1.2 Hz, 1 H, 6-H_eq_), 5.50 (s, 1 H, 2-H_ax_), 7.30–7.38 (m, 3 H, Ar-H), 7.48–7.50 (m, 2 H, Ar-H). A signal for the O*H* proton is not seen in the spectrum. HPLC (method ACN): purity 95.2%, t_R_ = 16.90 min.

**4-(*trans*-2-Ethyl-2-phenyl-1,3-dioxan-4-yl)butan-1-ol (12b).**

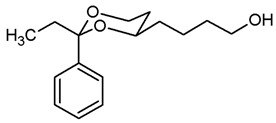


Under N_2_ atmosphere, a solution of the saturated ester **11b** (500 mg, 1.71 mmol) in THF abs. (25 mL) was cooled down to 0 °C. LiBH_4_ (4 M solution in THF, 6.84 mL, 6.84 mmol) was added slowly. The mixture was stirred at rt for 17 h and then heated to reflux for 3 h. For workup, the mixture was cooled down to rt and 0.1 M HCl (60 mL) was added dropwise. When H_2_ formation was finished, the mixture was extracted with ethyl acetate (2 × 30 mL). The combined organic layers were washed with saturated aqueous solution of NaHCO_3_ (30 mL) and with brine (30 mL). After drying (K_2_CO_3_), the solvent was removed in vacuo, and the residue was purified by flash column chromatography (Ø 4 cm, cyclohexane:ethyl acetate = 7:3, length 17 cm, fraction 30 mL, R_f_ = 0.19). Colorless solid, mp 49.3 °C, yield 434 mg (96%). C_16_H_24_O_3_, M_r_ = 264.4. MS (EI): *m*/*z* [%] = 265 (M + H, 4), 247 (M + H-H_2_O, 10), 235 (M-CH_2_CH_3_, 18), 113 (C_7_H_13_O, 100), 105 (Ph-CO, 45). IR (neat): $\tilde{\nu }$ [cm^−1^] = 3368 (O-H), 2927, 2866 (C-H), 767, 704 (arom. monosubst.). ^1^H NMR (CDCl_3_): δ [ppm] = 0.80 (t, J = 7.5 Hz, 3 H, diox-CH_2_-C*H*_3_), 1.25–1.30 (m, 1 H, 5-H_eq_), 1.38–1.51 (m, 2 H, diox-(C*H*_2_)_3_), 1.58–1.70 (m, 5 H, 5-H_ax_, diox-(C*H*_2_)_3_), 1.74 (q, J = 7.4 Hz, 2 H, diox-C*H*_2_-CH_3_), 3.62–3.67 (m, 1 H, 4-H_ax_), 3.69 (t, J = 6.2 Hz, 2 H, C*H*_2_-OH), 3.77 (td, J = 12.0/2.5 Hz, 1 H, 6-H_ax_), 3.87 (ddd, J = 11.4/5.1/1.3 Hz, 1 H, 6-H_eq_), 7.27–7.40 (m, 5 H, Ar-H). A signal for the O*H* proton is not seen in the spectrum. HPLC (method ACN): purity 99.9%, t_R_ = 19.60 min.

***cis*-4-(4-Azidobutyl)-2-phenyl-1,3-dioxane (13a).**

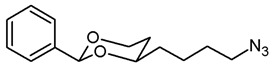


The alcohol **12a** (100 mg, 0.42 mmol) was dissolved in dry toluene (molecular sieves 4 Å, 4 mL). Zn(N_3_)_2_ · 2 pyridine ^80^ (97 mg, 0.315 mmol) and PPh_3_ (220 mg, 0.84 mmol) were added followed by a small amount of toluene (2 mL). Under N_2_ atmosphere, diisopropyl azodicarboxylate (0.16 mL, 0.84 mmol) was added dropwise and the mixture was stirred at rt for 5 h. The mixture was filtered through Celite^®^, the solvent was removed in vacuo, and the residue was purified by flash column chromatography (Ø 3 cm, cyclohexane:ethyl acetate = 19:1, length 18 cm, fraction 10 mL, R_f_ = 0.18). Colorless oil, yield 74 mg (67%). C_14_H_19_N_3_O_2_, M_r_ = 261.3. MS (ESI): *m*/*z* [%] = 284 (M + Na, 82), 545 (2 × M + Na, 32). IR (neat): $\tilde{\nu }$ [cm^−1^] = 2944, 2860 (C-H), 2091 (N_3_), 1105 (C-O), 751, 698 (arom. monosubst.). ^1^H NMR (CDCl_3_): δ [ppm] = 1.42–1.73 (m, 7 H, 5-H_eq_, diox-(C*H*_2_)_3_), 1.82 (tdd, J = 12.9/11.3/5.0 Hz, 1 H, 5-H_ax_), 3.29 (t, J = 6.6 Hz, 2 H, C*H*_2_-N_3_), 3.80–3.87 (m, 1 H, 4-H_ax_), 3.96 (td, J = 12.0/2.7 Hz, 1 H, 6-H_ax_), 4.27 (ddd, J = 11.4/5.0/1.1 Hz, 1 H, 6-H_eq_), 5.50 (s, 1 H, 2-H_ax_), 7.30–7.38 (m, 3 H, Ar-H), 7.47–7.50 (m, 2 H, Ar-H). HPLC (method ACN): purity 96.5%, t_R_ = 21.75 min.

**Azidobutyl-2-ethyl-2-phenyl-1,3-dioxane (13b).**

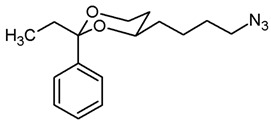


The alcohol **12b** (120 mg, 0.45 mmol) was dissolved in dry toluene (molecular sieves 4 Å, 7 mL). Zn(N_3_)_2_·2 pyridine ^80^ (97 mg, 0.315 mmol) and PPh_3_ (220 mg, 0.84 mmol) were added followed by a small amount of toluene (2 mL). Under N_2_ atmosphere, diisopropyl azodicarboxylate (0.18 mL, 0.91 mmol) was added dropwise and the mixture was stirred at rt for 5 h. The mixture was filtered through Celite^®^, the solvent was removed in vacuo, and the residue was purified by flash column chromatography (Ø 3.5 cm, cyclohexane:ethyl acetate = 19:1, length 20 cm, fraction 20 mL, R_f_ = 0.19). Colorless oil, yield 76 mg (58%). C_16_H_23_N_3_O_2_, M_r_ = 289.4. MS (EM, APCI): *m*/*z* = calculated for C_16_H_24_N_3_O_2_ 290.1863, found 290.1933. IR (neat): $\tilde{\nu }$ [cm^−1^] = 2940, 2867 (C-H), 2091 (N_3_), 58, 705 (arom. monosubst.). ^1^H NMR (CDCl_3_): δ [ppm] = 0.80 (t, J = 7.5 Hz, 3 H, diox-CH_2_-C*H*_3_), 1.24–1.29 (m, 1 H, 5-H_eq_), 1.40–1.70 (m, 7 H, 5-H_ax_, diox-(C*H*_2_)_3_), 1.74 (q, J = 7.4 Hz, 2 H, diox-C*H*_2_-CH_3_), 3.31 (t, J = 6.6 Hz, 2 H, C*H*_2_-N_3_), 3.61–3.68 (m, 1 H, 4-H_ax_), 3.77 (td, J = 11.5/2.4 Hz, 1 H, 6-H_ax_), 3.88 (ddd, J = 11.4/5.1/1.4 Hz, 1 H, 6-H_eq_), 7.26–7.41 (m, 5 H, Ar-H). HPLC (method ACN): purity 99.7%, t_R_ = 23.72 min.

**4-(*cis*-2-Phenyl-1,3-dioxan-4-yl)butan-1-amine (14a).**

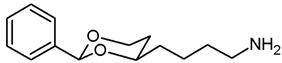


In a 25 mL Schlenk flask, the azide **13a** (64 mg, 0.24 mmol) was dissolved in dry ethyl acetate (molecular sieves 4 Å, 10 mL). Pd/C (10% Pd, 7 mg, 11% *m*/*m*) was added. The mixture was reacted with H_2_ at atmospheric pressure (using a rubber balloon). After 2 h, the H_2_ balloon was totally deflated and refilled with H_2_. The reaction was continued for additional 3 h. Afterwards, the mixture was filtered through Celite^®^. To eluate the basic amine from Celite^®^, it was necessary to wash with a basic eluent (see fc). The filtrate was concentrated in vacuo, and the residue was purified by flash column chromatography (Ø 2 cm, NH_3_ (16 mL) + MeOH (100 mL) + CH_2_Cl_2_ (ad 2000 mL), length 18 cm, fraction 10 mL, R_f_ = 0.06). Colorless oil, yield 47 mg (84%). C_14_H_21_NO_2_, M_r_ = 235.3. MS (EM, ESI): *m*/*z* = calculated for C_14_H_22_NO_2_ 236.1645, found 236.1645. IR (neat): $\tilde{\nu }$ [cm^−1^] = 3303, 3034, 2931 (N-H, C-H), 1104 (C-O), 749, 697 (arom. monosubst.). ^1^H NMR (CDCl_3_): δ [ppm] = 1.40–1.73 (m, 9 H, 5-H_eq_, diox-(C*H*_2_)_3_, N*H*_2_), 1.81 (tdd, J = 12.5/11.4/5.0 Hz, 1 H, 5-H_ax_), 2.71 (t, J = 6.6 Hz, 2 H, C*H*_2_-NH_2_), 3.80–3.86 (m, 1 H, 4-H_ax_), 3.95 (td, J = 12.0/2.5 Hz, 1 H, 6-H_ax_), 4.27 (ddd, J = 11.3/4.9/1.0 Hz, 1 H, 6-H_eq_), 5.50 (s, 1 H, 2-H_ax_), 7.29–7.38 (m, 3 H, Ar-H), 7.48–7.50 (m, 2 H, Ar-H). HPLC (method ACN): purity 99.6%, t_R_ = 12.77 min.

**4-(*trans*-2-Ethyl-2-phenyl-1,3-dioxan-4-yl)butan-1-amine (14b).**

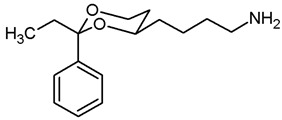


In a 25 mL Schlenk flask the azide **13b** (65 mg, 0.22 mmol) was dissolved in dry ethyl acetate (molecular sieves 4 Å, 10 mL). Pd/C (10% Pd, 6.5 mg, 10% *m*/*m*) was added. The mixture was reacted with H_2_ at atmospheric pressure (using a rubber balloon). After 2 h and after 3 h, the H_2_ balloon was totally deflated and refilled with H_2_. The reaction was stopped after 4.5 h by filtration through Celite^®^. To eluate the basic amine from Celite^®^, it was necessary to wash with a basic eluent (see fc). The filtrate was concentrated in vacuo, and the residue was purified by flash column chromatography (Ø 2 cm, NH_3_ (16 mL) + MeOH (100 mL) + CH_2_Cl_2_ (ad 2000 mL), length 18 cm, fraction 10 mL, R_f_ = 0.11). Colorless solid, mp 83.0 °C, yield 37 mg (63%). C_16_H_25_NO_2_, M_r_ = 263.4. MS (EM, ESI): *m*/*z* = calculated for C_16_H_24_NO_2_ 264.1958, found 264.1963. IR (neat): $\tilde{\nu }$ [cm^−1^] = 2972, 2935, 2864 (C-H), 760, 706 (arom. monosubst.) ^1^H NMR (CDCl_3_): δ [ppm] = 0.79 (t, J = 7.5 Hz, 3 H, diox-CH_2_-C*H*_3_), 1.24–1.30 (m, 1 H, 5-H_eq_), 1.35–1.73 (m, 9 H, 5-H_ax_, diox-(C*H*_2_)_3_, N*H*_2_), 1.74 (q, J = 7.5 Hz, 2 H, diox-C*H*_2_-CH_3_), 2.73 (t, J = 6.7 Hz, 2 H, C*H*_2_-NH_2_), 3.59–3.68 (m, 1 H, 4-H_ax_), 3.76 (td, J = 11.5/2.5 Hz, 1 H, 6-H_ax_), 3.87 (ddd, J = 11.4/5.2/1.4 Hz, 1 H, 6-H_ax_), 7.27–7.41 (m, 5 H, Ar-H). HPLC (method ACN): purity 98.3%, t_R_ = 15.40 min.

**[4-(*cis*-2-Phenyl-1,3-dioxan-4-yl)butyl]methanesulfonate (15a).**

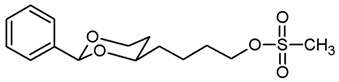


Under N_2_ atmosphere, the alcohol **12a** (451 mg, 1.91 mmol) was dissolved in CH_2_Cl_2_ abs. (30 mL) and treated with NEt_3_ (0.79 mL, 5.73 mmol). The mixture was cooled down to 0 °C. At this temperature, methanesulfonyl chloride (0.22 mL, 2.87 mmol) was added dropwise and the mixture was stirred at rt for 4 h. Then, CH_2_Cl_2_ (30 mL) was added, and the mixture was washed with 0.5 M NaOH (2 × 25 mL) and with saturated aqueous solution of NH_4_Cl (1 × 25 mL). The organic layer was dried (K_2_CO_3_). The solvent was removed in vacuo, and the residue was purified by flash column chromatography (Ø 4 cm, cyclohexane:ethyl acetate = 7:3, length 18 cm, fraction 30 mL, R_f_ = 0.21). Colorless oil, yield 578 mg (96%). C_15_H_22_O_5_S, M_r_ = 314.4. MS (EI): *m*/*z* [%] = 314 (M, 41), 105 (PhCO, 37), 91 (PhCH_2_, 35). IR (neat): $\tilde{\nu }$ [cm^−1^] = 2924, 2856 (C-H), 1350, 1170 (RO-SO_2_R), 1104 (C-O), 752, 699 (arom. monosubst.). ^1^H NMR (CDCl_3_): δ [ppm] = 1.50–1.87 (m, 8 H, 5-H_ax+eq_, diox-(C*H*_2_)_3_), 2.98 (s, 3 H, SO_2_C*H*_3_), 3.81–3.87 (m, 1 H, 4-H_ax_), 3.96 (td, J = 12.0/2.6 Hz, 1 H, 6-H_ax_), 4.24 (t, J = 6.5 Hz, 2 H, C*H*_2_-OSO_2_CH_3_), 4.27 (ddd, J = 11.4/5.0/1.2 Hz, 1 H, 6-H_eq_), 5.50 (s, 1 H, 2-H_ax_), 7.30–7.38 (m, 3 H, Ar-H), 7.47–7.49 (m, 2 H, Ar-H). HPLC (method ACN): purity 97.4%, t_R_ = 19.72 min.

**[4-(*trans*-2-Ethyl-2-phenyl-1,3-dioxan-4-yl)butyl] methanesulfonate (15b).**

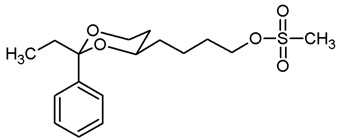


Under N_2_ atmosphere, the alcohol **12b** (250 mg, 0.95 mmol) was dissolved in CH_2_Cl_2_ abs. (30 mL) and treated with NEt_3_ (0.40 mL, 2.85 mmol). The mixture was cooled down to 0 °C. At this temperature, methanesulfonyl chloride (0.11 mL, 1.43 mmol) was added dropwise and the mixture was stirred at rt for 4 h. Then, CH_2_Cl_2_ (30 mL) was added, and the mixture was washed with 0.5 M NaOH (2 × 25 mL) and with saturated aqueous solution of NH_4_Cl (1 × 25 mL). The organic layer was dried (K_2_CO_3_). The solvent was removed in vacuo, and the residue was purified by flash column chromatography (Ø 3 cm, cyclohexane:ethyl acetate = 8:2, length 17 cm, fraction 20 mL, R_f_ = 0.11). Colorless oil, yield 314 mg (97%). C_17_H_26_O_5_S, M_r_ = 342.5. MS (EI): *m*/*z* [%] = 343 (M + H, 1), 313 (M-CH_2_CH_3_, 17), 105 (PhCO, 28), 95 (100). IR (neat): $\tilde{\nu }$ [cm^−1^] = 2941, 2871 (C-H), 1352, 1171 (RO-SO_2_R), 759, 705 (arom. monosubst.). ^1^H NMR (CDCl_3_): δ [ppm] = 0.79 (t, J = 7.5 Hz, 3 H, diox-CH_2_-C*H*_3_), 1.24–1.29 (m, 1 H, 5-H_eq_), 1.42–1.84 (m, 7 H, 5-H_ax_, diox-(C*H*_2_)_3_), 1.73 (q, J = 7.5 Hz, 2 H, diox-C*H*_2_-CH_3_), 3.02 (s, 3 H, SO_2_C*H*_3_), 3.62–3.68 (m, 1 H, 4-H_ax_), 3.77 (td, J = 11.5/2.5 Hz, 1 H, 6-H_ax_), 3.88 (ddd, J = 11.4/5.1/1.4 Hz, 1 H, 6-H_eq_), 4.27 (t, J = 6.5 Hz, 2 H, C*H*_2_-O-SO_2_CH_3_), 7.27–7.41 (m, 5 H, Ar-H). HPLC (method ACN): purity 99.9%, t_R_ = 21.43 min.

***N*-Methyl-4-(*cis*-2-phenyl-1,3-dioxan-4-yl)butan-1-amine (16a) and *N*-methyl-*N*,*N*-bis[4-(*cis*-2-phenyl-1,3-dioxan-4-yl)butyl]amine (NR_3_).**

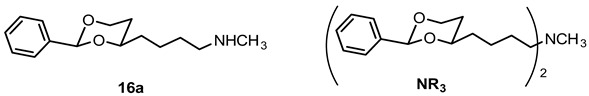


Mesylate **15a** (86 mg, 0.27 mmol) was dissolved in a solution of methylamine in ethanol (33% *m*/*m*, 8 mL). The mixture was heated to reflux for 3 h, using a reflux apparatus equipped with a rubber balloon to avoid loss of gaseous amine. Ethanol was removed under reduced pressure. The residue was dissolved in CH_2_Cl_2_ (20 mL) and washed with 1 M NaOH (2 × 10 mL). The organic layer was dried (K_2_CO_3_) and the solvent was removed in vacuo, giving a pale yellow oil which was purified by flash column chromatography (Ø 2 cm, gradient cyclohexane:ethyl acetate + 1% *N*,*N*-dimethylethanamine → ethyl acetate:MeOH 5:1 + 2.5% *N*,*N*-dimethylethanamine, length 17 cm, fraction 10 mL). The first eluent gave the dialkylated tertiary amine **NR_3_** (R_f_ = 0.26), the second eluent gave the monoalkylated secondary amine **16a** (R_f_ = 0.11).

Secondary amine **16a**: Colorless oil, yield 48 mg (70%). C_15_H_23_NO_2_, M_r_ = 249.4. MS (EM, APCI): *m*/*z* = calculated for C_15_H_24_NO_2_ 250.1802, found 250.1790. IR (neat): $\tilde{\nu }$ [cm^−1^] = 2929, 2854, 2790 (C-H), 1103 (C-O), 750, 697 (arom. monosubst.). ^1^H NMR (CDCl_3_): δ [ppm] = 1.37–1.75 (m, 7 H, 5-H_eq_, diox-(C*H*_2_)_3_)), 1.75–1.85 (m, 1 H, 5-H_ax_), 2.01 (s broad, 1 H, N*H*), 2.44 (s, 3 H, N-C*H*_3_), 2.63 (t, J = 6.9 Hz, 2 H, C*H*_2_-N-CH_3_), 3.80–3.86 (m, 1 H, 4-H_ax_), 3.95 (td, J = 11.9/2.5 Hz, 1 H, 6-H_ax_), 4.26 (dd broad, J = 11.3/4.3 Hz, 1 H, 6-H_eq_), 5.50 (s, 1 H, 2-H_ax_), 7.29–7.38 (m, 3 H, Ar-H), 7.48–7.50 (m, 2 H, Ar-H). HPLC (method ACN): purity 97.5%, t_R_ = 12.54 min. The substance was sensitive to heat and air.

Tertiary amine **NR_3_**: Colorless oil, yield 15 mg (24%). C_29_H_41_NO_4_, M_r_ = 467.6. MS (EM, ESI): *m*/*z* = calculated for C_29_H_42_NO_4_ 468.3108, found 468.3110. IR (neat): $\tilde{\nu }$ [cm^−1^] = 2941, 2854, 2786 (C-H), 1103 (C-O), 749, 696 (arom. monosubst.) ^1^H NMR (CDCl_3_): δ [ppm] = 1.36–1.74 (m, 14 H, 2 × 5-H_eq_, 2 × diox-(C*H*_2_)_3_), 1.80 (tdd, J = 12.7/11.3/5.0 Hz, 2 H, 2 × 5-H_ax_), 2.21 (s, 3 H, N-C*H*_3_), 2.33 (t, J = 7.0 Hz, 4 H, C*H*_2_-N-C*H*_2_), 3.79–3.84 (m, 2 H, 2 × 4-H_ax_), 3.95 (td, J = 12.0/2.6 Hz, 2 H, 2 × 6-H_ax_), 4.26 (dd broad, J = 11.3/4.9 Hz, 2 H, 2 × 6-H_eq_), 5.49 (s, 2 H, 2 × 2-H_ax_), 7.29–7.38 (m, 6 H, Ar-H), 7.48–7.50 (m, 4 H, Ar-H). HPLC (method ACN): purity 98.4%, t_R_ = 20.15 min.

**4-(*trans*-2-Ethyl-2-phenyl-1,3-dioxan-4-yl)-*N*-methylbutan-1-amine (16b).**

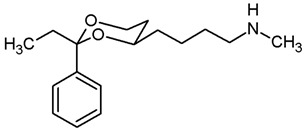


Mesylate **15b** (58 mg, 0.17 mmol) was dissolved in a solution of methylamine in ethanol (33% *m*/*m*, 5 mL). The mixture was heated to reflux for 5 h, using a reflux apparatus equipped with a rubber balloon to avoid loss of gaseous amine. Ethanol was removed under reduced pressure. The residue was dissolved in CH_2_Cl_2_ (10 mL) and washed with 1 M NaOH (2 × 10 mL). The organic layer was dried (K_2_CO_3_) and the solvent was removed in vacuo, giving a pale yellow oil which was purified by flash column chromatography (Ø 2 cm, NH_3_ (16 mL) + MeOH (100 mL) + CH_2_Cl_2_ (ad 2000 mL), length 17 cm, fraction 10 mL, R_f_ = 0.11). Colorless oil, yield 35 mg (74%). C_17_H_27_NO_2_, M_r_ = 277.4. MS (EM, ESI): *m*/*z* = calculated for C_17_H_28_NO_2_ 278.2115, found 278.2112. IR (neat): $\tilde{\nu }$ [cm^−1^] = 2928, 2863, 2788 (C-H), 757, 704 (arom. monosubst.). ^1^H NMR (CDCl_3_): δ [ppm] = 0.79 (t, J = 7.5 Hz, 3 H, diox-CH_2_-C*H*_3_), 1.25–1.29 (m, 1 H, 5-H_eq_), 1.36–1.73 (m, 8 H, 5-H_ax_, diox-(C*H*_2_)_3_, N*H*), 1.74 (q, J = 7.5 Hz, 2 H, diox-C*H*_2_-CH_3_), 2.46 (s, 3 H, N-C*H*_3_), 2.62 (t, J = 7.0 Hz, 2 H, C*H*_2_-N-CH_3_), 3.61–3.67 (m, 1 H, 4-H_ax_), 3.73–3.80 (m, 1 H, 6-H_ax_), 3.87 (ddd, J = 11.4/5.0/1.4 Hz, 1 H, 6-H_eq_), 7.26–7.40 (m, 5 H, Ar-H). HPLC (method ACN): purity 98.9%, t_R_ = 16.33 min.

***N*-Benzyl-4-(*cis*-2-phenyl-1,3-dioxan-4-yl)butan-1-amine (17a).**

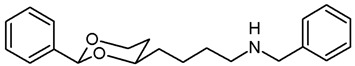


Mesylate **15a** (107 mg, 0.34 mmol) was dissolved in dry acetonitrile (molecular sieves 3 Å, 10 mL). K_2_CO_3_ (140 mg, 1.02 mmol) and freshly distilled benzylamine (48 μL, 0.44 mmol) were added, and the mixture was heated to reflux for 16 h. To complete the reaction, additional benzylamine (24 μL, 0.22 mmol) was added, and the mixture was heated to reflux for another 2 h. For workup, the solvent was removed in vacuo, and the residue was transferred into a separatory funnel with 0.1 M HCl and a small amount of ethyl acetate. The pH value was adjusted to 3–4 by adding a few drops of 0.5 M HCl. The mixture was quickly extracted with ethyl acetate. The organic layer contained small amounts of educt **15a** but no product **17a** (TLC-control) and was discarded. The aqueous layer was alkalized with 2 M NaOH and again extracted with ethyl acetate (3 × 10 mL). The combined organic layers were dried (K_2_CO_3_). The solvent was removed in vacuo, and the residue was purified by flash column chromatography (Ø 2 cm, cyclohexane:ethyl acetate = 7:3 + 1% *N*,*N*-dimethylethanamine, length 8 cm, fraction 8 mL, R_f_ = 0.09). Colorless oil, yield 36 mg (32%). C_21_H_27_NO_2_, M_r_ = 325.4. MS (EI): *m*/*z* [%] = 326 (M + H, 2), 248 (M-Ph, 4), 106 (PhCHO, 64), 91 (PHCH_2_, 100). IR (neat): $\tilde{\nu }$ [cm^−1^] = 3030, 2928, 2853 (C-H), 1105 (C-O), 746, 696 (arom. monosubst.) ^1^H NMR (CDCl_3_): δ [ppm] = 1.38–1.71 (m, 7 H, 5-H_eq_, diox-(C*H*_2_)_3_), 1.80 (tdd, J = 12.5/11.3/5.0 Hz, 1 H, 5-H_ax_), 2.65 (t, J = 6.9 Hz, 2 H, C*H*_2_-NHBz), 3.79 (s, 2 H, Ph-C*H*_2_-N), 3.80–3.85 (m, 1 H, 4-H_ax_), 3.95 (td, J = 12.9/2.6 Hz, 1 H, 6-H_ax_), 4.26 (ddd, J = 11.3/4.9/1.0 Hz, 1 H, 6-H_eq_), 5.49 (s, 1 H, 2-H_ax_), 7.22–7.38 (m, 8 H, Ar-H), 7.48–7.50 (m, 2 H, Ar-H). A signal for the N*H* proton is not seen in the spectrum. HPLC (method ACN): purity 98.2%, t_R_ = 17.69 min.

***N*-Benzyl-4-(*trans*-2-ethyl-2-phenyl-1,3-dioxan-4-yl)butan-1-amine (17b).**

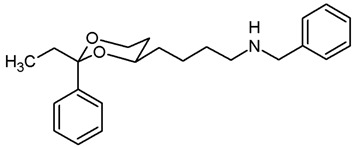


Mesylate **15b** (100 mg, 0.29 mmol) was dissolved in dry acetonitrile (molecular sieves 3 Å, 10 mL). K_2_CO_3_ (120 mg, 0.87 mmol) and freshly distilled benzylamine (48 μL, 0.44 mmol) were added, and the mixture was heated to reflux for 8 h. Additional benzylamine (48 μL, 0.44 mmol) was added, and the mixture was heated to reflux for another 17 h overnight. For workup, the solvent was removed in vacuo, and the residue was transferred into a separatory funnel with water and a small amount of ethyl acetate. The pH value was adjusted to 9–10 with 1 M NaOH and the mixture was extracted with ethyl acetate (3 × 20 mL). The combined organic layers were washed with brine (1 × 20 mL) and dried (K_2_CO_3_). The solvent was removed in vacuo, and the residue was purified by flash column chromatography (Ø 2.5 cm, cyclohexane:ethyl acetate = 8:2 + 0.5% *N*,*N*-dimethylethanamine, length 18 cm, fraction 10 mL, R_f_ = 0.15). Colorless oil, yield 84 mg (82%). C_23_H_31_NO_2_, M_r_ = 353.5. MS (EM, ESI): *m*/*z* = calculated for C_23_H_32_NO_2_ 354.2428, found 354.2424. IR (neat): $\tilde{\nu }$ [cm^−1^] = 3026, 2928, 2862 (C-H), 757, 699 (arom. monosubst.). ^1^H NMR (CDCl_3_): δ [ppm] = 0.79 (t, J = 7.5 Hz, 3 H, diox-CH_2_-C*H*_3_), 1.23–1.28 (m, 1 H, 5-H_eq_), 1.37–1.71 (m, 8 H, 5-H_ax_, diox-(C*H*_2_)_3_, N*H*), 1.73 (q, J = 7.5 Hz, 2 H, diox-C*H*_2_-CH_3_), 2.66 (t, J = 7.0 Hz, 2 H, C*H*_2_-NHBz), 3.59–3.66 (m, 1 H, 4-H_ax_), 3.73–3.79 (m, 1 H, 6-H_ax_), 3.81 (s, 2 H, N-C*H*_2_-Ph), 3.87 (ddd, J = 11.4/5.1/1.4 Hz, 1 H, 6-H_eq_), 7.23–7.39 (m, 10 H, Ar-H). HPLC (method ACN): purity 99.2%, t_R_ = 19.16 min.

***N*-Benzyl-*N*-methyl-4-(*cis*-2-phenyl-1,3-dioxan-4-yl)butan-1-amine (18a).**

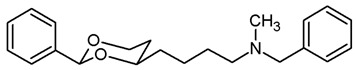


Mesylate **15a** (80 mg, 0.25 mmol) was dissolved in dry acetonitrile (molecular sieves 3 Å, 10 mL). K_2_CO_3_ (104 mg, 0.75 mmol) and *N*-methylbenzylamine (48 μL, 0.44 mmol) were added, and the mixture was heated to reflux for 7 h. To complete the transformation, additional *N*-methylbenzylamine (48 μL, 0.44 mmol) was added, and the mixture was heated to reflux for another 16 h. For workup, the solvent was removed in vacuo, and the residue was transferred into a separatory funnel with water and ethyl acetate. The pH value was adjusted to 9–10 with 2 M NaOH. The mixture was extracted with ethyl acetate (3 × 10 mL). The combined organic layers were dried (K_2_CO_3_), the solvent was removed in vacuo, and the residue was purified by flash column chromatography (Ø 2 cm, gradient CH_2_Cl_2_:MeOH = 97.5:2.5 → CH_2_Cl_2_:MeOH = 95:5, length 19 cm, fraction 10 mL, R_f_ = 0.30 (CH_2_Cl_2_:MeOH = 95:5)). Colorless oil, yield 68 mg (80%). C_22_H_29_NO_2_, M_r_ = 339.5. MS (EM, APCI): *m*/*z* = calculated for C_22_H_30_NO_2_ 340.2271, found 340.2313. IR (neat): $\tilde{\nu }$ [cm^−1^] = 2941, 2842, 2785 (C-H), 740, 696 (arom. monosubst). ^1^H NMR (CDCl_3_): δ [ppm] = 1.36–1.72 (m, 7 H, 5-H_eq_, diox-(C*H*_2_)_3_), 1.80 (tdd, J = 12.7/11.3/5.0 Hz, 1 H, 5-H_ax_), 2.20 (s, 3 H, N-C*H*_3_), 2.39 (t, J = 7.0 Hz, 2 H, diox-(CH_2_)_3_-C*H*_2_-N), 3.49 (s, 2 H, N-C*H*_2_-Ph), 3.79–3.85 (m, 1 H, 4-H_ax_), 3.95 (td, J = 12.0/2.6 Hz, 1 H, 6-H_ax_), 4.27 (ddd, J = 11.3/4.9/0.9 Hz, 1 H, 6-H_eq_), 5.50 (s, 1 H, 2-H_ax_), 7.22–7.38 (m, 8 H, Ar-H), 7.49–7.50 (m, 2 H, Ar-H). HPLC (method ACN): purity 98.3%, t_R_ = 18.33 min.

***N*-Benzyl-4-(*trans*-2-ethyl-2-phenyl-1,3-dioxan-4-yl)-*N*-methyl-butan-1-amine (18b).**

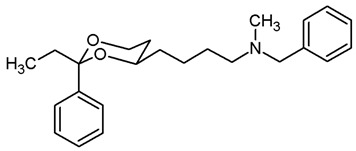


Benzylamine **17b** (50 mg, 0.14 mmol) was dissolved in CH_2_Cl_2_ abs. (7 mL). A solution of formalin (37%, stab. with 10–15% MeOH, 210 μL, 2.8 mmol) and NaBH(OAc)_3_ (95%, 62.5 mg, 0.28 mmol) were added, and the mixture was stirred at rt overnight. CH_2_Cl_2_ (10 mL) and water (15 mL) were added, and the aqueous layer was extracted with CH_2_Cl_2_ (3 × 10 mL). The combined organic layers were washed with brine (1 × 15 mL) and dried (K_2_CO_3_). The solvent was removed in vacuo, and the residue was purified by flash column chromatography (Ø 2 cm, CH_2_Cl_2_:MeOH = 96:4, length 20 cm, fraction 10 mL, R_f_ = 0.14). Colorless resin, yield 47 mg (91%). C_24_H_33_NO_2_, M_r_ = 367.5. MS (EM, APCI): *m*/*z* = calculated for C_24_H_34_NO_2_ 368.2584, found 368.2625. IR (neat): $\tilde{\nu }$ [cm^−1^] = 2940, 2865, 2786 (C-H), 757, 699 (arom. monosubst.). ^1^H NMR (CDCl_3_): δ [ppm] = 0.80 (t, J = 7.5 Hz, 3 H, diox-CH_2_-C*H*_3_), 1.26 (d broad, J = 12.8 Hz, 1 H, 5-H_eq_), 1.50–1.69 (m, 7 H, 5-H_ax_, diox-(C*H*_2_)_3_), 1.74 (q, J = 7.4 Hz, 2 H, diox-C*H*_2_-CH_3_), 2.23 (s, 3 H, N-C*H*_3_), 2.40–2.46 (m, 2 H, diox-(CH_2_)_3_-C*H*_2_-N), 3.53 (s broad, 2 H, N-C*H*_2_-Ph), 3.59–3.66 (m, 1 H, 4-H_ax_), 3.76 (td, J = 12.0/2.5 Hz, 1 H, 6-H_ax_), 3.87 (dd broad, J = 11.3/4.9 Hz, 1 H, 6-H_eq_), 7.23–7.40 (m, 10 H, Ar-H). HPLC (method ACN): purity 99.5%, t_R_ = 20.01 min.

**1-[4-(*cis*-2-Phenyl-1,3-dioxan-4-yl)butyl]pyrrolidine (19a).**

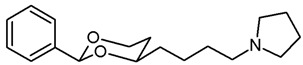


Mesylate **15a** (80 mg, 0.25 mmol) was dissolved in dry acetonitrile (molecular sieves 3 Å, 10 mL). K_2_CO_3_ (104 mg, 0.75 mL) and freshly distilled pyrrolidine (31 μL, 0.375 mmol) were added, and the mixture was heated to reflux for 14.5 h. For workup, the solvent was removed in vacuo, and the residue was transferred into a separatory funnel with water and a small amount of ethyl acetate. The pH value was adjusted to 9-10 with 2 M NaOH. The mixture was extracted with ethyl acetate (3 × 20 mL) and with CH_2_Cl_2_ (3 × 20 mL). The combined organic layers were dried (K_2_CO_3_). The solvent was removed in vacuo, and the residue was purified by flash column chromatography (Ø 2 cm, NH_3_ (16 mL) + MeOH (100 mL) + CH_2_Cl_2_ (ad 2000 mL), length 18 cm, fraction 8 mL, R_f_ = 0.12). The eluent was prepared as follows: In a 2 L volumetric flask, MeOH (100 mL) and NH_3_ (16 mL) were mixed, CH_2_Cl_2_ (1.5 L) was added, and the mixture was gently swirled. The mixture cooled down by itself. It was warmed up to rt and then diluted with CH_2_Cl_2_ to a total volume of 2 L. Colorless solid, mp 50–51 °C, yield 53 mg (81%). C_18_H_27_NO_2_, M_r_ = 289.4. MS (EI): *m*/*z* [%] = 288 (M-1, 10), 84 (CH_2_=N(CH_2_-CH_2_)_2_, 100).

IR (neat): $\tilde{\nu }$ [cm^−1^] = 2925, 2859, 2786 (C-H), 1105 (C-O), 762, 701 (arom. monosubst.).

^1^H NMR (CDCl_3_): δ [ppm] = 1.38–1.85 (m, 12 H, 5-H_ax+eq_, diox-(C*H*_2_)_3_, N(CH_2_-C*H*_2_)_2_), 2.47 (t, J = 7.5 Hz, 2 H, C*H*_2_-N_pyrrolidine_), 2.50–2.55 (m, 4 H, N-(C*H*_2_-CH_2_)_2_), 3.80–3.86 (m, 1 H, 4-H_ax_), 3.95 (td, J = 12.2/2.6 Hz, 1 H, 6-H_ax_), 4.24 (ddd, J = 11.3/4.9/1.0 Hz, 1 H, 6-H_eq_), 5.49 (s, 1 H, 2-H_ax_), 7.29–7.38 (m, 3 H, Ar-H), 7.48–7.50 (m, 2 H, Ar-H). HPLC (method ACN): purity 98.0%, t_R_ = 15.07 min.

**1-[4-(*trans*-2-Ethyl-2-phenyl-1,3-dioxan-4-yl)butyl]pyrrolidine (19b).**

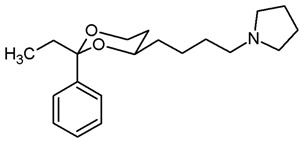


Mesylate **15b** (100 mg, 0.29 mmol) was dissolved in dry acetonitrile (molecular sieves 3 Å, 10 mL). K_2_CO_3_ (120 mg, 0.87 mL) and freshly distilled pyrrolidine (46 μL, 0.435 mmol) were added, and the mixture was heated to reflux for 17 h. For workup, the solvent was removed in vacuo, and the residue was transferred into a separatory funnel with water and a small amount of ethyl acetate. The pH value was adjusted to 9–10 with 2 M NaOH. The mixture was extracted with ethyl acetate (3 × 20 mL). The combined organic layers were washed with brine (1 × 20 mL) and dried (K_2_CO_3_). The solvent was removed in vacuo, and the residue was purified by flash column chromatography (Ø 2 cm, cyclohexane:ethyl acetate = 8:2 + 0.5% *N*,*N*-dimethylethanamine, length 18 cm, fraction 10 mL, R_f_ = 0.11 (cyclohexane:ethyl acetate = 7:3 + 0.5% *N*,*N*-dimethylethanamine)). Colorless oil, yield 86 mg (94%). C_20_H_31_NO_2_, M_r_ = 317.5. MS (EI): *m*/*z* [%] = 318 (M + H, 34), 84 (CH_2_=N(CH_2_-CH_2_)_2_, 100). IR (neat): $\tilde{\nu }$ [cm^−1^] = 2930, 2866, 2784 (C-H), 757, 704 (arom. monosubst.). ^1^H NMR (CDCl_3_): δ [ppm] = 0.79 (t, J = 7.5 Hz, 3 H, diox-CH_2_-C*H*_3_), 1.25–1.29 (m, 1H, 5-H_eq_), 1.33–1.83 (m, 11 H, 5-H_ax_, diox-(C*H*_2_)_3_, N(CH_2_-C*H*_2_)_2_), 1.73 (q, J = 7.5 Hz, 2 H, diox-C*H*_2_-CH_3_), 2.45–2.49 (m, 2 H, C*H*_2_-N_pyrrolidine_), 2.50–2.55 (m, 4 H, N(C*H*_2_-CH_2_)_2_), 3.60–3.67 (m, 1 H, 4-H_ax_), 3.73–3.79 (m, 1 H, 6-H_ax_), 3.87 (ddd, J = 11.3/5.0/1.3 Hz, 1 H, 6-H_eq_), 7.26–7.40 (m, 5 H, Ar-H). HPLC (method ACN): purity 99.7%, t_R_ = 17.47 min.

**3-Hydroxy-1-phenyl-4-(*cis*-2-phenyl-1,3-dioxan-4-yl)butan-1-one (20a).**

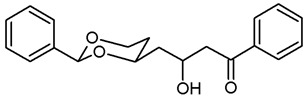


Under N_2_ atmosphere, iPr_2_NH (1.12 mL, 8 mmol) was dissolved in THF abs. (12 mL) and cooled to −78 °C. A solution of *n*-butyllithium (1.6 M in *n*-hexane, 5 mL, 8 mmol) was added dropwise and the mixture was stirred for 15 min at −78 °C. Acetophenone (1.21 mL, 10.4 mmol), dissolved in THF abs. (6 mL), was added dropwise. 5 min later, a solution of the aldehyde **9a** (827 mg, 4 mmol) in THF abs. (6 mL) was added, and the reaction mixture was stirred for 45 min at −78 °C. Saturated aqueous solution of NH_4_Cl (25 mL) was added, and the cooling bath was removed, allowing the mixture to warm up. At rt, the reaction mixture was diluted with water (30 mL) and extracted with ethyl acetate (3 × 30 mL). The combined organic layers were washed with brine (1 × 30 mL), dried (K_2_CO_3_) and the solvent was removed in vacuo. The residue was purified by flash column chromatography (Ø 7 cm, cyclohexane:ethyl acetate = 7:3, length 20 cm, fraction 65 mL, R_f_ = 0.15) to give a solid, which was recrystallized from cyclohexane with a few drops of ethyl acetate. Colorless crystals, mp 82–83 °C, yield 905 mg (69%). C_20_H_22_O_4_ (M_r_ = 326.4). MS (ESI): *m*/*z* [%] = 675 (2 × M + Na, 100). IR (neat): $\tilde{\nu }$ [cm^−1^] = 3492 (O-H) 2923, 2858 (C-H), 1676 (C=O), 1099 (C-O), 749, 689 (arom. monosubst.). ^1^H NMR (CDCl_3_): δ [ppm] = 1.56 (dtd, J = 13.3/2.4/1.3 Hz, 0.6H, 5-H_eq_), 1.62 (dtd, J = 13.3/2.3/1.4 Hz, 0.4H, 5-H_eq_), 1.81–2.01 (m, 3H, 5-H_ax_ and diox-C*H*_2_-CH-OH), 3.09 (dd, J = 17.7/8.8 Hz, 0.6H, C*H*_2_-C=O), 3.15 (dd, J = 17.3/4.5 Hz, 0.4H, C*H*_2_-C=O), 3.25 (dd, J = 17.7/2.9 Hz, 0.6H, C*H*_2_-C=O), 3.26 (dd, J = 17.3/7.7 Hz, 0.4H, C*H*_2_-C=O), 3.56–3.57 (m, 1H, CHO*H*), 4.01 (td, J = 11.8/2.5 Hz, 1H, 6-H_ax_), 4.20–4.20 (m, 1H, C*H*OH), 4.29 (ddd, J = 11.4/5.0/1.1 Hz, 1H, 6-H_eq_), 4.49–4.60 (m, 1 H, 4-H_ax_), 5.57 (s, 1 H, 2-H_ax_), 7.32–7.60 (m, 8 H, Ar-H), 7.92–7.96 (m, 2 H, Ar-H). The ratio of diastereomer is 60: 40. HPLC (method ACN): purity 99.6%, t_R_ = 19.87 min.

**4-(*trans*-2-Ethyl-2-phenyl-1,3-dioxan-4-yl)-3-hydroxy-1-phenylbutan-1-one (20b).**

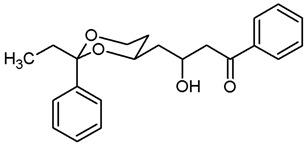


Under N_2_ atmosphere, iPr_2_NH (2.05 mL, 14.52 mmol) was dissolved in THF abs. (22 mL) and cooled to −78 °C. A solution of *n*-butyllithium (1.6 M in *n*-hexane, 9.10 mL, 14.52 mmol) was added dropwise and the mixture was stirred for 15 min at −78 °C. Acetophenone (2.20 mL, 18.88 mmol), dissolved in THF abs. (11 mL), was added dropwise. 5 min later, a solution of the aldehyde **9b** (1.70 g, 7.26 mmol) in THF abs. (11 mL) was added, and the reaction mixture was stirred for 80 min at −78 °C. Saturated aqueous solution of NH_4_Cl (50 mL) was added, and the cooling bath was removed, allowing the mixture to warm up. At rt, the reaction mixture was diluted with water (enough to dissolve all solids) and extracted with ethyl acetate (3 × 30 mL). The combined organic layers were washed with brine (1 × 30 mL), dried (K_2_CO_3_) and the solvent was removed in vacuo. Purification by flash column chromatography (Ø 6 cm, cyclohexane:ethyl acetate = 9:1, length 17 cm, fraction 65 mL, R_f_ = 0.09) gave a mixture of two diastereomers. Colorless solid, mp 122–129 °C, yield 2.16 g (84%). One of the diastereomers was isolated by recrystallisation from cyclohexane:ethyl acetate 95:5. Colorless crystals, mp 132 °C, yield 61% related to the fc isolated product. C_22_H_26_O_4_, M_r_ = 354.4. MS (EI): *m*/*z* [%] = 377 (M + Na, 100). IR (neat): $\tilde{\nu }$ [cm^−1^] = 3530 (O-H), 3060, 2956, 2882 (C-H), 1669 (C=O), 752, 688 (arom. monosubst.). ^1^H NMR (CDCl_3_): δ [ppm] = 0.79 (t, J = 7.5 Hz, 3 × 0.7 H, diox-CH_2_-C*H*_3_), 0.80 (t, J = 7.5 Hz, 3 × 0.7 H, diox-CH_2_-C*H*_3_), 1.26–1.39 (m, 1 H, 5-H_eq_), 1.71–1.89 (m, 5 H, diox-C*H*_2_-CH_3_, 5-H_ax_, diox-C*H*_2_-COH), 3.12 (dd, J = 17.0/5.3 Hz, 0.3 H, C*H*_2_-CO), 3.15 (dd, J = 17.7/8.3 Hz, 0.7 H, C*H*_2_-CO), 3.26 (dd, J = 17.6/3.5 Hz, 0.7 H, C*H*_2_-CO), 3.29 (dd, J = 17.0/7.0 Hz, 0.3 Hz, C*H*_2_-CO), 3.58–3.59 (m, 1 H, O-*H*), 3.77–3.92 (m, 2 H, 6-H_ax+eq_), 4.03–4.13 (m, 1 H, 4-H_ax_), 4.53–4.62 (m, 0.3 H, C*H*-OH), 4.64–4.73 (m, 0.7 H, C*H*-OH), 7.28–7.63 (m, 8 H, Ar-H), 7.97–8.00 (m, 2 H, Ar-H). The ratio of diastereomers is 70:30. HPLC (method ACN): purity 95.7%, t_R_ = 21.00 min.

**(2*Z*)-1-Phenyl-4-(*cis*-2-phenyl-1,3-dioxan-4-yl)but-2-en-1-one ((*Z*)-21a) and** **(2*E*)-1-Phenyl-4-(*cis*-2-phenyl-1,3-dioxan-4-yl)but-2-en-1-one ((*E*)-21a) and (3*E*)-1-Phenyl-4-(*cis*-2-phenyl-1,3-dioxan-4-yl)but-3-en-1-one (A).**

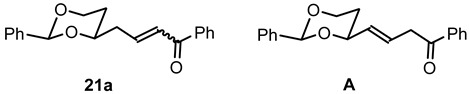


Under N_2_ atmosphere, the β-hydroxyketone **20a** (600 mg, 1.84 mmol) was dissolved in CH_2_Cl_2_ abs. (9 mL) and treated with NEt_3_ (1.54 mL, 11.04 mmol). The mixture was cooled to 0 °C. A solution of methanesulfonyl chloride (280 μL, 3.68 mmol) in CH_2_Cl_2_ abs. (1 mL) was added dropwise and the reaction mixture was stirred for 2 h at 0 °C. Water (10 mL) was added, and the organic layer was separated. The aqueous layer was extracted with CH_2_Cl_2_ (3 × 10 mL). The combined organic layers were washed with 1 M HCl (2 × 20 mL) and with saturated aqueous solution of NaHCO_3_ (2 × 20 mL) and were dried (K_2_CO_3_). The solvent was removed in vacuo, and the residue was purified by flash column chromatography (Ø 4 cm, cyclohexane:ethyl acetate = 9:1, length 18 cm, fraction 30 mL).

(*Z*)-**21a** (R_f_ = 0.15): Colorless oil, yield 27 mg (2.3%). C_20_H_20_O_3_, M_r_ = 308.4. ^1^H NMR (CDCl_3_): δ [ppm] = 1.56–1.62 (m, 1 H, 5-H_eq_), 1.92 (qd, J = 12.5/5.0 Hz, m, 1 H, 5-H_ax_), 2.87–2.95 (m, 1 H, C*H*_2_-CH=CH), 3.06 (dddd, J = 15.8/7.4/4.3/1.7 Hz, 1 H, C*H*_2_-CH=CH), 3.98 (td, J = 12.0/2.7 Hz, 1 H, 6-H_ax_), 4.01–4.08 (m, 1 H, 4-H_ax_), 4.28 (ddd, J = 11.5/4.1/1.0 Hz, 1 H, 6-H_eq_), 5.54 (s, 1 H, 2-H_ax_), 6.57 (dt, J = 11.7/7.2 Hz, 1 H, C*H*=CH-CO), 6.95 (dt, 11.7/1.6 Hz, 1 H, CH=C*H*-C=O), 7.33–7.58 (m, 8 H, Ar-H), 7.94–7.96 (m, 2 H, Ar-H).

(*E*)-**21a** (R_f_ = 0.08): Colorless oil, yield 474 mg (84%). C_20_H_20_O_3_, M_r_ = 308.4. MS (ESI): *m*/*z* [%] = 331 (M + Na, 27), 639 (2 × M + Na, 100), 655 (2 × M + K, 76). IR (neat): ν [cm^−1^] = 3062, 2921, 2853 (C-H), 1669 (C=O), 1620 (C=C), 1100 (C-O), 749, 594 (arom. monosubst.). **^1^**H NMR (CDCl_3_): δ [ppm] = 1.61 (dtd, J = 13.3/2.5/1.4 Hz, 1 H, 5-H_eq_), 1.90 (dddd, J = 13.2/12.4/11.3/5.1 Hz, 1 H, 5-H_ax_), 2.59 (dddd, J = 14.9/6.8/5.6/0.9 Hz, 1 H, C*H*_2_-CH=CH), 2.72 (dtd, J = 15.0/6.9/1.2 Hz, 1 H, C*H*_2_-CH=CH), 3.97 (td, J = 12.0/2.6 Hz, 1 H, 6-H_ax_), 4.03–4.09 (m, 1 H, 4-H_ax_), 4.30 (ddd, J = 11.5/5.0/1.3 Hz, 1 H, 6-H_eq_), 5.52 (s, 1 H, 2-H_ax_), 6.91 (dt, J = 15.5/1.0, 1 H, CH=C*H*-CO), 7.10 (dt, J = 15.4/7.1 Hz, 1 H, C*H*=CH-CO), 7.31–7.59 (m, 8 H, Ar-H), 7.92–7.94 (m, 2 H, Ar-H). HPLC (method ACN): purity 96.4%, t_R_ = 21.63 min.

**A** (R_f_ = 0.12): Colorless oil, yield 29 mg (2.6%). C_20_H_20_O_3_, M_r_ = 308.4. **^1^**H NMR (CDCl_3_): δ [ppm] = 1.63 (dtd, J = 13.3/2.5/1.5 Hz, 1 H, 5-H_eq_), 1.97 (dddd, J = 13.1/12.4/11.4/5.0 Hz, 1 H, 5-H_ax_), 3.76–3.79 (m, 2 H, C*H*_2_-CO), 4.00 (td, J = 12.0/2.5 Hz, 1 H, 6-H_ax_), 4.29 (ddd, J = 11.3/4.9/1.1 Hz, 1 H, 6-H_eq_), 4.40–4.45 (m, 1 H, 4-H_ax_), 5.57 (s, 1 H, 2-H_ax_), 5.76 (ddt, J = 15.7/6.1/3.1 Hz, 1 H, C*H*=CH-CH_2_-CO), 6.08 (dtd, J = 15.5/6.9/1.1 Hz, 1 H, CH=C*H*-CH_2_-CO), 7.30–7.60 (m, 8 H, Ar-H), 7.95–7.97 (m, 2 H, Ar-H).

**(2*Z*)-4-(*trans*-2-Ethyl-2-phenyl-1,3-dioxan-4-yl)-1-phenylbut-2-en-1-one ((*Z*)-21b) and (2*E*)-4-(*trans*-2-ethyl-2-phenyl-1,3-dioxan-4-yl)-1-phenylbut-2-en-1-one ((*E*)-21b).**

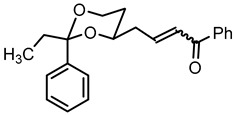


Under N_2_ atmosphere, the β-hydroxyketone **20b** (1.776 g, 5.0 mmol) was dissolved in CH_2_Cl_2_ abs. (20 mL) and treated with NEt_3_ (4.17 mL, 30.1 mmol). The mixture was cooled to 0 °C. A solution of methanesulfonyl chloride (0.78 mL, 10.0 mmol) in CH_2_Cl_2_ abs. (2 mL) was added dropwise and the reaction mixture was stirred for 2.5 h at 0 °C. Water (30 mL) was added, and the organic layer was separated. The aqueous layer was extracted with CH_2_Cl_2_ (3 × 20 mL). The combined organic layers were washed with 1 M HCl (2 × 30 mL) and with saturated aqueous solution of NaHCO_3_ (2 × 30 mL) and were dried (K_2_CO_3_). The solvent was removed in vacuo. Purification by flash column chromatography (Ø 5.5 cm, cyclohexane:ethyl acetate = 9.5:0.5, length 17 cm, fraction 65 mL) gave a mixture of (*Z*)-**21b** (R_f_ = 0.22) and (*E*)-**21b** (R_f_ = 0.15). Colorless oil, yield 1.567 g (93%). C_22_H_24_O_3_, M_r_ = 336.4. MS (ESI): *m*/*z* [%] = 337 (M + H, 45), 359 (M + Na, 100). IR (neat): $\tilde{\nu }$ [cm^−1^] = 3059, 2928, 2875 (C-H), 1671 (C=O), 1622 (C=C), 758, 695 (arom. monosubst.). ^1^H NMR (CDCl3): δ [ppm] = 0.81 (t, J = 7.5 Hz, 3 H, diox-CH_2_-C*H*_3_), 1.33–1.37 (m, 1 H, 5-H_eq_), 1.75 (q, J = 7.4 Hz, 2 H, diox-C*H*_2_-CH_3_), 1.72–1.83 (m, 1 H, 5-H_ax_), 3.45–2.52 (m, 0.87 H, C*H*_2_-CH=CH), 2.61 (dtd, J = 14.3/7.2/1.1 Hz, 0.87 H, C*H*_2_-CH=CH), 2.97–2.87 (m, 0.13 H, C*H*_2_-CH=CH), 2.90–2.97 (m, 0.13 H, C*H*_2_-CH=CH), 3.80 (td, J = 11.6/2.5 Hz, 1 H, 6-H_ax_), 3.84–3.90 (m, 1 H, 4-H_ax_), 3.91 (ddd, J = 11.5/5.0/1.4 Hz, 1 H, 6-H_eq_), 6.61 (dt, 11.7/7.2 Hz, 0.13 H, C*H*=CH-CO), 6.96 (dt, J = 15.5/1.1 Hz, 0.87 H, CH=C*H*-CO) 6.94–7.00 (m, 0.13 H, CH=C*H*-CO), 7.10 (dt, J = 15.3/7.2 Hz, 0.87 H, C*H*=CH-CO), 7.27–7.60 (m, 8 H, Ar-H), 7.94–7.97 (m, 2 H, Ar-H). The 1H NMR spectrum shows diastereomers (*E*)-**21b**:(*Z*)-**21b** in the ratio 87:13. HPLC (method ACN): 86.5%, t_R_ = 23.3 min ((*E*)-**21b**), 11.5%, t_R_ = 23.6 min ((*Z*)-**21b**).

**1-Phenyl-4-(*cis*-2-phenyl-1,3-dioxan-4-yl)butan-1-one (22a).**

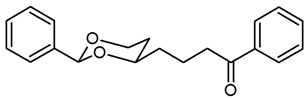


**21a** (250 mg, 0.81 mmol) was dissolved in methanol abs. (25 mL). Pd/C (10% Pd, 25 mg, 10% *m*/*m*) and ammonium formate (101 mg, 1.62 mmol) were added. After purging with N_2_, the mixture was heated to reflux for 2 h, then cooled down to rt and filtered through Celite^®^. The solvent was removed in vacuo, and the residue was purified by flash column chromatography (Ø 3 cm, cyclohexane:ethyl acetate = 9:1, length 18 cm, fraction 20 mL, R_f_ = 0.33 (cyclohexane:ethyl acetate = 8:2)). Colorless solid, mp 68.3 °C, yield 206 mg (82%). C_20_H_22_O_3_, M_r_ = 310.4. MS (ESI): *m*/*z* [%] = 643 (2 × M + Na, 100). IR (neat): $\tilde{\nu }$ [cm^−1^] = 2916, 2855 (C-H), 1672 (C=O), 1093 (C-O), 756, 691 (arom. monosubst.). ^1^H NMR (CDCl_3_): δ [ppm] = 1.53–1.58 (m, 1 H, 5-H_eq_), 1.62–2.02 (m, 5 H, diox-C*H*_2_-C*H*_2_, 5-H_ax_), 3.04 (t, J = 7.2 Hz, 2 H, C*H*_2_-CO-Ph), 3.87–3.93 (m, 1 H, 4-H_ax_), 3.97 (td, J = 11.9/2.6 Hz, 1 H, 6-H_ax_), 4.27 (ddd, J = 11.4/4.9/1.1 Hz, 1 H, 6-H_eq_), 5.52 (s, 1 H, 2-H_ax_), 7.29–7.57 (m, 8H, Ar-H), 7.94–7.96 (m, 2 H, Ar-H). HPLC (method MeOH): purity 98.8%, t_R_ = 18.61 min.

**4-(*trans*-2-Ethyl-2-phenyl-1,3-dioxan-4-yl)-1-phenylbutan-1-one (22b).**

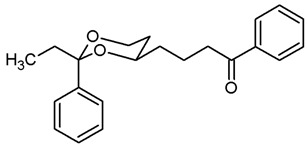


The α,β-unsaturated ketone **21b** (mixture of (*E*)-**21b** and (*Z*)-**21b**, 200 mg, 0.59 mmol) was dissolved in methanol abs. (10 mL). Pd/C (10% Pd, 20 mg, 10% *m*/*m*) and ammonium formate (73 mg, 1.18 mmol) were added. After purging with N_2_, the mixture was heated to reflux for 2 h, then cooled down to rt and filtered through Celite^®^. The solvent was removed in vacuo, and the residue was adsorbed on silica gel and given on the column (Ø 2 cm, cyclohexane:ethyl acetate = 9.5:0.5, length 18 cm, fraction 10 mL, R_f_ = 0.21). Colorless solid, mp 78–79 °C, yield 149 mg (75%). C_22_H_26_O_3_, M_r_ = 338.4. MS (ESI): *m*/*z* [%] = 361 (M + Na, 100). IR (neat): $\tilde{\nu }$ [cm^−1^] = 3060, 2940, 2871 (C-H), 1682 C=O), 756, 705 (arom. monosubst.). ^1^H NMR (CDCl_3_): δ [ppm] = 0.79 (t, J = 7.5 Hz, 3 H, diox-CH_2_-C*H*_3_), 1.27–1.32 (m, 1 H, 5-H_eq_), 1.38–1.61 (m, 3 H, 5-H_ax_, diox-C*H*_2_-CH_2_-CH_2_), 1.63 (q, J = 7.3 Hz, 2 H, diox-C*H*_2_-CH_3_), 1.70–1.96 (m, 2 H, diox-CH_2_-C*H*_2_-CH_2_), 2.93 (t, J = 7.3 Hz, 2 H, C*H*_2_-CO), 3.57–3.63 (m, 1 H, 4-H_ax_), 3.68 (td, J = 12.0/2.7 Hz, 1 H, 6-H_ax_), 3.77 (ddd, J = 11.3/5.1/1.3 Hz, 1 H, 6-H_eq_), 7.15–7.48 (m, 8 H, Ar-H), 7.86–7.88 (m, 2 H, Ar-H). HPLC (method ACN): purity 99.4%, t_R_ = 23.12 min.

***N*-Hydroxy-1-phenyl-4-(*cis*-2-phenyl-1,3-dioxan-4-yl)butan-1-imine (23a).**

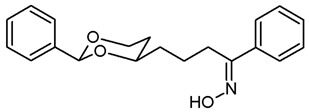


The saturated ketone **22a** (180 mg, 0.58 mmol) and hydroxylamine-HCl (180 mg, 2.6 mmol) were suspended in dry ethanol (molecular sieves 3 Å, 20 mL). NaOAc (95 mg, 1.16 mmol) was added, and the mixture was heated to reflux for 7 h. Ethanol was removed under reduced pressure, and the residue was transferred into a separatory funnel with CH_2_Cl_2_ (20 mL) and water (20 mL). The layers were separated, and the aqueous layer was extracted with CH_2_Cl_2_ (2 × 10 mL). The combined organic layers were dried (K_2_CO_3_). The solvent was removed in vacuo giving the crude product, which was purified by flash column chromatography (Ø 3 cm, cyclohexane:ethyl acetate = 9:1, length 19 cm, fraction 20 mL, R_f_ = 0.05). Colorless solid, mp 86 °C, yield 147 mg (78%). C_20_H_23_NO_3_, M_r_ = 325.4. MS (ESI): *m*/*z* [%] = 325 (M, 100), 348 (M + Na, 75), 364 (M + K, 30). IR (neat): $\tilde{\nu }$ [cm^−1^] = 3235 (O-H), 3065, 2925, 2850 (C-H), 1104 (C-O), 748, 693 (arom. monosubst). ^1^H NMR (CDCl_3_): δ [ppm] = 1.48–1.52 (m, 1 H, 5-H_eq_), 1.59–1.85 (m, 5 H, 5-H_ax_, diox-C*H*_2_-C*H*_2_), 2.81–2.92 (m, 2 H, C*H*_2_-C=N), 3.83–3.88 (m, 1 H, 4-H_ax_), 3.94 (td, J = 12.1/2.5 Hz, 1 H, 6-H_ax_), 4.25 (dd broad, J = 11.3/4.8 Hz, 1 H, 6-H_eq_), 5.49 (s, 1 H, 2-H_ax_), 7.30–7.37 (m, 6 H, Ar-H), 7.46 (dd, J = 7.7/1.7 Hz, 2 H, N=C-Ar-H_ortho_), 7.60–7.62 (m, 2 H, diox-Ar-H_ortho_), 8.45 (s, 1 H, C=N-O-*H*). HPLC (method MeOH): purity 98.5%, t_R_ = 17.83 min.

**1-Phenyl-4-(*cis*-2-phenyl-1,3-dioxan-4-yl)butan-1-amine (24a).**

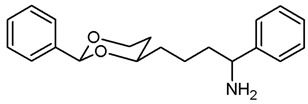


(a) Reduction in oxime **23a**

The oxime **23a** (110 mg, 0.34 mmol) was dissolved in THF abs. (10 mL) and LiAlH_4_ (64.5 mg, 1.7 mmol) was added. The mixture was stirred for 19 h at rt and then heated to reflux for 4 h. After cooling to rt, ice-cooled water (10 mL) was added dropwise. When H_2_ formation was finished, the mixture was transferred into a separatory funnel, water (20 mL) and brine (10 mL) were added, and the mixture was extracted with CH_2_Cl_2_ (3 × 20 mL). The solvent was removed in vacuo, and the residue was purified by flash column chromatography (Ø 2 cm, cyclohexane:ethyl acetate = 6:4 + 1.5% *N*,*N*-dimethylethanamine, length 17 cm, fraction 10 mL, R_f_ = 0.11). Colorless oil, yield 56 mg (53%). HPLC (method ACN): purity 92%, t_R_ = 16.76 min.

(b) Reductive amination of saturated ketone **22a**

The saturated ketone **22a** (60 mg, 0.19 mmol) and NH_4_OAc (223 mg, 2.90 mmol) were dissolved in methanol abs. (12 mmol) using ultrasound. NaBH_3_CN (95%, 64 mg, 0.97 mmol) was added, and the mixture was heated to reflux for 22 h. Water (30 mL) was added, and the pH value was adjusted to 10–11 with 5 M NaOH. The aqueous layer was extracted with CH_2_Cl_2_ (3 × 25 mL). The combined organic layers were dried (K_2_CO_3_). The solvent was removed in vacuo, and the residue was purified by flash column chromatography (Ø 2 cm, cyclohexane:ethyl acetate = 7:3 + 1% *N*,*N*-dimethylethanamine, length 18 cm, fraction 10 mL, R_f_ = 0.04). Colorless oil, yield 33.5 mg (56%). HPLC (method ACN): purity 92%, t_R_ = 16.76 min. C_20_H_25_NO_2_, M_r_ = 311.4. MS (ESI): *m*/*z* [%] = 312 (M + H, 100). IR (neat): $\tilde{\nu }$ [cm^−1^] = 3032, 2926, 2851 (C-H), 1103 (C-O), 750, 697 (arom. monosubst.). ^1^H NMR (CDCl_3_): δ [ppm] = 1.25–1.84 (m, 8 H, diox-(C*H*_2_)_3_, 5-H_ax+eq_), 3.74–3.84 (m, 1 H, 4-H_ax_), 3.87–3.97 (m, 2 H, 6-H_ax_, Ph-C*H*-N), 4.25 (dd broad, J = 11.3/5.0 Hz, 1 H, 6-H_eq_), 5.47 (s, 0.5 H, 2-H_ax_), 5.48 (s, 0.5 H, 2-H_ax_), 7.21–7.39 (m, 8 H, Ar-H), 7.43–7.43 (m, 2 H, Ar-H). Signals for the N*H*_2_ protons are not seen in the spectrum. HPLC (method ACN): purity 97.2%, t_R_ = 16.76 min.

**4-(*trans*-2-Ethyl-2-phenyl-1,3-dioxan-4-yl)-1-phenylbutan-1-amine (24b).**

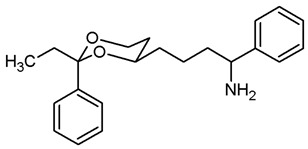


The saturated ketone **22b** (100 mg, 0.30 mmol) and NH_4_OAc (347 mg, 4.5 mmol) were dissolved in methanol abs. (20 mL) using ultrasound. NaBH_3_CN (95%, 64 mg, 0.97 mmol) was added, and the mixture was heated to reflux for 24 h, using a reflux apparatus equipped with a rubber balloon. Water (30 mL) was added, and the pH value was adjusted to 10–11 with 5 M NaOH. The aqueous layer was diluted with water and extracted with CH_2_Cl_2_ (3 × 25 mL). The combined organic layers were dried (K_2_CO_3_). The solvent was removed in vacuo, and the residue was purified by flash column chromatography (Ø 2 cm, CH_2_Cl_2_:methanol = 9.5:0.5, length 18 cm, fraction 10 mL, R_f_ = 0.07). Colorless oil, yield 46 mg (45%). C_22_H_29_NO_2_, M_r_ = 339.5. MS (EM, APCI): *m*/*z* = calculated for C_22_H_30_NO_2_ 340.2271, found 340.2302. IR (neat): $\tilde{\nu }$ [cm^−1^] = 2930, 2865 (C-H), 757, 701 (arom. monosubst.). ^1^H NMR (CDCl_3_): δ [ppm] = 0.77 (t, J = 7.5 Hz, 3 H, diox-CH_2_-C*H*_3_), 1.20–1.24 (m, 1 H, 5-H_eq_), 1.25–1.74 (m, 9 H, diox-C*H*_2_-CH_3_, diox-(C*H*_2_)_3_, 5-H_ax_), 1.95 (s broad, 2 H, N*H*_2_), 3.54–3.62 (m, 1 H, 4-H_ax_), 3.73 (t broad, J = 12.0 Hz, 1 H, 6-H_ax_), 3.84 (dd broad, J = 11.4/5.0 Hz, 1 H, 6-H_eq_), 3.93 (t broad, J = 6.9 Hz, 1 H, Ph-C*H*-N), 7.22–7.38 (m, 10 H, Ar-H). HPLC (method ACN): purity 97.3%, t_R_ = 18.03 min.

**N-Methyl-1-phenyl-4-(*cis*-2-phenyl-1,3-dioxan-4-yl)butan-1-amine (25a).**

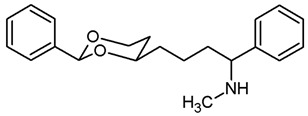


The saturated ketone **22a** (100 mg, 0.32 mmol) was dissolved in CH_2_Cl_2_ abs. (7 mL). Methylamine (2 M solution in THF, 0.24 mL, 0.48 mmol) and NaBH(OAc)_3_ (95%, 214 mg, 0.96 mmol) were added. The mixture was heated to reflux, using a reflux apparatus equipped with a rubber balloon to avoid loss of gaseous amine. Additional methylamine (2 M solution in THF, each 0.24 mL, 0.48 mmol) was added after 2 h, 3.5 h, 20 h, 24 h. Additional NaBH(OAc)_3_ (71.4 mg, 0.32 mmol) was added after 20 h. After 26 h, 1 M NaOH (15 mL) was added, and the layers were separated. The aqueous layer was extracted with CH_2_Cl_2_ (3 × 10 mL) and ethyl acetate (3 × 10 mL). The CH_2_Cl_2_ fraction and the ethyl acetate fraction were washed with brine (1 × 20 mL) separately, then combined and dried (K_2_CO_3_). The solvent was removed in vacuo, and the residue was purified by flash column chromatography (Ø 2 cm, cyclohexane:ethyl acetate = 7:3 + 3% *N*,*N*-dimethylethanamine, length 18.5 cm, fraction 10 mL, R_f_ = 0.1). Colorless oil, yield 34 mg (33%). C_21_H_27_NO_2_, M_r_ = 325.4. MS (EI): *m*/*z* [%] = 325 (M, 2), 120 (Ph-CH=NH-CH_3_, 100). IR (neat): $\tilde{\nu }$ [cm^−1^] = 2932, 2848, 2788 (C-H), 1103 (C-O), 750, 697 (arom. monosubst.). ^1^H NMR (CDCl_3_): δ [ppm] = 1.20–1.82 (m, 9 H, diox-(C*H*_2_)_3_, N*H*, 5-H_ax+eq_), 2.26 (s, 3 H, N-C*H*_3_), 3.45 (ddd, J = 8.2/6.1/2.4 Hz, 1 H, Ph-C*H*-N), 3.72–3.80 (m, 1 H, 4-H_ax_), 3.88–3.95 (m, 1H, 6-H_ax_), 4.23 (dd broad, J = 11.7/4.6 Hz, 1 H, 6-H_eq_), 5.45 (s, 0.5 H, 2-H_ax_), 5.46 (s, 0.5 H, 2-H_ax_), 7.23–7.37 (m, 8 H, Ar-H), 7.43–7.47 (m, 2 H, Ar H). HPLC (method MeOH): purity 98.1%, t_R_ = 15.73 min.

**4-(*trans*-2-Ethyl-2-phenyl-1,3-dioxan-4-yl)-*N*-methyl-1-phenylbutan-1-amine (25b).**

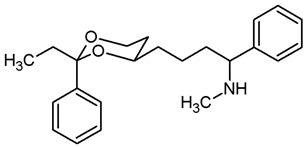


The saturated ketone **22b** (80 mg, 0.24 mmol) was dissolved in CH_2_Cl_2_ abs. (7 mL). Methylamine (2 M solution in THF, 0.36 mL, 0.72 mmol) and NaBH(OAc)_3_ (95%, 161 mg, 0.72 mmol) were added. The mixture was heated to reflux, using a reflux apparatus equipped with a rubber balloon to avoid loss of gaseous amine. Additional NaBH(OAc)_3_ (53 mg, 0.24 mmol) was added after 9 h. Additional methylamine (2 M solution in THF, each 0.24 mL, 0.48 mmol) was added after 4.5 h, 9 h, 24 h. After 28 h, 1 M NaOH (15 mL) was added. The aqueous layer was diluted with water (ca. 10 mL) and then extracted with CH_2_Cl_2_ (3 × 10 mL). The combined organic layers were washed with brine and dried (K_2_CO_3_). The solvent was removed in vacuo, and the residue was purified by flash column chromatography (Ø 2 cm, cyclohexane:ethyl acetate = 9.5:0.5 + 2% *N*,*N*-dimethylethanamine, length 17.5 cm, fraction 10 mL, R_f_ = 0.21). Colorless oil, yield 46 mg (55%). C_23_H_31_NO_2_, M_r_ = 353.5. MS (EM, APCI): *m*/*z* = calculated for C_23_H_32_NO_2_ 354.2428, found 354.2455. IR (neat): $\tilde{\nu }$ [cm^−1^] = 2932, 2865, 2788 (C-H), 757, 701 (arom. monosubst.). ^1^H NMR (CDCl_3_): δ [ppm] = 0.76 (t, J = 7.5 Hz, 3 H, diox-CH_2_-C*H*_3_), 1.18–1.78 (m, 11 H, diox-C*H*_2_-CH_3_, 5-H_ax+eq_, diox-(C*H*_2_)_3_, N-*H*), 2.28 (s, 0.5 × 3 H, N-C*H*_3_), 2.29 (0.5 × 3 H, N-C*H*_3_), 3.45–3.49 (m, 1 H, Ph-C*H*-N), 3.52–3.59 (m, 1 H, 4-H_ax_), 3.72 (t broad, J = 12.0 Hz, 1 H, 6-H_ax_), 3.83 (dd broad, J = 11.3/5.3 Hz, 1 H, 6-H_eq_), 7.22–7.37 (m, 10 H, Ar-H). HPLC (method ACN): purity 97.9%, t_R_ = 18.36 min.

***N*-Benzyl-1-phenyl-4-(*cis*-2-phenyl-1,3-dioxan-4-yl)butan-1-amine (26a).**

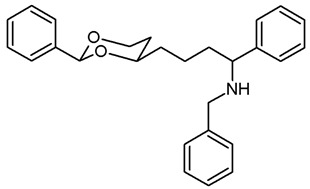


The saturated ketone **22a** (80 mg, 0.26 mmol) and freshly distilled benzylamine (42 μL, 0.39 mmol) were dissolved in CH_2_Cl_2_ abs. (5 mL). NaBH(OAc)_3_ (95%, 174 mg, 0.78 mmol) was added, and the mixture was heated to reflux. After 24 h, additional NaBH(OAc)_3_ (95%, 60 mg, 0.26 mmol) and benzylamine (42 μL, 0.39 mmol) were added, and the mixture was heated to reflux for another 8 h. Then, 1 M NaOH (15 mL) was added, and the aqueous layer was extracted with CH_2_Cl_2_ (3 × 10 mL). The combined organic layers were washed with brine and dried (K_2_CO_3_). The solvent was removed in vacuo, and the residue was purified by flash column chromatography (Ø 2.5 cm, cyclohexane:ethyl acetate = 9:1 + 0.5% *N*,*N*-dimethylethanamine, length 18 cm, fraction 10 mL, R_f_ = 0.06). Colorless oil, yield 49 mg (47%). C_27_H_31_NO_2_, M_r_ = 401.5. MS (ESI): *m*/*z* [%] = 402 (M + H, 100). IR (neat): $\tilde{\nu }$ [cm^−1^] = 3029, 2930, 2848 (C-H), 1104 (C-O), 748, 696 (arom. monosubst.). ^1^H NMR (CDCl_3_): δ [ppm] = 1.16–1.79 (m, 9 H, 5-H_ax+eq_, diox-(C*H*_2_)_3,_ N-*H*), 3.53 (d, J = 13.2 Hz, 1 H, N-C*H*_2_-Ph), 3.65 (d, J = 13.5 Hz, 1 H, N-C*H*_2_-Ph), 3.61–3.64 (m, 1 H, Ph-C*H*-N), 3.71–3.79 (m, 1 H, 4-H_ax_), 3.91 (td, J = 12.4/2.1 Hz, 1 H, 6-H_ax_), 4.23 (dd, J = 11.4/4.8 Hz, 1 H, 6-H_eq_), 5.45 (s, 0.5 H, 2-H_ax_), 5.46 (s, 0.5 H, 2-H_ax_), 7.23–7.37 (m, 13 H, Ar-H), 7.43–7.46 (m, 2 H, Ar-H). A signal for the N*H* proton is not seen in the spectrum. ^13^C NMR (CDCl_3_): δ [ppm] = 21.8 (0.5 C, diox-CH_2_-*C*H*_2_*), 21.9 (0.5 C, diox-CH_2_-*C*H*_2_*), 31.4 (0.5 C, C-5), 31.5 (0.5 C, C-5), 36.0 (0.5 C, diox-*C*H_2_), 36.1 (0.5 C, diox-*C*H_2_), 38.1 (1 C, diox-(CH_2_)_2_-*C*H_2_), 51.5 (1 C, Ph-*C*H_2_-N), 62.6 (1 C, Ph-*C*H-N-Bz), 67.2 (1 C, C-6), 77.1 (1 C, C-4), 101.2 (1 C, C-2), 126.3–128.8 (15 C, Ar), 139.0 (1 C, Ar_quart._), 140.3 (1 C, Ar_quart._), 143.8 (1 C, Ar_quart._). HPLC (method ACN): purity 97.6%, t_R_ = 20.05 min.

***N*-Benzyl-4-(*trans*-2-ethyl-2-phenyl-1,3-dioxan-4-yl)-1-phenylbutan-1-amine (26b).**

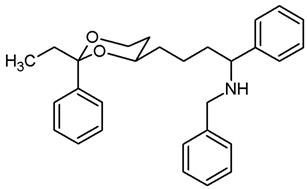


The saturated ketone **22b** (70 mg, 0.21 mmol) and freshly distilled benzylamine (69 μL, 0.63 mmol) were dissolved in CH_2_Cl_2_ abs. (5 mL). NaBH(OAc)_3_ (95%, 140 mg, 0.63 mmol) was added, and the mixture was heated to reflux for a total of 3 d. Additional NaBH(OAc)_3_ (95%, 47 mg, 0.21 mmol) was added after 24 h. Additional benzylamine (each 69 μL, 0.63 mmol) was added after 24 h and 2.5 d. After 3 d, 1 M NaOH (15 mL) was added, the organic layer was separated, and the aqueous layer was extracted with CH_2_Cl_2_ (3 × 10 mL). The combined organic layers were dried (K_2_CO_3_). The solvent was removed in vacuo, and the residue was purified by flash column chromatography (Ø 3 cm, cyclohexane:ethyl acetate = 9:1, length 16 cm, fraction 10 mL, R_f_ = 0.09).

Colorless oil, yield 75 mg (83%). C_29_H_35_NO_2_, M_r_ = 429.6. MS (ESI): *m*/*z* [%] = 430 (M + H, 100). IR (neat): $\tilde{\nu }$ [cm^−1^] = 3026, 2928, 2865 (C-H), 757, 699 (arom. monosubst.). ^1^H NMR (CDCl_3_): δ [ppm] = 0.76 (t, J = 7.5 Hz, 3 H, diox-CH_2_-C*H*_3_), 1.18 (d broad, J = 12.8 Hz, 1 H, 5-H_eq_), 1.20–1.81 (m, 7 H, diox-(C*H*_2_)_3_, 5-H_ax_), 1.70 (q, J = 7.5 Hz, 0.5 × 2 H, diox-C*H*_2_-CH_3_), 1.70 (q, J = 7.5 Hz, 0.5 × 2 H, diox-C*H*_2_-CH_3_), 3.49–3.57 (m, 1 H, 4-H_ax_), 3.54 (d, J = 13.2 Hz, 0.5 H, N-C*H*_2_-Ph), 3.54 (d, J = 13.2 Hz, 0.5 H, N-C*H*_2_-Ph), 3.64 (t, J = 7.0 Hz, 1 H, Ph-C*H*-N), 3.68 (d, J = 13.3 Hz, 1 H, N-C*H*_2_-Ph), 3.68–3.75 (m, 1 H, 6-H_ax_), 3.83 (dd broad, J = 11.3/5.0, 1 H, 6-H_eq_), 7.27–7.37 (m, 15 H, Ar-H). A signal for the N*H* proton is not seen in the spectrum. HPLC (method ACN): purity 97.4%, t_R_ = 21.11 min.

**1-[1-Phenyl-4-(*cis*-2-phenyl-1,3-dioxan-4-yl)butyl]pyrrolidine (27a).**

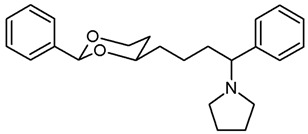


The saturated ketone **22a** (70 mg, 0.23 mmol) and freshly distilled pyrrolidine (29 μL, 0.345 mmol) were dissolved in CH_2_Cl_2_ abs. (5 mL). NaBH(OAc)_3_ (95%, 154 mg, 0.69 mmol) was added, and the mixture was heated to reflux. After 4 h, additional NaBH(OAc)_3_ (95%, 51 mg, 0.23 mmol) and pyrrolidine (29 μL, 0.345 mmol) were added, and the mixture was heated to reflux for additional 20 h. Then, 1 M NaOH (15 mL) was added, and the aqueous layer was extracted with CH_2_Cl_2_ (3 × 10 mL). The combined organic layers were washed with brine and dried (K_2_CO_3_). The solvent was removed in vacuo, and the residue was purified by flash column chromatography (Ø 2 cm, cyclohexane:ethyl acetate = 9:1 + 0.5% *N*,*N*-dimethylethanamine, length 18 cm, fraction 10 mL, R_f_ = 0.06). Colorless oil, yield 30 mg (33%). C_24_H_31_NO_2_, M_r_ = 365.5. MS (EI): *m*/*z* [%] = 365 (M, 100), 160 (Ph-CH=N(CH_2_-CH_2_)_4_, 100). IR (neat): $\tilde{\nu }$ [cm^−1^] = 2946, 2859, 2779 (C-H), 1103 (C-O), 749, 697 (arom. monosubst.) ^1^H NMR (CDCl_3_): δ [ppm] = 1.01–1.98 (m, 12 H, 5-H_ax+eq_, diox-(C*H*_2_)_3_, N(CH_2_C*H*_2_)_2_), 2.26–2.33 (m, 2 H, N(C*H*_2_CH_2_)_2_), 2.48–2.54 (m, 2 H, N(C*H*_2_CH_2_)_2_), 3.01–3.06 (m, 1 H, Ph-C*H*-N), 3.64–3.73 (m, 1 H, 4-H_ax_), 3.86 (t broad, J = 11.3 Hz, 1 H, 6-H_ax_), 5.19 (dd broad, J = 11.1/4.6 Hz, 1 H, 6-H_eq_), 5.40 (s, 0.5 H, 2-H_ax_), 5.41 (s, 0.5 H, 2-H_ax_), 7.19–7.34 (m, 8 H, Ar-H), 7.39–7.42 (m, 2 H, Ar-H). HPLC (method ACN): purity 99.6%, t_R_ = 18.83 min.

**1-[4-(*trans*-2-Ethyl-2-phenyl-1,3-dioxan-4-yl)-1-phenylbutyl]pyrrolidine (27b).**

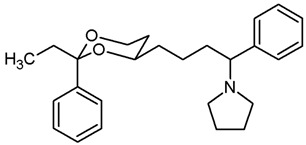


The saturated ketone **22b** (70 mg, 0.21 mmol) and freshly distilled pyrrolidine (52 μL, 0.63 mmol) were dissolved in CH_2_Cl_2_ abs. (6 mL). NaBH(OAc)_3_ (95%, 141 mg, 0.63 mmol) was added, and the mixture was heated to reflux for a total of 4 d. Additional NaBH(OAc)_3_ (95%, 47 mg, 0.21 mmol) was added after 48 h. Additional pyrrolidine (each 52 μL, 0.63 mmol) was added after 24, 48 and 72 h. After 4 d, 1 M NaOH (15 mL) was added, the organic layer was separated, and the aqueous layer was extracted with CH_2_Cl_2_ (3 × 10 mL). The combined organic layers were dried (K_2_CO_3_). The solvent was removed in vacuo, and the residue was purified by flash column chromatography (Ø 2 cm, cyclohexane:ethyl acetate = 9:1 + 0.5% *N*,*N*-dimethylethanamine, length 19 cm, fraction 10 mL, R_f_ = 0.12). Colorless oil, yield 65 mg (83%). C_26_H_35_NO_2_, M_r_ = 393.5. MS (EM, ESI): *m*/*z* = calculated for C_27_H_35_NO_2_ 394.2741, found 394.2740. IR (neat): $\tilde{\nu }$ [cm^−1^] = 2943, 2867, 2780 (C-H), 757, 702 (arom. monosubst.). ^1^H NMR (CDCl_3_): δ [ppm] = 0.74 (t, J = 7.5 Hz, 3 H, diox-CH_2_-C*H*_3_), 0.99–2.05 (m, 14 H, 5-H_ax+eq_, diox-C*H*_2_-CH_3_, diox-(C*H*_2_)_3_, N(CH_2_-C*H*_2_)_2_), 2.37, 2.48, 2.56 (s broad each, 4 H, N(C*H*_2_-CH_2_)_2_, 3.04–3.08 (m, 1 H, Ph-C*H*-N_pyrolidine_), 3.44–3.62 (m, 1 H, 4-H_ax_), 3.69 (“tt”, J = 11.4/2.5 Hz, 1 H, 6-H_ax_), 3.79–3.85 (m, 1 H, 6-H_eq_), 7.20–7.39 (m, 10 H, Ar-H). HPLC (method ACN): purity 99.9%, t_R_ = 19.98 min.

### 3.2. Receptor Binding Studies

#### 3.2.1. Materials

Guinea pig brains and rat livers were commercially available (Harlan-Winkelmann, Borchen, Germany). Homogenizers: Elvehjem Potter (B. Braun Biotech International, Melsungen, Germany) and Soniprep^®^ 150 (MSE, London, UK). Centrifuges: Cooling centrifuge model Eppendorf 5427R (Eppendorf, Hamburg, Germany) and High-speed cooling centrifuge model Sorvall^®^ RC-5C plus (Thermo Fisher Scientific, Langenselbold, Germany). Multiplates: standard 96 well multiplates (Diagonal, Muenster, Germany). Shaker: self-made device with adjustable temperature and tumbling speed (scientific workshop of the institute). Harvester: MicroBeta^®^ FilterMate 96 Harvester. Filter: Printed Filtermat Typ A and B. Scintillator: Meltilex^®^ (Typ A or B) solid state scintillator. Scintillation analyzer: MicroBeta^®^ Trilux (all Perkin Elmer LAS, Rodgau-Jügesheim, Germany).

#### 3.2.2. Preparation of Membrane Homogenates from Guinea Pig Brain

5 guinea pig brains were homogenized with the potter (500–800 rpm, 10 up and down strokes) in 6 volumes of cold 0.32 M sucrose. The suspension was centrifuged at 1200× *g* for 10 min at 4 °C. The supernatant was separated and centrifuged at 23,500× *g* for 20 min at 4 °C. The pellet was resuspended in 5–6 volumes of buffer (50 mM TRIS, pH 7.4) and centrifuged again at 23,500× *g* (20 min, 4 °C). This procedure was repeated twice. The final pellet was resuspended in 5–6 volumes of buffer and frozen (−80 °C) in 1.5 mL portions containing about 1.5 mg protein/mL.

#### 3.2.3. Preparation of Membrane Homogenates from Rat Liver

Two rat livers were cut into small pieces and homogenized with the potter (500–800 rpm, 10 up and down strokes) in 6 volumes of cold 0.32 M sucrose. The suspension was centrifuged at 1200× *g* for 10 min at 4 °C. The supernatant was separated and centrifuged at 31,000× *g* for 20 min at 4 °C. The pellet was resuspended in 5–6 volumes of buffer (50 mM TRIS, pH 8.0) and incubated at rt for 30 min. After the incubation, the suspension was centrifuged again at 31,000× *g* for 20 min at 4 °C. The final pellet was resuspended in 5–6 volumes of buffer and stored at −80 °C in 1.5 mL portions containing about 2 mg protein/mL.

#### 3.2.4. Cell Culture and Preparation of Membrane Homogenates from GluN2B Cells

Mouse L(tk-) cells stably transfected with the dexamethasone-inducible eukaryotic expression vectors pMSG GluN1a, pMSG GluN2B (1:5 ratio) were grown in Modified Earl’s Medium (MEM) containing 10% of standardized FBS Superior (Biochrom AG, Berlin, Germany). The expression of the NMDA receptor at the cell surface was induced after the cell density of the adherent growing cells had reached approximately 90% of confluency. For the induction, the original growth medium was replaced by growth medium containing 4 µM dexamethasone and 4 µM ketamine (final concentration). After 24 h, the cells were rinsed with phosphate-buffered saline solution (PBS, Biochrom AG, Berlin, Germany), harvested by mechanical detachment and pelleted (10 min, 1200× *g*).

For the binding assay, the cell pellet was resuspended in PBS solution, and the number of cells was determined using a Scepter^®^ cell counter (MERCK Millipore, Darmstadt, Germany). Subsequently, the cells were lysed by sonication (4 °C, 6 × 10 s cycles with breaks of 10 s). The resulting cell fragments were centrifuged with a high performance cool centrifuge (23,500× *g*, 4 °C). The supernatant was discarded, and the pellet was resuspended in a defined volume of PBS yielding cell fragments of approximately 500,000 cells/mL. The suspension of membrane homogenates was sonicated again (4 °C, 2 × 10 s cycles with a break of 10 s) and stored at −80 °C.

#### 3.2.5. Protein Determination

The protein concentration was determined by the method of Bradford, modified by Stoscheck. Om brief, the Bradford solution was prepared by dissolving 5 mg of Coomassie Brilliant Blue G 250 in 2.5 mL of EtOH (95%, *v*/*v*). 10 mL deionized H_2_O and 5 mL phosphoric acid (85%, *m*/*v*) were added to this solution, the mixture was stirred and filled to a total volume of 50 mL with deionized water. The calibration was carried out using bovine serum albumin as a standard in 9 concentrations (0.1, 0.2, 0.4, 0.6, 0.8, 1.0, 1.5, 2.0 and 4.0 mg/mL). In a 96 well standard multiplate, 10 µL of the calibration solution or 10 µL of the membrane receptor preparation were mixed with 190 µL of the Bradford solution, respectively. After 5 min, the UV absorption of the protein-dye complex at λ = 595 nm was measured with a plate reader (Tecan Genios^®^, Tecan, Crailsheim, Germany).

#### 3.2.6. General Procedures for the Binding Assays

The test compound solutions were prepared by dissolving approximately 10 µmol (usually 2–4 mg) of test compound in DMSO so that a 10 mM stock solution was obtained. To obtain the required test solutions for the assay, the DMSO stock solution was diluted with the respective assay buffer. The filtermats were presoaked in 0.5% aqueous polyethylenimine solution for 2 h at rt before use. All binding experiments were carried out in duplicates in the 96 well multiplates. The concentrations given are the final concentration in the assay. Generally, the assays were performed by addition of 50 µL of the respective assay buffer, 50 µL of test compound solution in various concentrations (10^−5^, 10^−6^, 10^−7^, 10^−8^, 10^−9^ and 10^−10^ mol/L), 50 µL of the corresponding radioligand solution and 50 µL of the respective receptor preparation into each well of the multiplate (total volume 200 µL). The receptor preparation was always added last. During the incubation, the multiplates were shaken at a speed of 500–600 rpm at the specified temperature. Unless otherwise noted, the assays were terminated after 120 min by rapid filtration using the harvester. During the filtration, each well was washed five times with 300 µL of water. Subsequently, the filtermats were dried at 95 °C. The solid scintillator was melted on the dried filtermats at a temperature of 95 °C for 5 min. After solidifying the scintillator at rt, the trapped radioactivity in the filtermats was measured with the scintillation analyzer. Each position on the filtermat, corresponding to one well of the multiplate, was measured for 5 min with the [^3^H]-counting protocol. The overall counting efficiency was 20%. The *IC*_50_ values were calculated with the program GraphPad Prism^®^ 3.0 (GraphPad Software, San Diego, CA, USA) by non-linear regression analysis. Subsequently, the *IC*_50_ values were transformed into *K*_i_ values using the equation of Cheng and Prusoff. The *K*_i_ values are given as mean value ± SEM from three independent experiments.

#### 3.2.7. Performance of the σ_1_ Receptor Assay [24]

The assays were performed with the radioligand [^3^H]-(+)-pentazocine (22.0 Ci/mmol; Perkin Elmer). The thawed membrane preparation of guinea pig brain (about 100 μg of the protein) was incubated with various concentrations of test compounds, 2 nM [^3^H]-(+)-pentazocine, and TRIS buffer (50 mM, pH 7.4) at 37 °C. The non-specific binding was determined with 10 μM unlabeled (+)-pentazocine. The *K*_d_ value of (+)-pentazocine is 2.9 nM.

#### 3.2.8. Performance of the σ_2_ Receptor Assay [24]

The assays were performed with the radioligand [^3^H]di-*o*-tolylguanidine (specific activity 50 Ci/mmol; ARC, St. Louis, MO, USA). The thawed rat liver membrane preparation (about 100 µg protein) was incubated with various concentrations of the test compound, 3 nM [^3^H]di-*o*-tolylguanidine and buffer containing (+)-pentazocine (500 nM (+)-pentazocine in TRIS buffer (50 mM TRIS, pH 8.0)) at rt. The non-specific binding was determined with 10 μM non-labeled di-*o*-tolylguanidine. The *K*_d_ value of di-*o*-tolylguanidine is 17.9 nM.

#### 3.2.9. Performance of the GluN2B Assay [26]

The competitive binding assay was performed with the radioligand [^3^H]ifenprodil (60 Ci/mmol; BIOTREND, Cologne, Germany). The thawed cell membrane preparation from the transfected L(tk-) cells (about 20 μg protein) was incubated with various concentrations of test compounds, 5 nM [^3^H]ifenprodil, and TRIS/EDTA-buffer (5 mM TRIS/1 mM EDTA, pH 7.5) at 37 °C. The non-specific binding was determined with 10 μM unlabeled ifenprodil. The *K*_d_ value of ifenprodil is 7.6 nM.

#### 3.2.10. Further Assays to Record Receptor Affinity

The assays to determine the affinity towards the PCP binding site of NMDA receptors [22,25], towards µ-opioid receptors [28], towards κ-opioid receptors [28] and towards δ-opioid receptors [28] were conducted as reported in literature.

### 3.3. Determination of logD_7.4_ Values [29]

Instruments and parameters for LC-MS standard analysis (in general, if not stated otherwise).

UPLC-UV/MS (Agilent, Waldbronn, Germany): degasser: 1260 HiP (G4225A); pump: 1260 Bin Pump (G1212B); autosampler: 1260 HiP ALS (G1367E); column oven: 1290 TCC (G1316C), 30 °C; UV/Vis detector: 1260 VWD (G1314F); MS-detector: 6120 Quadrupole LC/MS (G1978B). MS source: multimode source (G1978B); ESI mode; SIM mode (*m*/*z* given for each compound). Data acquisition and settings were performed with OpenLab CDS (ChemStation Edition, Agilent, Waldbronn, Germany). Guard column: Zorbax Eclipse Plus-C18 (Agilent, Waldbronn, Germany) (2.1 mm × 12.5 mm, 5.0 μm particle size). Main column: Zorbax SB-C18 (Agilent, Waldbronn, Germany) (2.1 mm × 50 mm, 1.8 μm particle size). Spray chamber: vaporizer temperature: 200 °C; drying gas: 12 L/min; nebulizer pressure: 40 psi; capillary voltage: 3000 V; corona current: 4 µA; charging voltage: 2000 V; fragmentor voltage: 100 V; drying gas temperature: 250 °C. 2 mL safe lock tubes (Eppendorf, Hamburg, Germany), 2 mL LC-MS vials (Agilent, Waldbronn, Germany).

LC-MS standard method (LC parameters in general, if not stated otherwise).

Eluents: solvent A: H_2_O/CH_3_CN 95:5 + 0.1% formic acid; solvent B: H_2_O/CH_3_CN 5:95 + 0.1% formic acid; gradient elution (A %): 0–2.5 min: gradient from 100% to 0%, 2.5–3.5 min: 0%, 3.5–4.0 min: gradient from 0% to 100%, 4.0–8.0 min: 100%. Change valve position: after 1.0 min the valve was switched from “waste” to “MS source”. Flow rate: 0.4 mL/min. Injection volume: 1.0 µL to 100 µL (given for each compound, 1.0 µL if not stated otherwise).

Chemicals, solvents and stock solutions.

3-Morpholinopropanesulfonic acid (MOPS) (Fisher Chemical, Schwerte, Germany 372.5 mg, 8.9 mM) and MOPS sodium salt (Merck KgaA, Darmstadt, Germany, 513.4 mg, 11.1 mM) were dissolved in dist. H_2_O (200 mL) to prepare a 20 mM buffer solution with pH 7.4. A mixture of n-octanol (Merck KgaA, Darmstadt, Germany) and MOPS buffer (20 mM, pH 7.4) in the ratio 1:1 was stirred overnight at room temperature (500 rpm) to saturate both liquids with each other. Afterwards, the aqueous and organic layers were separated.

10 mM stock solutions of the test compounds in DMSO (MERCK-Schuchardt, Hohenbrunn, Germany) were prepared by dissolving an exactly weighted amount of the test compound and adding the calculated amount of DMSO. Depending on the lipophilicity, either the 10 mM stock solution was used directly, or the stock solution was diluted 1:100 with MOPS buffer to a concentration of 100 µM.

General procedure.

In order to determine the logD_7.4_ value, the micro shake flask method was used [1,2]. To create physiological conditions a buffer with pH 7.4 was used to analyze the lipophilicity (logD_7.4_). The logD_7.4_ value was determined by using three different volume ratios of buffer and n-octanol (1:1, 2:1, 1:2).

Method LA (standard procedure): The 10 mM DMSO stock solution of the test compound (7.5 µL) was added to three different volumes of MOPS buffer (750 µL, 1000 µL, 500 µL) in 2 mL Eppendorf tubes. Afterwards, the tubes were filled up to 1500 µL with n-octanol (750 µL, 500 µL, 1000 µL). Each ratio was produced as a triplicate. The tubes were vortexed at rt and centrifuged at 4 °C with 16,000 rpm for 2 min.

Method LB (for very hydrophilic compounds): The 100 µM MOPS solution of the test compound (75 µL) was added to three different volumes of MOPS buffer (675 µL, 925 µL, 425 µL) in 2 mL Eppendorf tubes. n-Octanol was added to fill up the tubes to a total volume of 1500 µL (750 µL, 500 µL, 1000 µL). Each ratio was produced as a triplicate. Afterwards, the tubes were vortexed at rt and centrifuged at 4 °C with 16,000 rpm for 2 min.

An aliquot of the aqueous layer was analyzed by LC-MS standard method. For matrix-matched calibration to calculate logD_7.4_ value, the samples were diluted with MOPS buffer within a range of 1.56 nM to 1.0 µM or 39 nM to 10 µM. All samples were measured once.

### 3.4. Metabolic Stability In Vitro [29]

Preparation of mouse liver microsomes

Frozen livers (−80 °C) from male C57BL/6 mice were received from Prof. Dr. Martina Düfer from the Institute of Pharmaceutical and Medicinal Chemistry (University of Münster).

At first, the frozen livers were warmed up at 37 °C for a few min and washed with 1.15% (*m*/*v*) KCl solution at 4 °C. After cutting the livers into small pieces, the livers were homogenized in an Elvehjem-Potter (10 strokes, 3 s, 800 rpm) with cold phosphate buffer (pH 7.4, 0.1 M, 1.0 mL PBS/g liver) containing sodium EDTA (0.5 mM). PBS (pH 7.4, 0.1 M, 3.0 mL PBS/g liver), cooled on ice, was added, and the resulting suspension was centrifuged at 9000× *g* for 20 min at 4 °C. The supernatant was centrifuged again at 40,000× *g* for 90 min at 4 °C. The obtaining microsome pellet was dissolved in PBS (pH 7.4, 0.1 M). Aliquots of 1.0 mL were filled in safe lock Eppendorf tubes and stored at −80 °C.

Instruments and parameters for LC-MS standard analysis (in general, if not stated otherwise).

UPLC-UV/MS (Agilent, Waldbronn, Germany): degasser: 1260 HiP (G4225A); pump: 1260 Bin Pump (G1212B); autosampler: 1260 HiP ALS (G1367E); column oven: 1290 TCC (G1316C), 30 °C; UV/Vis detector: 1260 VWD (G1314F); MS-detector: 6120 Quadrupole LC/MS (G1978B). MS source: multimode source (G1978B); ESI mode; SIM mode (*m*/*z* given for each compound). Data acquisition and settings were performed with OpenLab CDS (ChemStation Edition, Agilent, Waldbronn, Germany). Guard column: Zorbax Eclipse Plus-C18 (Agilent, Waldbronn, Germany) (2.1 mm × 12.5 mm, 5.0 μm particle size). Main column: Zorbax SB-C18 (Agilent, Waldbronn, Germany) (2.1 mm × 50 mm, 1.8 μm particle size). Spray chamber: vaporizer temperature: 200 °C; drying gas: 12 L/min; nebulizer pressure: 40 psi; capillary voltage: 3000 V; corona current: 4 µA; charging voltage: 2000 V; fragmentor voltage: 100 V; drying gas temperature: 250 °C. 2 mL safe lock tubes (Eppendorf, Hamburg, Germany), 2 mL LC-MS vials (Agilent, Waldbronn, Germany).

LC-MS standard method (LC parameters in general, if not stated otherwise).

Eluents: solvent A: H_2_O/CH_3_CN 95:5 + 0.1 % formic acid; solvent B: H_2_O/CH_3_CN 5:95 + 0.1 % formic acid; gradient elution (A %): 0–2.5 min: gradient from 100% to 0%, 2.5–3.5 min: 0%, 3.5–4.0 min: gradient from 0% to 100%, 4.0–8.0 min: 100%. Change valve position: after 1.0 min the valve was switched from “waste” to “MS source”. Flow rate: 0.4 mL/min. Injection volume: 1.0 µL to 100 µL (given for each compound, 1.0 µL if not stated otherwise).

Chemicals, solvents and stock solutions.

NADPH Na_4_ (Carl Roth, Karlsruhe, Germany) and UDPGA Na_3_ (Merck KgaA, Darmstadt, Germany) were dissolved in phosphate buffer (PBS, 0.1 M, pH 7.4, Merck KGaA, Darmstadt, Germany) to prepare a 2.0 mg/mL solution, respectively. MgCl_2_ (Honeywell Specialty Chemicals, Seelze, Germany) was dissolved in bidist. H_2_O to a 0.05 M solution. 1.0 mM solutions of the test compounds were prepared from the 10 mM DMSO stock solutions by diluting 1:10 with DMSO (MERCK-Schuchardt, Hohenbrunn, Germany).

Phase I Metabolism.

NADPH-Na_4_ (2.0 mg/mL in 0.1 M PBS, 50 µL), MgCl_2_ (0.05 M in H_2_O, 50 µL) and phosphate buffer (PBS, 0.1 M, 76.8 µL) were mixed in an Eppendorf tube. The test compound (1.0 mM in DMSO, 1.2 µL) and mouse liver microsomes (MLM, 22 µL) were added. Instead of test compound, imipramine (1.0 mM in DMSO, 1.2 µL) was incubated with mouse liver microsomes as positive control. The metabolic stability of imipramine using this procedure is well known (20% of parent compound after 90 min incubation). The prepared samples were incubated at 37 °C for 90 min at 900 rpm at the thermomixer (Eppendorf). The incubation was stopped by adding CH_3_CN/CH_3_OH 1:1 (400 µL) to the samples and ice-cooling for 10 min to precipitate the proteins. The samples were centrifuged at 4 °C for 15 min at 16,000 rpm. An aliquot of the supernatant was measured by LC-MS standard method. An “empty sample” (without test compound, PBS was added to replace the missing volume) was prepared in the same way. Additionally, “blanks” (without NADPH Na_4_, PBS was added to replace the missing volume) were prepared according to the same procedure. The test compound (10 mM in DMSO, 1.2 µL) was added after precipitating the proteins under ice-cooling for 10 min.

## 4. Conclusions

Homologation of the primary ethanamine **2a** (*K*_i_(PCP) = 19 nM) to primary butanamine **14b** (*K*_i_(PCP) = 731 nM) led to considerably reduced affinity towards the PCP binding site of the NMDA receptor. An additional phenyl moiety in α-position of the primary amine slightly increased the PCP affinity of **24b** (*K*_i_(PCP) = 524 nM). It was concluded that the PCP binding site did not tolerate homologation of ethanamine **2a** by two CH_2_ moieties.

However, homologation of the benzylated ethanamine **1** (*K*_i_(σ_1_) = 19 nM) by two CH_2_ moieties led to the benzylated butanamine **17a** *K*_i_(σ_1_) = 31 nM) exhibiting almost the same σ_1_ affinity as **1**. Methylation of the secondary amine **17a** resulted in the very potent σ_1_ ligand **18a** (*K*_i_(σ_1_) = 6.3 nM). 1,3-Dioxanes **17b** *K*_i_(σ_1_) = 14 nM) and **18b** *K*_i_(σ_1_) = 8.7 nM) derived from propiophenone showed almost the same σ_1_ affinity as the analogous benzaldehyde derivatives **17a** and **18a**. Obviously, the σ_1_ receptor well tolerates homologation of the aminoethyl side chain and the change in relative configuration of 1,3-dioxane-based ligands.

The benzaldehyde-derived 1,3-dioxanes **17a** and **18a** show higher selectivity for σ_1_ receptors over σ_2_ receptors and the ifenprodil binding site of the NMDA receptor than the analogous propiophenone derivatives **17b** and **18b**. The 1,3-dioxanes **17a**,**b** and **18a**,**b** neither bound at the PCP binding site of the NMDA receptor nor at opioid receptors. These data indicate very high selectivity for the σ_1_ receptor.

The benzyl group at the amino moiety of **17a**,**b** and **18a**,**b** corresponds to the second hydrophobic region postulated in σ_1_ pharmacophore models. Compounds **24**–**27** did not reach the σ_1_ affinity of the benzylamines **17a**,**b** and **18a**,**b**, although they contain an additional phenyl moiety at the butyl side chain as second hydrophobic region. However, small aliphatic substituents at the amino moiety of these ligands (e.g., propiophenone derivatives **25b**, **27b**) already led to moderate σ_1_ affinity.

The highest LLE values were determined for the benzylamine **17a** (LLE = 6.19) and the pyrrolidine **19a** (LLE = 6.72). Due to their excellent selectivity over σ_2_ receptors, the PCP and ifenprodil binding sites at NMDA receptors, and µ-, κ-, and δ-opioid receptors (only shown for **17a**), **17a** and **19a** represent the most promising σ_1_ ligands of this series of compounds, although they exhibit only moderate σ_1_ affinity (*K*_i_(**17a**) = 31 nM, *K*_i_(**19a**) = 154 nM). Both ligands showed a medium metabolic stability, i.e., after incubation with mouse liver microsomes and NADPH for 90 min, 56% and 55% of intact parent compound **17a** and **19a** were detected, respectively.

## Figures and Tables

**Table 1:** Affinities of butan-1-amines **14**, **16**–**19** and **24**–**27** towards σ_1_, and σ_2_ receptors as well as towards the PCP and ifenprodil binding sites of the NMDA receptor. Affinity data of reference compounds for each receptor subtype are included.

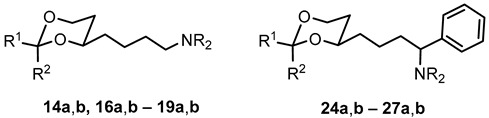							
Compd.	R^1^	R^2^	NR_2_	*K*_i_ ± SEM [nM] (n = 3) ^(a)^			
				σ_1_	σ_2_	PCP	GluN2B
**14a**	Ph	H	NH_2_	>1,000	>1000	>1000	>1000
**14b**	Et	Ph		>1,000	>1000	731	>1000
**16a**	Ph	H	NHCH_3_	842	>1000	>1000	>1000
**16b**	Et	Ph		107 ± 5	311	>1000	>1000
**17a**	Ph	H	NHBn	31 ± 4.0	213	>1000	>1000
**17b**	Et	Ph		14 ± 6.1	22 ± 1.9	569 ± 141	39 ± 13
**18a**	Ph	H	N(CH_3_)Bn	6.3 ± 2.4	31 ± 3.1	>1000	261 ± 54
**18b**	Et	Ph		8.7 ± 3.6	24 ± 6.3	>1000	20 ± 3.3
**19a**	Ph	H	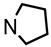	154 ± 8	>1000	>1000	>1000
**19b**	Et	Ph		56 ± 19	57 ± 1.9	>1000	67 ± 18
**24a**	Ph	H	NH_2_	>1000	1000	>1000	1350
**24b**	Et	Ph		427	170	524	136 ± 36
**25a**	Ph	H	NHCH_3_	144 ± 26	>1000	>1000	716
**25b**	Et	Ph		58 ± 8.2	255	>1000	190 ± 39
**26a**	Ph	H	NHBn	918	907	>1000	>1000
**26b**	Et	Ph		88 ± 43	139	>1000	257 ± 57
**27a**	Ph	H	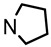	161 ± 59	>1000	>1000	366
**27b**	Et	Ph		19 ± 0.6	64 ± 15	>1000	63 ± 20
(+)-pentazocine				5.7 ± 2.2	-		
Haloperidol				6.3 ± 1.6	78 ± 2.3		
di-*o*-tolylguanidine				89 ± 29	58 ± 18		
Memantine				-	-	740 ± 47	
Dexoxadrol				-	-	25 ± 4	
Ifenprodil				-	-	-	10 ± 0.7
Traxoprodil				-	-	-	128 ± 42

^(a)^ The given K_i_ values represent means of three independent experiments (n = 3). Values in % represent the inhibition of the radioligand binding at a test compound concentration of 1 µM. Values without SEM represent the mean of two experiments.

**Table 2:** Lipophilicity and metabolic stability of promising σ_1_ receptor ligands.

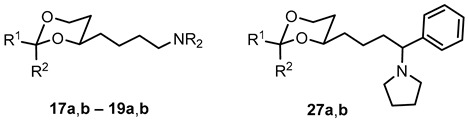							
Compd.	R^1^	R^2^	NR_2_	p*K*_i_(σ_1_)	logD_7.4_	LLE ^(a)^	Metabolic Stability (5) ^(b)^
**17a**	Ph	H	NHBn	7.51	1.32 ± 0.04	6.19	56 ± 3
**17b**	Et	Ph		7.85	2.65 ± 0.04	5.20	50 ± 4
**18a**	Ph	H	N(CH_3_)Bn	8.20	2.49 ± 0.05	5.71	48 ± 4
**18b**	Et	Ph		8.06	3.54 ± 0.06	4.52	36 ± 1
**19a**	Ph	H	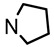	6.81	0.09 ± 0.05	6.72	55 ± 2
**19b**	Et	Ph		7.25	1.29 ± 0.05	5.96	62 ± 2
**27a**	Ph	H	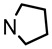	6.79	2.20 ± 0.07	4.59	76 ± 3
**27b**	Et	Ph		7.72	3.33 ± 0.08	4.39	55 ± 5
imipramine							29 ± 3

^(a)^ LLE (ligand-lipophilicity efficiency) = p*K*_i_(σ_1_) − logD_7.4_. ^(b)^ Amount of the remaining unmetabolized parent compound (in %) after 90 min incubation with mouse liver microsomes and NADPH.

## Data Availability

Data is contained within the article or supplementary material

## Supplementary Materials

The following supporting information can be downloaded at: https://www.mdpi.com/1424-8247/18/9/1300/s1. The Supporting Information contains the opioid receptor affinity data of butanamines 17 and 18, general chemistry methods, HPLC methods to determine the purity of compounds, synthesis of pentane-1,3,5-triol, comparison of the structures of etoxadrol, ethanamine 2a and butanamine 14b, as well as NMR spectra, mass spectra and HPLC traces of all test compounds to prove their purity.

## Abbreviations

DIAD, diisopropyl azodicarboxylate; DMSO, dimethyl sulfoxide; LDA, lithium diisopropylamide; LLE, ligand-lipophilicity efficiency; NADPH, nicotinamide adenine dinucleotide phosphate; NMDA, *N*-methyl-d-aspartate; PCP, 1-(1-phenylcyclohexyl)piperidine (phencyclidine); PET, positron emission tomography; SEM, standard error of the mean; THF, tetrahydrofuran.
