# Supplementary material for: Synthesis and Structure-Affinity Relationships of Receptor Ligands with 1,3-Dioxane Structure
Source: Pharmaceuticals (Basel). 2025 Aug 29;18(9):1300. doi: 10.3390/ph18091300 (PMC12865480; doi:10.3390/ph18091300)
Supplement: Supplementary file 1 [file pharmaceuticals-18-01300-s001.zip › pharmaceuticals-3815077-supplementary.pdf]

Supporting information

Synthesis and Structure-Affinity Relationships of Receptor Ligands with 1,3-Dioxane Structure

Elisabeth Quick, Dirk Schepmann, Bernhard Wünsch *

Institut für Pharmazeutische und Medizinische Chemie, Universität Münster, Correnssstraße 48, D-48149 Münster, Germany

* Correspondence: wuensch@uni-muenster.de; Tel.: +49-251-8333311

Content page

1. Opioid receptor affinity of the most potent σ_1 receptor ligands S2
2. Chemistry, general methods S3
3. HPLC methods for determination of purity S4
4. Synthesis of pentane-1,3,5-triol (**6**) S5
5. Comparison of etoxadrol (**28**) with ethanamine **2** and butanamine **14b** S6
6. Spectroscopic and chromatographic data of test compounds S7

1. Opioid receptor affinity of most potent σ1 receptor ligands

Table S1: Opioid receptor affinity of the most potent σ_1 receptor ligands. Affinity data of reference compounds for each receptor subtype are included.

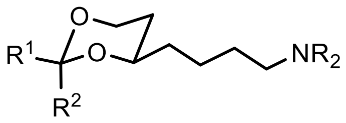

17a,b, 18a,b

compd.	R^1	R^2	NR_2	K_i ± SEM [nM] (n = 3) [a)]			
				σ_1 receptor	μ receptor	κ receptor	δ receptor
**17a**	Ph	H	NHBn	31 ± 4.0	3 %	0 %	282
**17b**	Et	Ph		14 ± 6.1	293	0 %	612
**18a**	Ph	H	$N(CH_3)Bn$	6.3 ± 2.4	0 %	0 %	0 %
**18b**	Et	Ph		8.7 ± 3.6	0 %	0 %	0 %
morphine					5.2 ± 1.6	35 ± 6	-
naloxone					2.3 ± 1.1	6.9 ± 0.50	103
U-50,488					-	0.34 ± 0.07	-
SNC80					-	-	1.2 ± 0.5

a) The given K_i values represent means of three independent experiments (n = 3). Values in % represent the inhibition of the radioligand binding at a test compound concentration of 1 µM. Values without SEM represent the mean of two experiments.

2. Chemistry, General Methods

Oxygen and moisture sensitive reactions were carried out under nitrogen, dried with silica gel with moisture indicator (orange gel, VWR, Darmstadt, Germany) and in dry glassware (Schlenk flask or Schlenk tube). Temperature was controlled with dry ice/acetone (-78 °C), ice/water (0 °C), Cryostat (Julabo TC100E-F, Seelbach, Germany), magnetic stirrer MR 3001 K (Heidolph, Schwalbach, Germany) or RCT CL (IKA, Staufen, Germany), together with temperature controller EKT HeiCon (Heidolph) or VT-5 (VWR) and PEG or silicone bath. All solvents were of analytical or technical grade quality. Demineralized water was used. CH_2Cl_2 was distilled from CaH_2; THF was distilled from sodium/benzophenone; MeOH was distilled from magnesium methanolate. Thin layer chromatography (tlc): tlc silica gel 60 F_{254} on aluminum sheets (VWR). Flash chromatography (fc): Silica gel 60, 40–63 µm (VWR); parentheses include: diameter of the column (Ø), length of the stationary phase (h), fraction size (v) and eluent. Automated flash chromatography: Isolera™ Spektra One (Biotage®); parentheses include: cartridge size, eluent, fraction size was always 20 mL. Melting point: Melting point system MP50 (Mettler Toledo, Gießen, Germany), open capillary, uncorrected. MS: MicroTOFQII mass spectrometer (Bruker Daltonics, Bremen, Germany); deviations of the found exact masses from the calculated exact masses were 5 ppm or less; the data were analyzed with DataAnalysis® (Bruker Daltonics). NMR: NMR spectra were recorded in deuterated solvents on Agilent DD2 400 MHz and 600 MHz spectrometers (Agilent, Santa Clara CA, USA); chemical shifts (*δ*) are reported in parts per million (ppm) against the reference substance tetramethylsilane and calculated using the solvent residual peak of the undeuterated solvent; coupling constants are given with 0.5 Hz resolution; assignment of ^{1}H and ^{13}C NMR signals was supported by 2-D NMR techniques where necessary. IR: FT/IR IR Affinity®-1 spectrometer (Shimadzu, Düsseldorf, Germany) using ATR technique.

3. HPLC methods for determination of purity

Pump: L-7100, degasser: L-7614, autosampler: L-7200, UV detector: L-7400, interface: D-7000, data transfer: D-line, data acquisition: HSM-Software (all from Merck Hitachi, Darmstadt, Germany); column: LiChrospher® 60 RP-select B (5 µm), LiChroCART® 250-4 mm cartridge; flow rate: 1.0 mL/min; injection volume: 5.0 µL; detection at λ = 210 nm.

Method ACN

Solvents: A: demineralized water with 0.05 % (V/V) trifluoroacetic acid, B: CH_3CN with 0.05 % (V/V) trifluoroacetic acid; gradient elution (% A): 0 – 4 min: 90 %; 4 – 29 min: gradient from 90 % to 0 %; 29 – 31 min: 0 %; 31 – 31.5 min: gradient from 0 % to 90 %; 31.5 – 40 min: 90 %. Unless otherwise mentioned, the purity of all test compounds is greater than 95 %.

Method MeOH

Solvents: A: demineralized water with 0.05 % (V/V) trifluoroacetic acid, B: CH_3OH with 0.05 % (V/V) trifluoroacetic acid; gradient elution (% A): 0 – 1 min: 80 %; 1 – 22 min: gradient from 80 % to 0 %; 22 – 30 min: 0 %; 30 – 31 min: gradient from 0 % to 80 %; 31 – 40 min: 80 %. Unless otherwise mentioned, the purity of all test compounds is greater than 95 %.

4. Synthesis of pentane-1,3,5-triol (6)

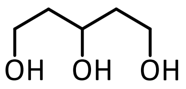

Under ice cooling and N_2 atmosphere, a solution of diethyl 3-hydroxyglutarate (12.44 g, 61 mmol) in THF (95 mL) was added dropwise to a suspension of $LiAlH_4$ (5.0 g, 130 mmol) in THF (95 mL) and the reaction mixture was heated to reflux for 48 h. The remaining excess of $LiAlH_4$ was destroyed with water (95 mL) at rt, H_2SO_4 (¼ conc.) was added until the precipitate was dissolved completely (pH 1) and the mixture was heated to reflux for further 17 h. THF was evaporated in vacuo, water (200 mL) was added and the mixture was alkalized (pH 10) with NH_3 (25 %). The resulting precipitate of $Al(OH)_3$ was removed by suction through a filter paper with a sufficient amount of sea sand on top and washed with water. The filtrate was evaporated in vacuo and the dry residue was extracted with ethanol (100 mL) under reflux for 17 h. Insoluble Li_2SO_4 was filtered off at 50 °C and washed with ethanol (100 mL). The ethanol was removed in vacuo and the residue was purified by flash column chromatography (Ø 8 cm, ethyl acetate : methanol = 9 : 1, length 18 cm, fraction 65 mL, R_f = 0.15) followed by distillation in high vacuum, bp 127 °C (1.6 x 10-2 mbar). Colorless oil, yield 5.92 g (81 %). $C_5H_{12}O_3$, M_r = 120.1. MS (EI): m/z [%] = 121 (M + H, 100). IR (neat): $\tilde{\nu}$ [cm^{-1}] = 3294 (O-H), 2938, 2883 (C-H), 1041 (C-O). ^1H NMR (CD_3OD): δ [ppm] = 1.60 – 1.73 (m, 4 H, CH_2-CH-CH_2), 3.69 (t, J = 6.5 Hz, 4 H, CH_2-OH), 3.87 (tt, J = 8.1 / 4.5 Hz, 1 H, CH_2-C*H*-CH_2). Signals for the O*H* protons are not seen in the spectrum.

5. Comparison of etoxadrol (28) with ethanamine 2a and butanamine 14b

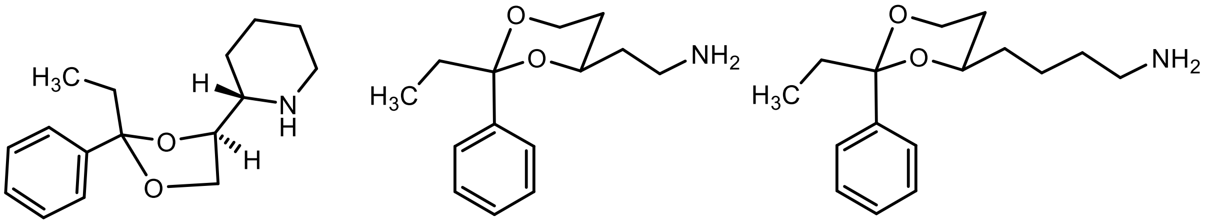

28 (etaoxadrol) **2a** **14b**

Figure S1: Etoxadrol (**28**) compared with 1,3-dioxane-based ethanamine **2a** and butanamine **14b** interacting with high (**28**, **2a**) and moderate (**14b**) affinity with the PCP binding site of the NMDA receptor.

6. Spectroscopic and chromatographic data of test compounds

Spectroscopic and chromatographic data of compound **14a**

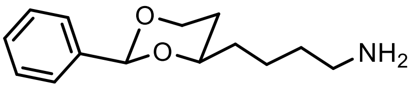

14a

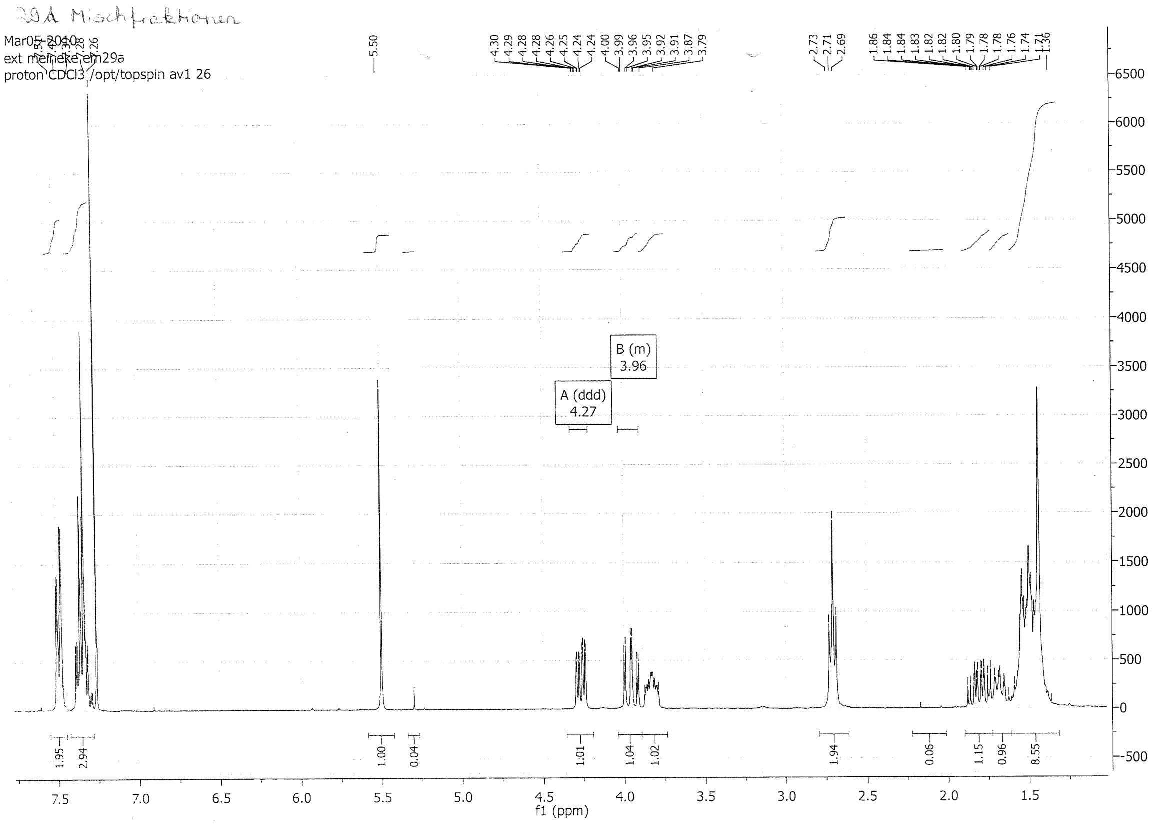

MicroTof (Bruker Daltonics) OC-Münster Abt. Massenspektrometrie

Analysis Info

Analysis Name	Z:\Wuensch EM 29-a_acn_Tray1-72_01_113294.d	Acquisition Date	06.05.2010 13:43:16
Sample Name	Wuensch EM 29-a_acn	Method	oa_ms_pos_lm_ot.m
Comment	MeOH pur		

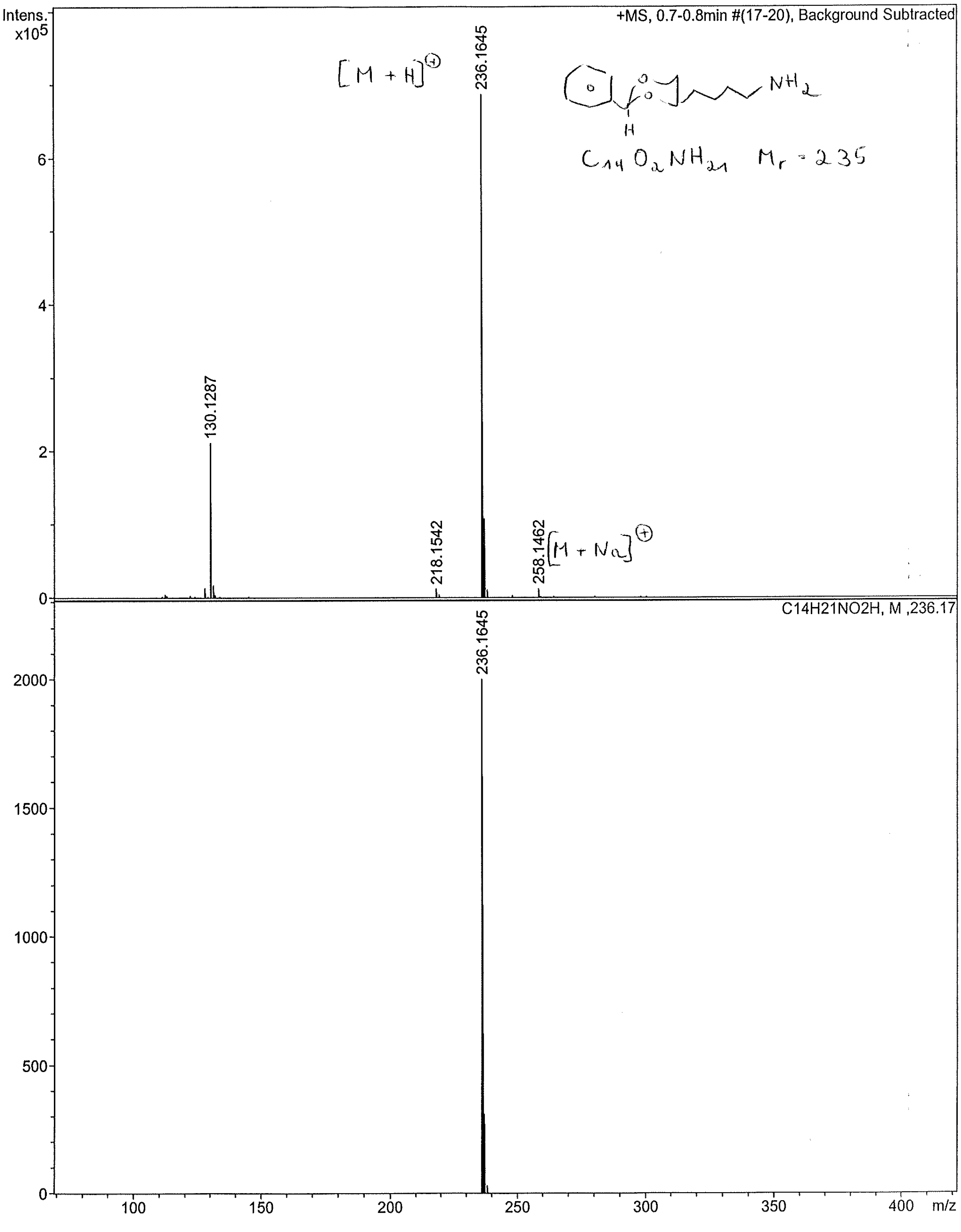

HPLC

Analyzed: 11.03.10 04:17

Reported: 12.03.10 09:40
Processed: 12.03.10 09:40

Data Path: D:\WIN32APP\HSM\Chromni\DATA\1291\
Application: Chromni
Sample Name: EM29A
Injection from this vial: 1 of 1

Series:1291
Vial Number: 11
Vial Type: UNK
Volume: 5,0 ul

Chrom Type: HPLC Channel : 1

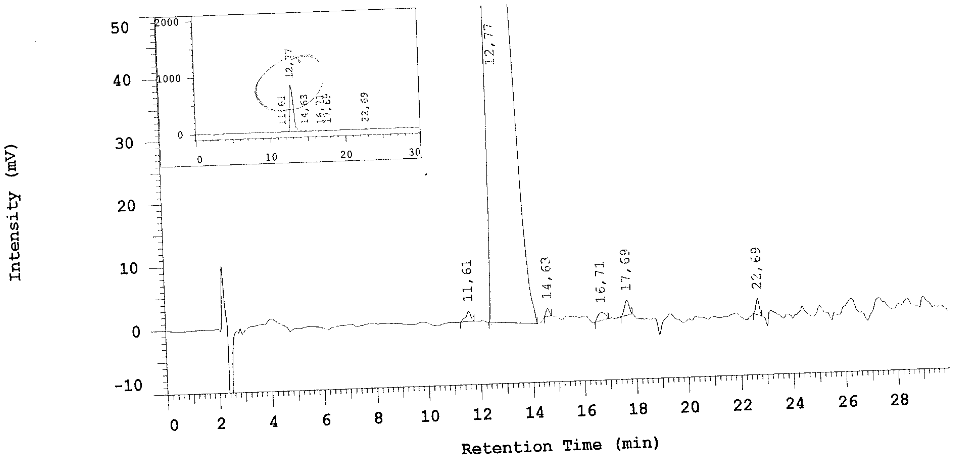

Acquisition Method: Chromni
Blank Subtr Sample Name: ACN
Column Type: 010
Solvent A: Wasser + 0,05%TFA

Developed by: Jens
Solvent B: ACN + 0,05%TFA

No.	RT	Area	Conc 1	BC
1	11,61	21606	0,081	MC
2	12,77	26526257	99,591	MC
3	14,63	11010	0,041	MC
4	16,71	25085	0,094	MC
5	17,69	29002	0,109	MC
6	22,69	22262	0,084	MC
		26635222	100,000	

Peak rejection level: 0

Spectroscopic and chromatographic data of compound **14b**

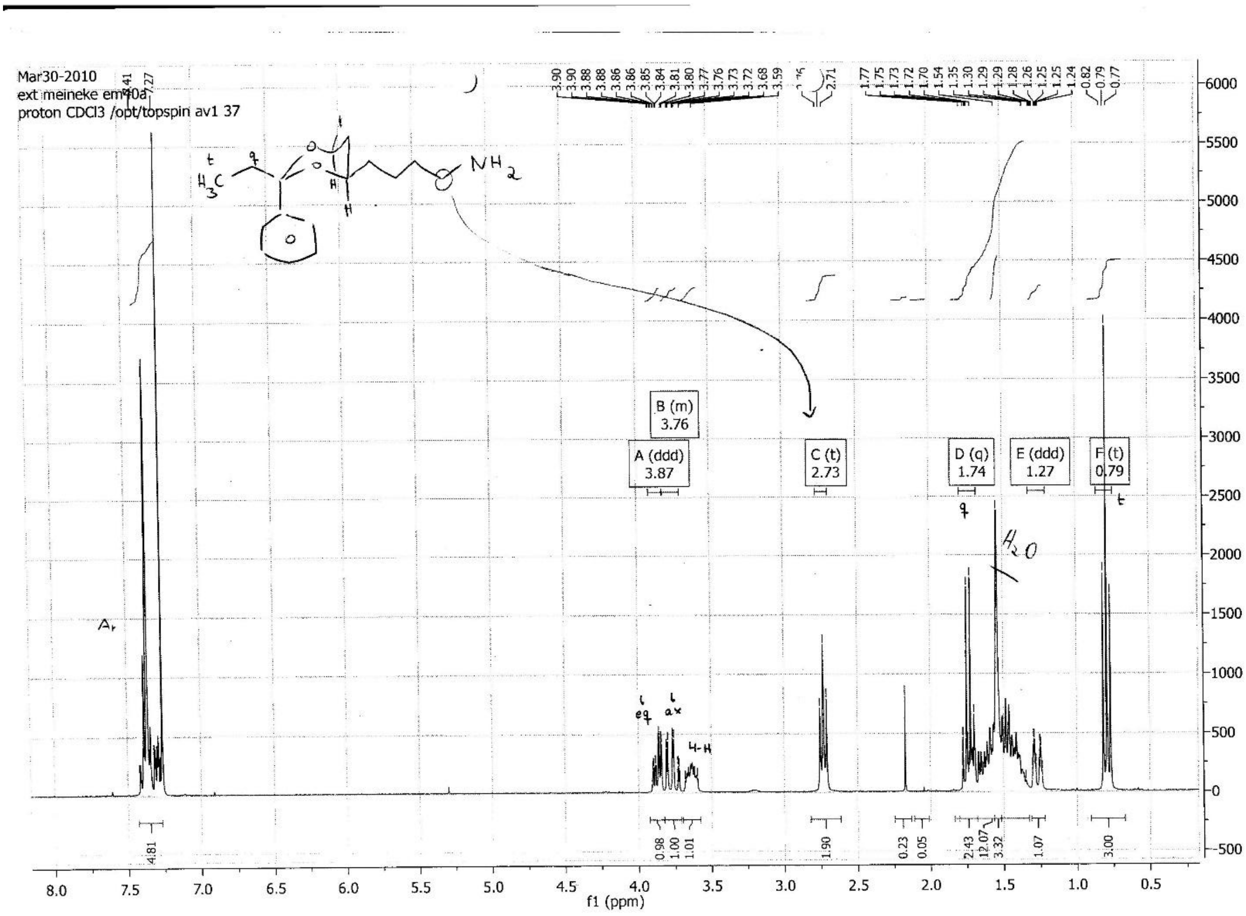

MicroTof (Bruker Daltonics) OC-Münster Abt. Massenspektrometrie

Analysis Info

Analysis Name	Z:\Wuensch EM 40-a_acn_Tray1-74_01_113296.d	Acquisition Date	06.05.2010 13:50:38
Sample Name	Wuensch EM 40-a_acn	Method	oa_ms_pos_lm_ot.m
Comment	MeOH pur		

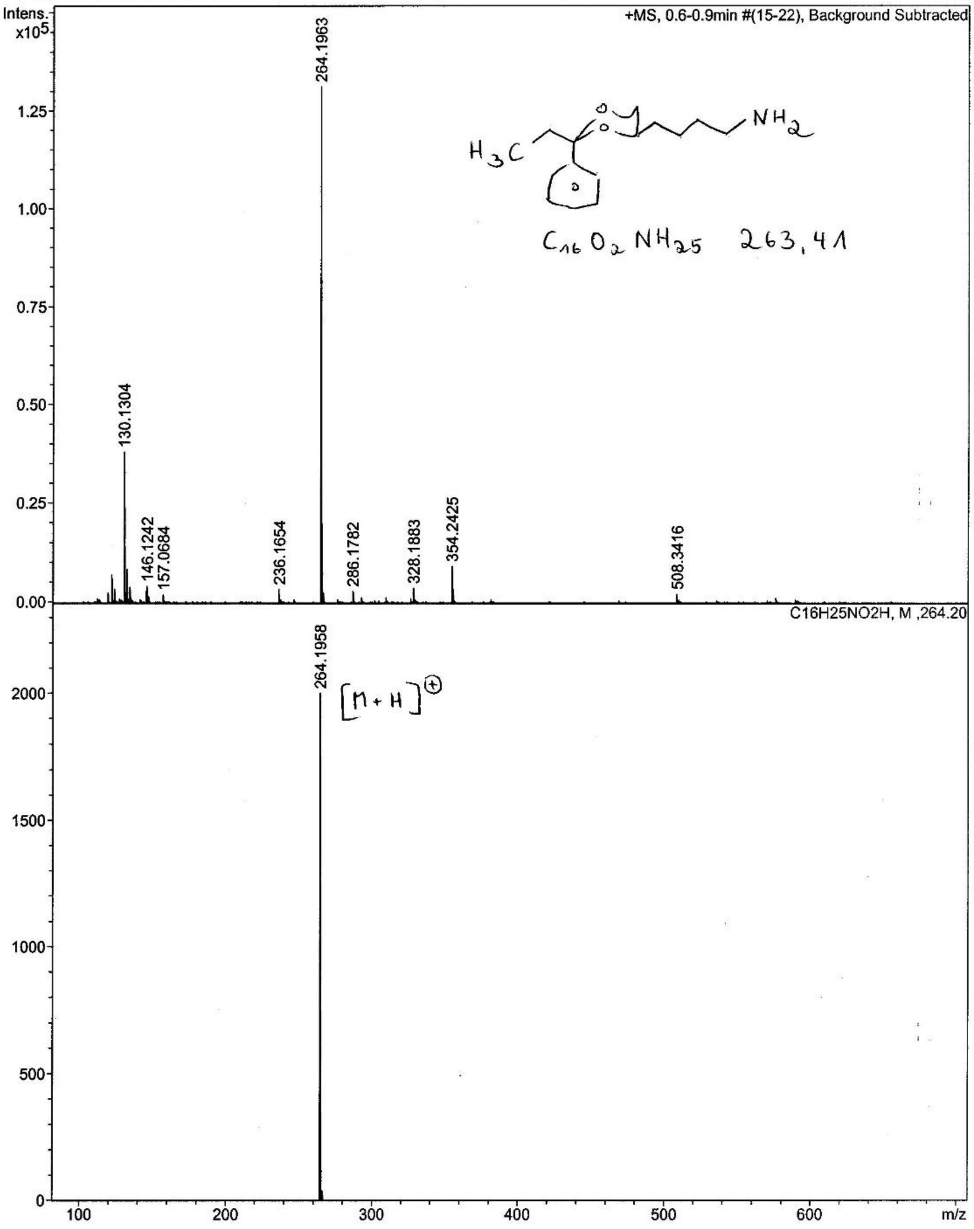

Bruker Compass DataAnalysis 4.0 printed: 07.05.2010 09:07:39 Page 1 of 1

HPLC

Analyzed: 18.03.10 21:47

Reported: 19.03.10 13:16
Processed: 19.03.10 13:16

Data Path: D:\WIN32APP\HSM\Chromni\DATA\1319\
Application: Chromni
Sample Name: EM40A
Injection from this vial: 1 of 1

Series:1319
Vial Number: 5
Vial Type: UNK
Volume: 5,0 ul

Chrom Type: HPLC Channel : 1

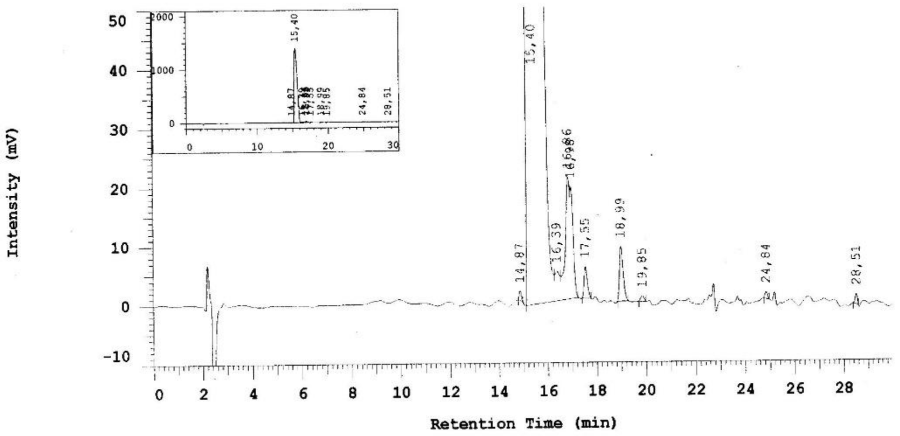

Acquisition Method: Chromni
Blank Subtr Sample Name: ACN
Column Type: 010
Solvent A: Wasser + 0,05%TFA

Developed by: Jens
Solvent B: ACN + 0,05%TFA

No.	RT	Area	Conc 1	BC
1	14,87	10502	0,027	MC
2	15,40	38525974	98,270	MC
3	16,39	73746	0,188	MC
4	16,86	257496	0,657	MC
5	16,98	168297	0,429	MC
6	17,55	48022	0,122	MC
7	18,99	94022	0,240	MC
8	19,85	8457	0,022	MC
9	24,84	7590	0,019	MC
10	28,51	10160	0,026	MC
		39204266	100,000	

Peak rejection level: 0

Spectroscopic and chromatographic data of compound **16a**

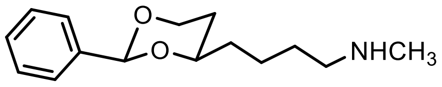

16a

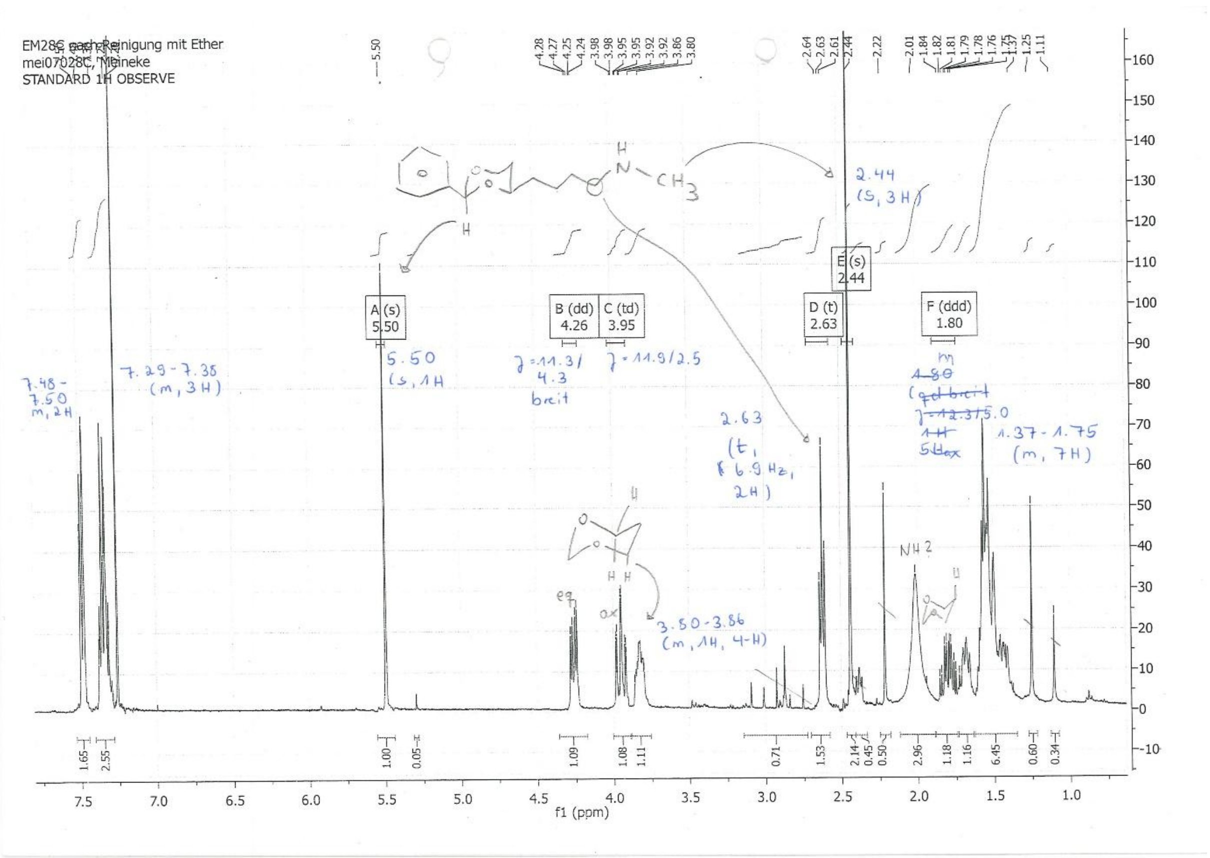

Generic Display Report

**Analysis Info**		Acquisition Date	8/16/2010 2:18:37 PM
Analysis Name	D:\Data\IPMC\PharmChemie\Routine\2010_08_16\WME_EM28C_GB2_01_991.d		
Method	kurz_pos_ms_low.m	Operator	Meiners
Sample Name	WME_EM28C	Instrument	micrOTOF-Q II
Comment	Meineke<br>EM28C		

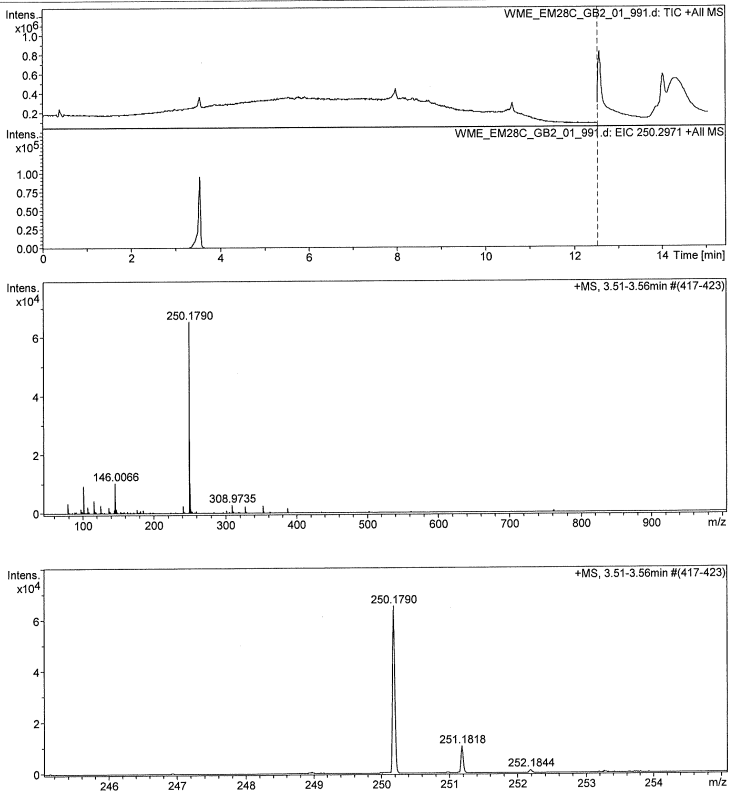

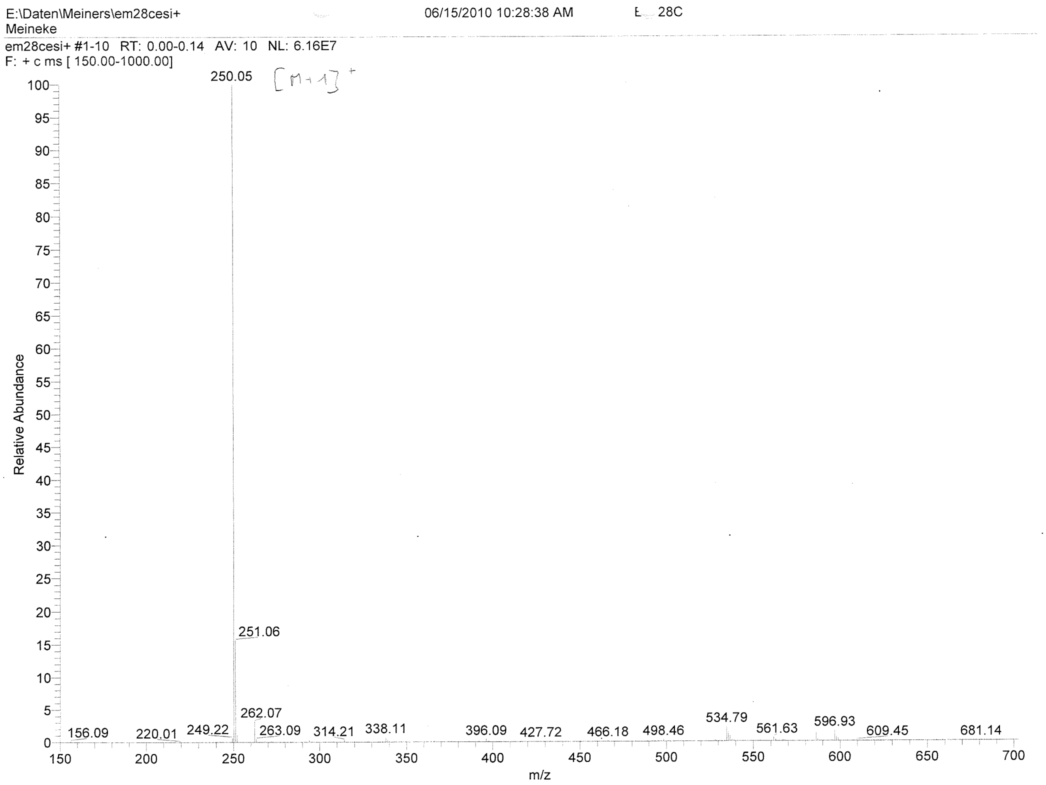
E:\Daten\Meiners\em28cesi+
Meineke
06/15/2010 10:28:38 AM
28C
em28cesi+ #1-10 RT: 0.00-0.14 AV: 10 NL: 6.16E7
F: + c ms [150.00-1000.00]
250.05
[M+1]+
251.06
262.07
156.09
220.01
249.22
263.09
314.21
338.11
396.09
427.72
466.18
498.46
534.79
561.63
596.93
609.45
681.14
Relative Abundance
m/z

HPLC

Analyzed: 19.08.10 18:40

Reported: 19.08.10 20:08
Processed: 19.08.10 20:08

Data Path: D:\WIN32APP\HSM\Chromni\DATA\1987\
Application: Chromni
Sample Name: EM28D
Injection from this vial: 1 of 1

Series:1987
Vial Number: 31
Vial Type: UNK
Volume: 5,0 ul

Chrom Type: HPLC Channel : 1

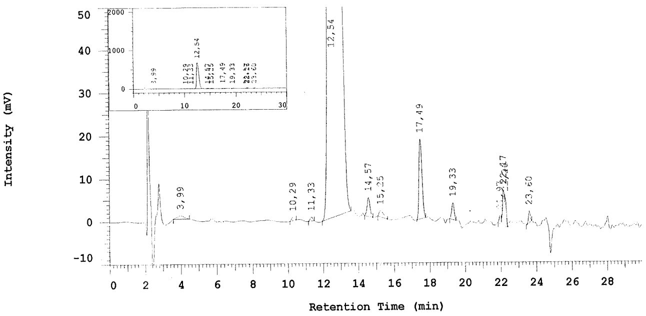

Acquisition Method: Chromni
Blank Subtr Sample Name: ACN
Column Type: 010
Developed by: Jens
Solvent A: Wasser + 0,05%TFA
Solvent B: ACN + 0,05%TFA

No.	RT	Area	Conc 1	BC
1	3,99	22994	0,111	MC
2	10,29	7849	0,038	MC
3	11,33	8760	0,042	MC
4	12,54	20261806	97,543	MC
5	14,57	49016	0,236	MC
6	15,25	26076	0,126	MC
7	17,49	225025	1,083	MC
8	19,33	39647	0,191	MC
9	21,97	14095	0,068	MC
10	22,17	64637	0,311	MC
11	22,26	34686	0,167	MC
12	23,60	17650	0,085	BB
		20772241	100,000	

Peak rejection level: 0

Spectroscopic and chromatographic data of compound **16b**

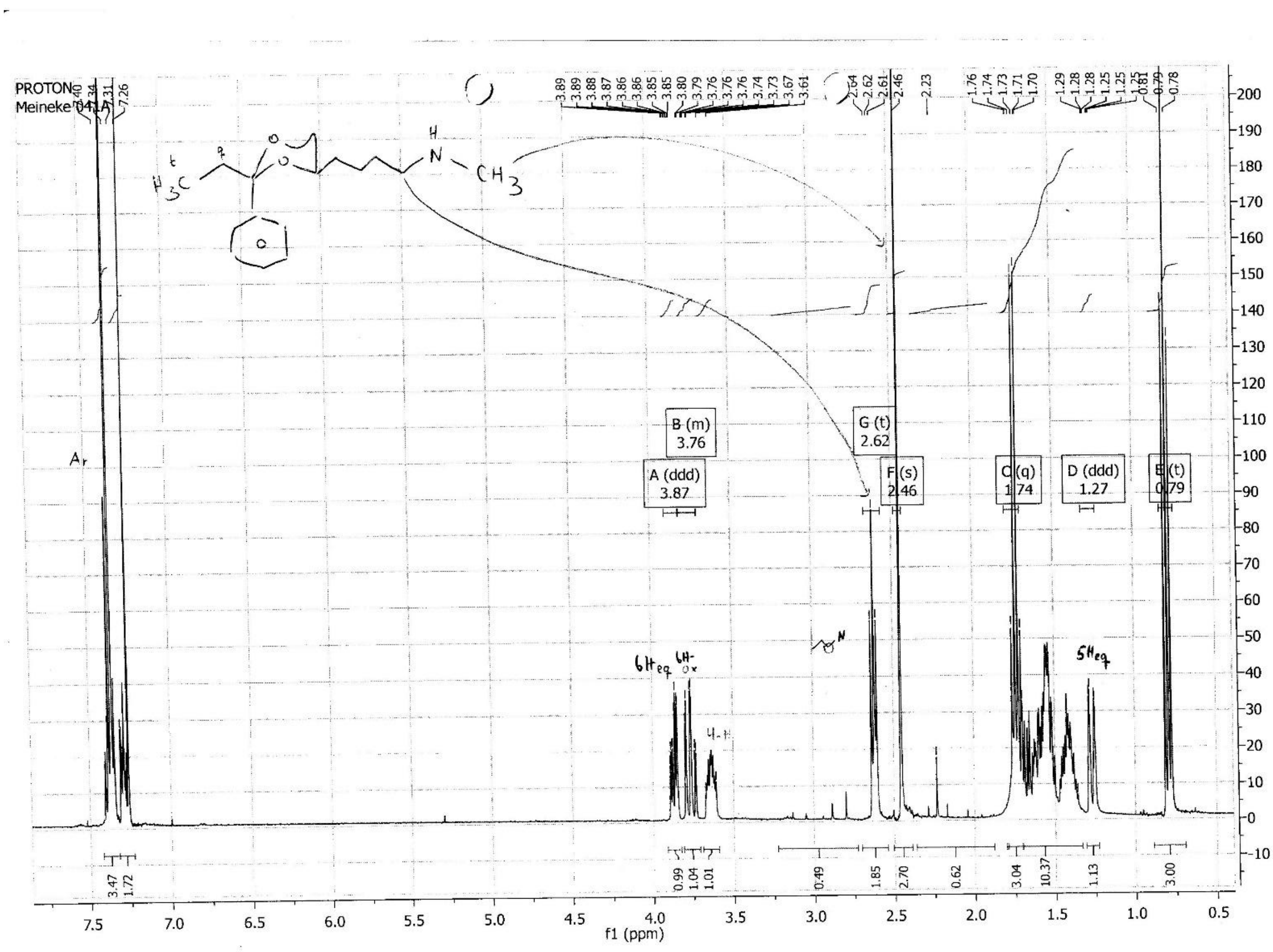

MicroTof (Bruker Daltonics) OC-Münster Abt. Massenspektrometrie

Analysis Info

Analysis Name	Z:\Wuensch EM 41-a_acn_Tray1-75_01_113297.d	Acquisition Date	06.05.2010 13:54:18
Sample Name	Wuensch EM 41-a_acn	Method	oa_ms_pos_lm_ot.m
Comment	MeOH pur		

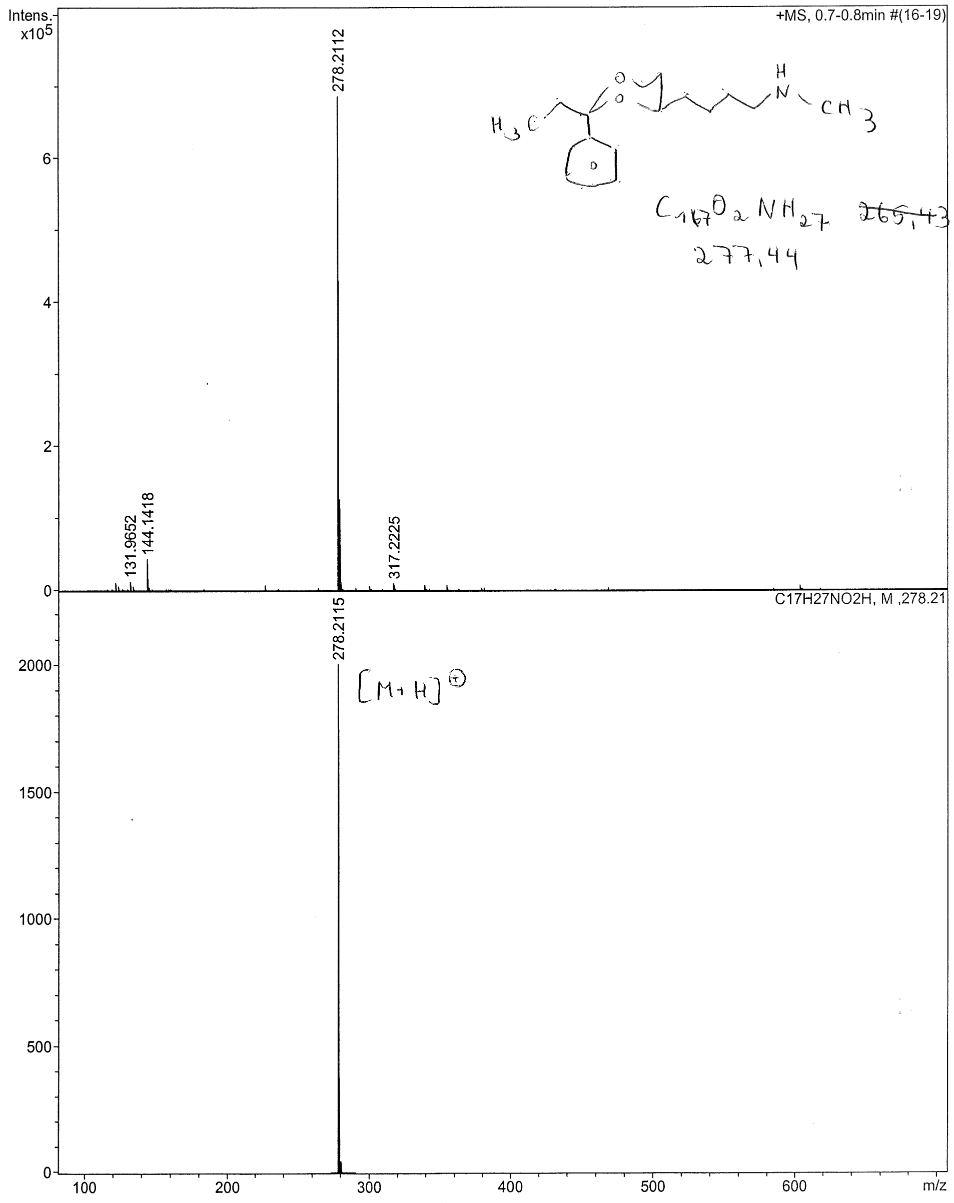

HPLC

Analyzed: 01.04.10 00:58

Reported: 01.04.10 08:24
Processed: 01.04.10 08:24

Data Path: D:\WIN32APP\HSM\Chromni\DATA\1377\
Application: Chromni
Sample Name: EM41A
Injection from this vial: 1 of 1

Series:1377
Vial Number: 9
Vial Type: UNK
Volume: 5,0 ul

Chrom Type: HPLC Channel : 1

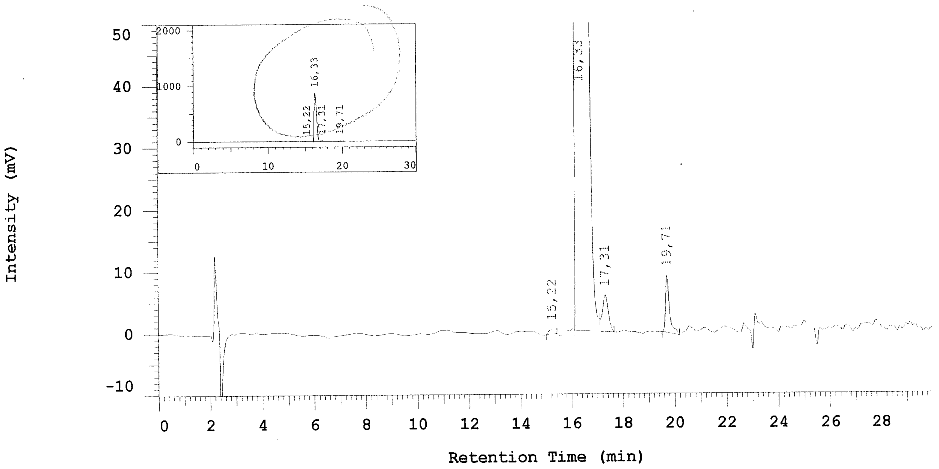

Acquisition Method: Chromni
Blank Subtr Sample Name: ACN
Column Type: 010
Developed by: Jens
Solvent A: Wasser + 0,05%TFA
Solvent B: ACN + 0,05%TFA

No.	RT	Area	Conc 1	BC
1	15,22	11276	0,063	MC
2	16,33	17777172	98,872	MC
3	17,31	95122	0,529	MC
4	19,71	96437	0,536	BB
		17980007	100,000	

Peak rejection level: 0

Spectroscopic and chromatographic data of compound **17a**

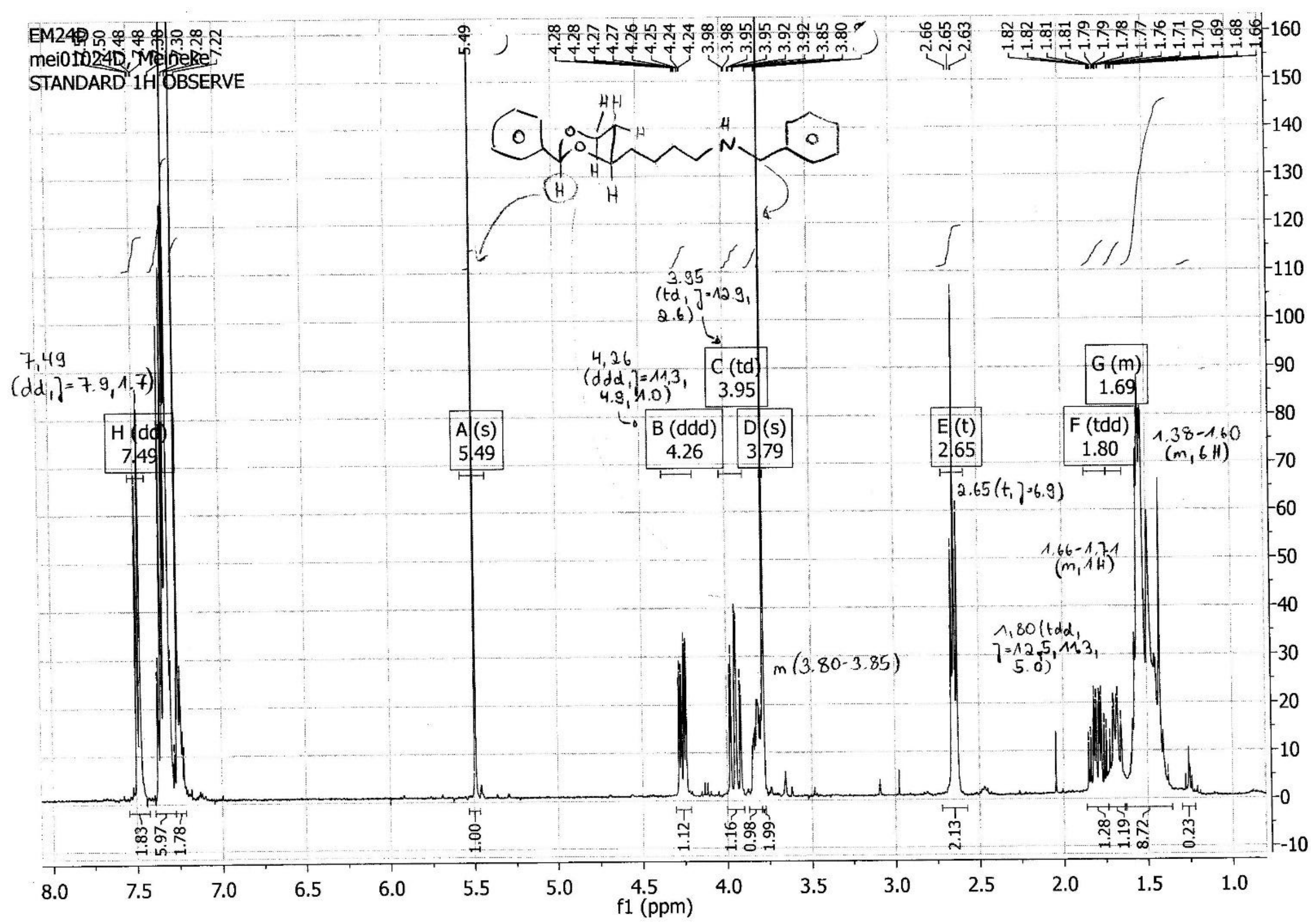

D:\Xcalibur\data\wme1211010 21.01.2010 09:05:58 EM24D

wme1211010
Type: Unknown ID: 1 Row: 1
Sample Name: EM24D
Study:
Client: Meineke
Laboratory: Meiners
Company:
Phone:
Instrument Method: D:\Xcalibur\methods\400ei.meth
Processing Method:
Vial: 1
Injection Volume (µl): 10,00
Sample Weight: 0,00
Sample Volume (µl): 0,00
ISTD Amount: 0,00
Dil Factor: 1,00

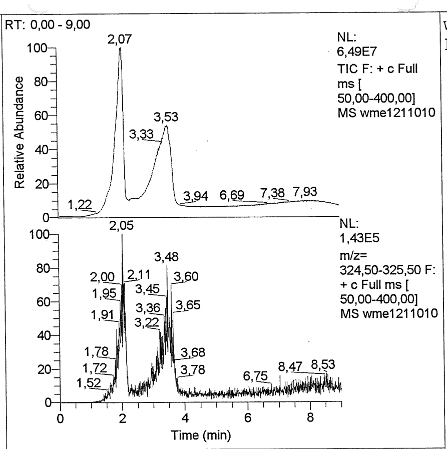

Mr=325,45

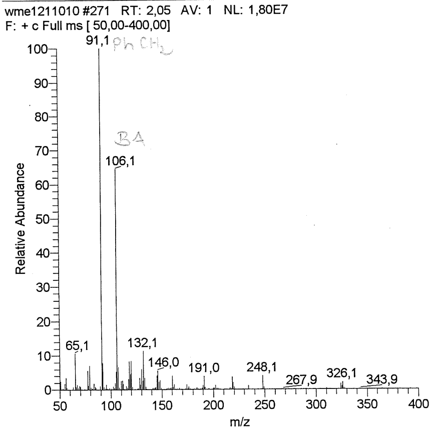

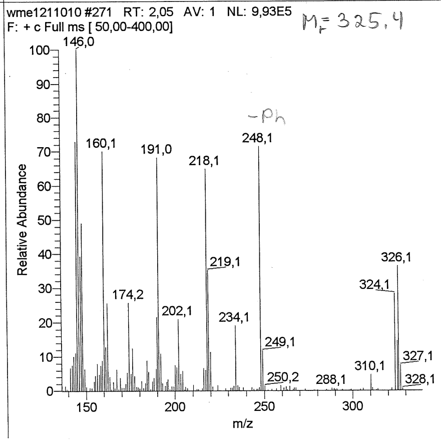

wme1211010#271 RT: 2,05
F: + c Full ms [50,00-400,00]

m/z	Intensity	Relative
50,9	432463,0	2,40
55,1	306442,0	1,70
56,1	625773,0	3,47
65,1	1910838,0	10,60
67,1	265418,0	1,47
77,1	983259,0	5,45
79,2	1248619,0	6,92
83,2	311427,0	1,73
91,1	18033664,0	100,00
92,2	1408809,0	7,81
95,2	260156,0	1,44
104,2	344198,0	1,91
105,1	952251,0	5,28
106,1	11636224,0	64,53
107,1	1172457,0	6,50
110,1	446426,0	2,48
111,2	461651,0	2,56
112,1	292665,0	1,62
117,1	543103,0	3,01
118,1	1490515,0	8,27
119,1	805981,0	4,47
120,0	1508180,0	8,36
128,1	607298,0	3,37
129,1	264569,0	1,47
130,1	1072798,0	5,95
131,1	469423,0	2,60
132,1	2023070,0	11,22
133,2	599510,0	3,32
145,0	723613,0	4,01
146,0	992791,0	5,51
147,1	389370,0	2,16
148,1	486080,0	2,70
160,1	695472,0	3,86
191,0	676904,0	3,75
218,1	643816,0	3,57
219,1	349662,0	1,94
248,1	709396,0	3,93
324,1	280846,0	1,56
326,1	359936,0	2,00

HPLC

Analyzed: 21.01.10 03:10

Reported: 21.01.10 13:40
Processed: 21.01.10 13:40

Data Path: D:\WIN32APP\HSM\Chromni\DATA\1070\
Application: Chromni

Series:1070

Sample Name: EM 24D

Vial Number: 10

Injection from this vial: 1 of 1

Vial Type: UNK
Volume: 5,0 ul

Chrom Type: HPLC Channel : 1

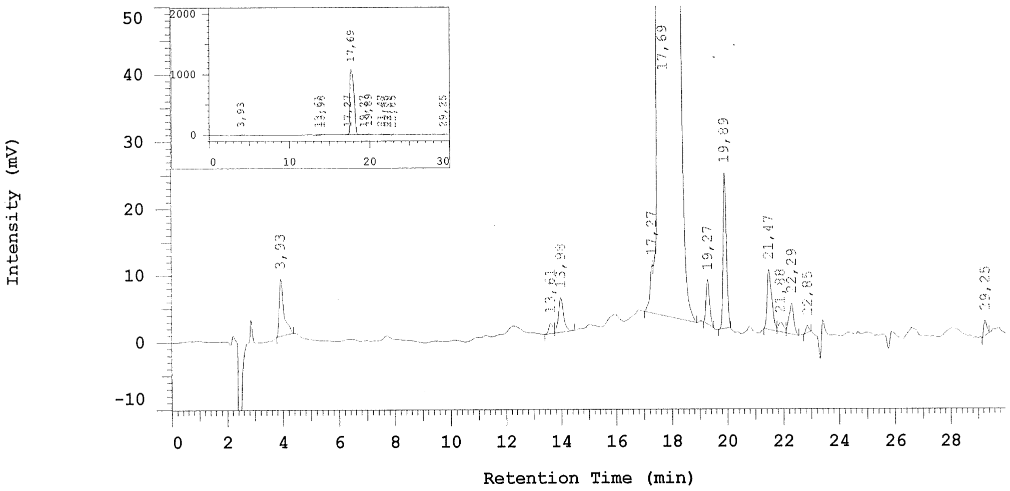

Acquisition Method: Chromni
Blank Subtr Sample Name: ACN
Column Type: 010

Developed by: Jens

Solvent A: Wasser + 0,05%TFA

Solvent B: ACN + 0,05%TFA

No.	RT	Area	Conc 1	BC
1	3,93	97263	0,259	MC
2	13,61	16038	0,043	MC
3	13,98	66401	0,177	BB
4	17,27	53055	0,141	MC
5	17,69	36850227	98,189	MC
6	19,27	58584	0,156	MC
7	19,89	188541	0,502	MC
8	21,47	101427	0,270	MC
9	21,88	19210	0,051	MC
10	22,29	54237	0,145	MC
11	22,85	7485	0,020	BB
12	29,25	17299	0,046	MC
		37529767	100,000	

Peak rejection level: 0

Spectroscopic and chromatographic data of compound **17b**

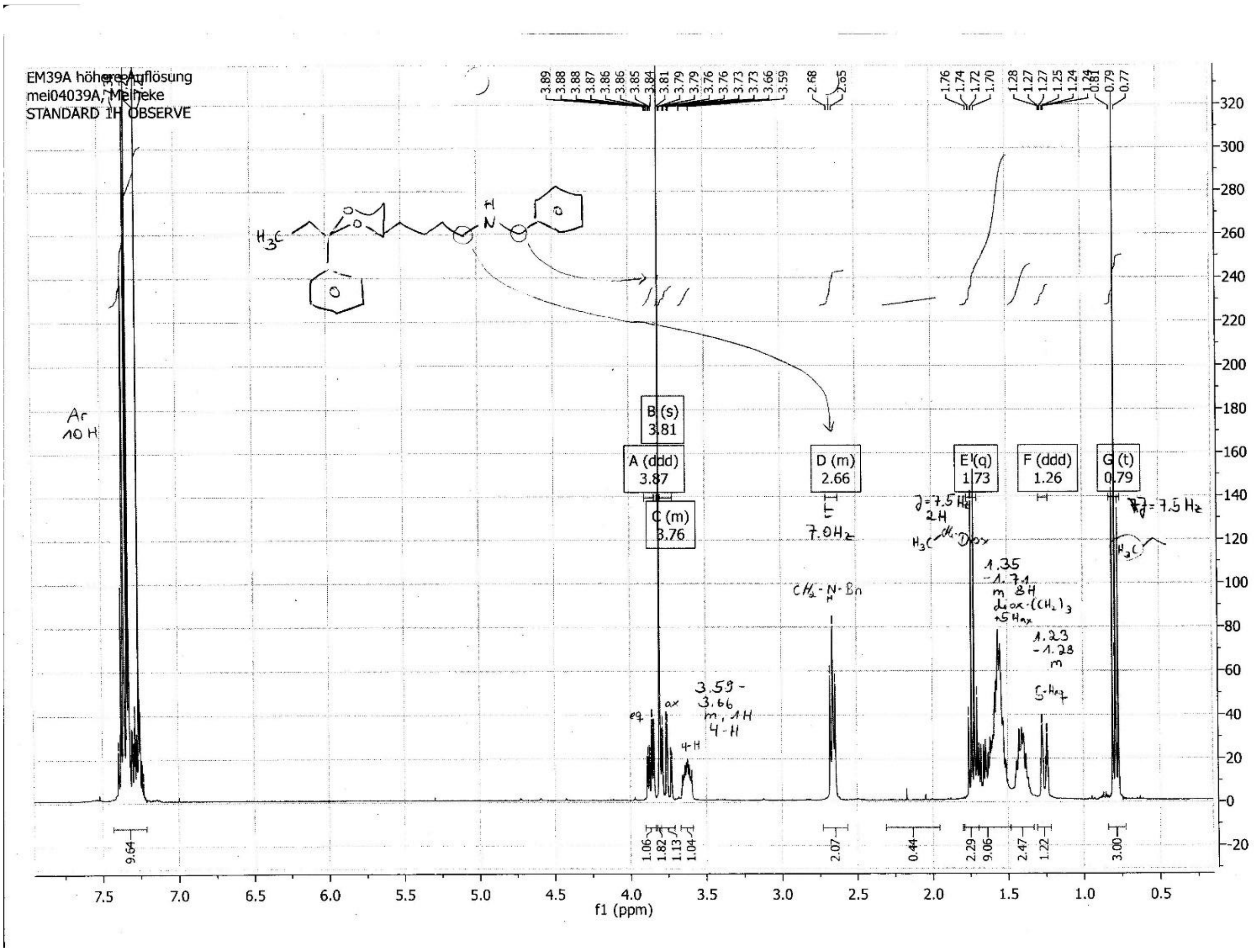

MicroTof (Bruker Daltonics) OC-Münster Abt. Massenspektrometrie

Analysis Info

Analysis Name Z:\Wuensch EM 39-a_acn_Tray1-73_01_113295.d

Sample Name Wuensch EM 39-a_acn

Comment MeOH pur

Acquisition Date 06.05.2010 13:46:57

Method oa_ms_pos_lm_ot.m

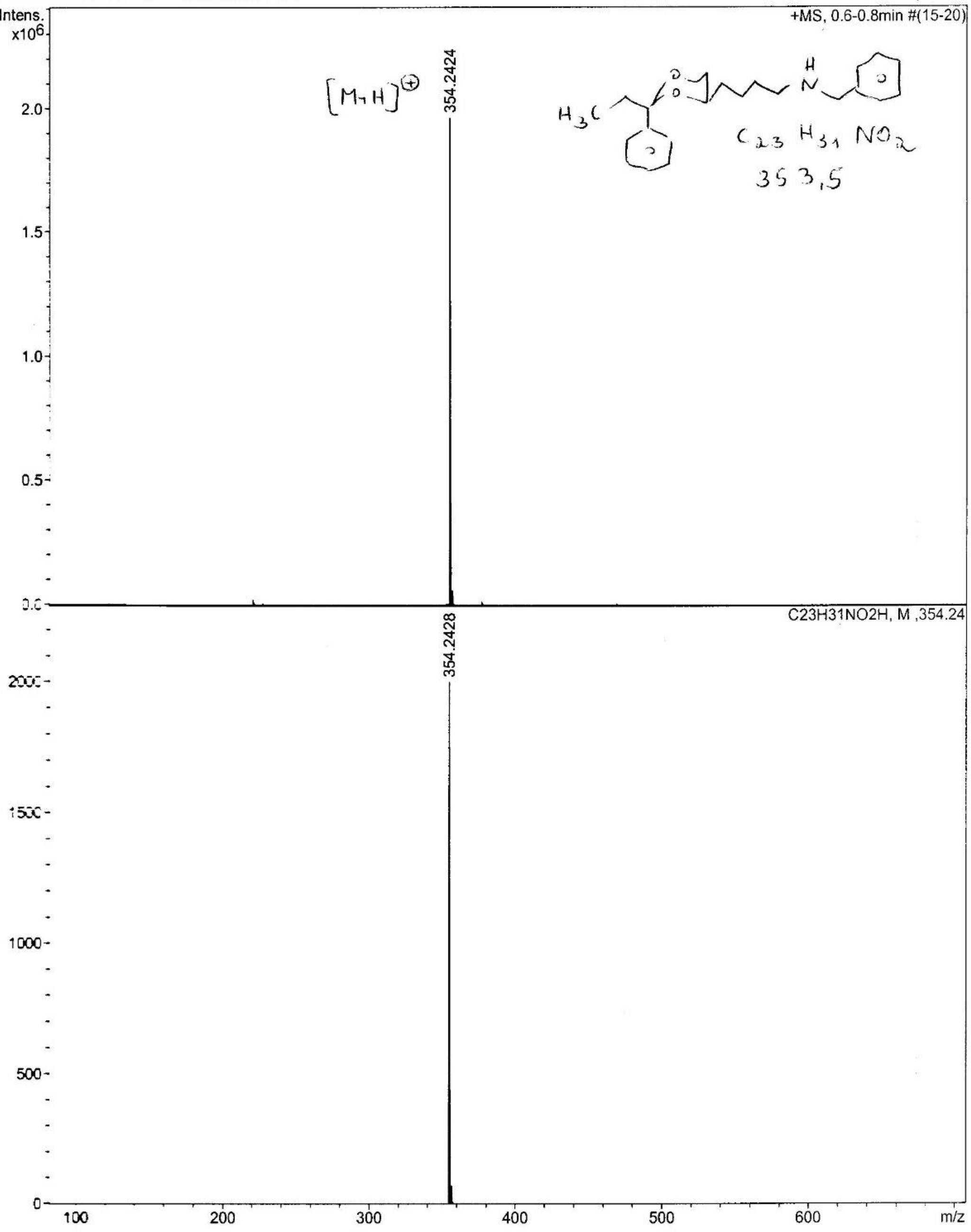

HPLC

Analyzed: 01.04.10 00:17

Reported: 01.04.10 08:21
Processed: 01.04.10 08:21

Data Path: D:\WIN32APP\HSM\Chromni\DATA\1376\
Application: Chromni
Sample Name: EM39A
Injection from this vial: 1 of 1

Series:1376
Vial Number: 8
Vial Type: UNK
Volume: 5,0 ul

Chrom Type: HPLC Channel : 1

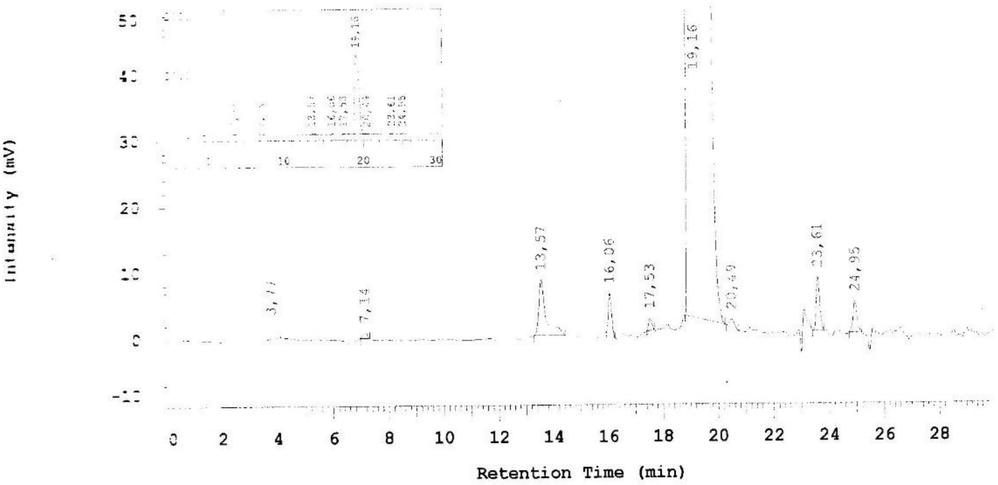

Acquisition Method: Chromni
Blank Subtr Sample Name: ACN
Column Type: C10
Solvent A: Wasser + 0,05%TFA

Developed by: Jens
Solvent B: ACN + 0,05%TFA

No.	RT	Area	Conc 1	BC
1	3,77	25989	0,055	MC
2	7,14	10048	0,021	BB
3	13,57	154525	0,327	MC
4	16,06	48781	0,103	MC
5	17,53	14706	0,031	MC
6	19,16	46816934	99,187	MC
7	20,49	15391	0,033	MC
8	23,61	67244	0,142	MC
9	24,95	46955	0,099	MC
		47200573	100,000	

Peak rejection level: 0

Spectroscopic and chromatographic data of compound **18a**

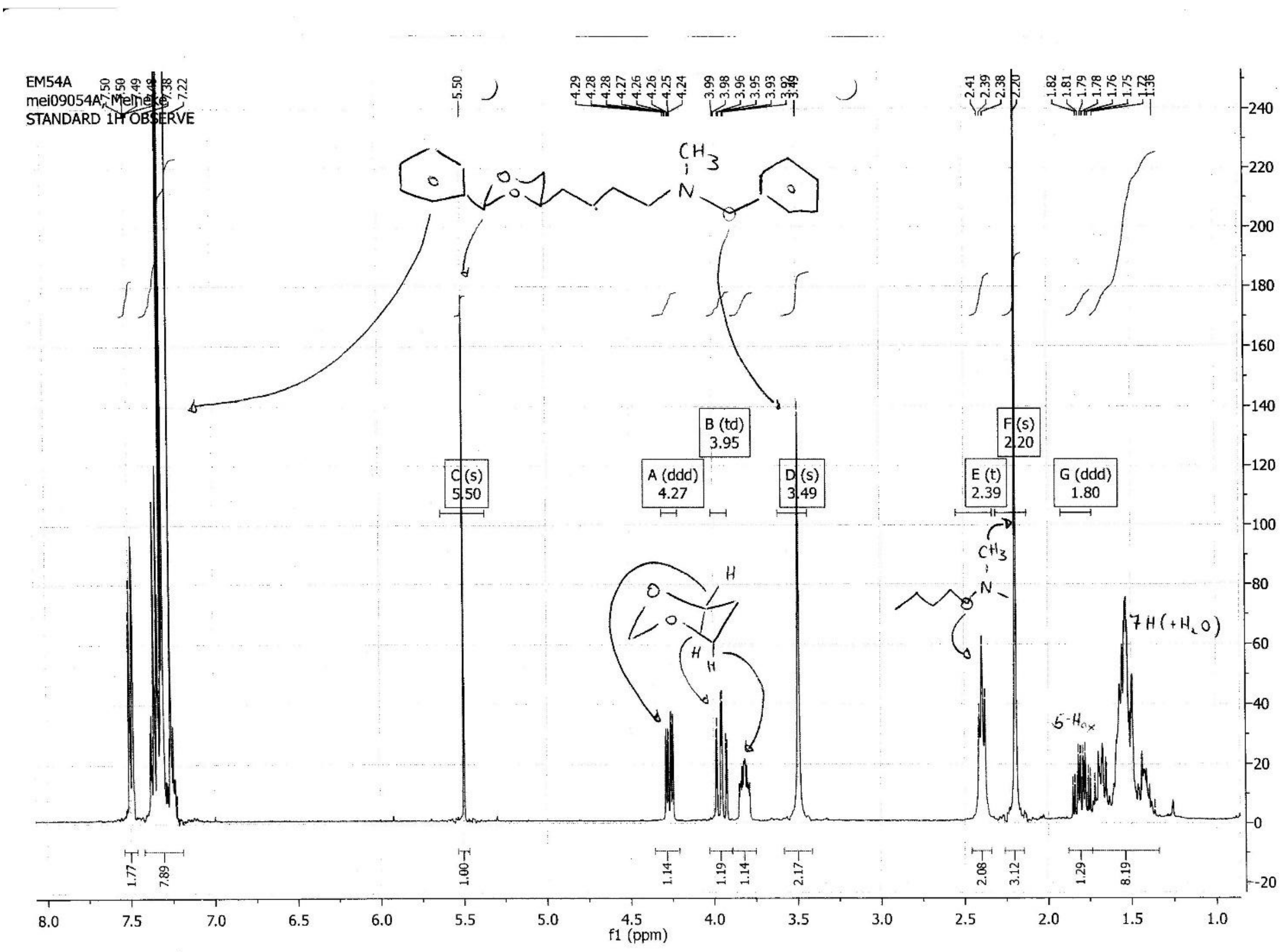

Generic Display Report

**Analysis Info**		Acquisition Date	9/24/2010 9:47:40 AM
Analysis Name	D:\Data\IPMC\PharmChemie\Routine\2010_09\WME_EM54A.d		
Method	directprobe_default.m	Operator	Schmidt
Sample Name	EM54A	Instrument	micrOTOF-Q II
Comment	Meineke<br>APCI-Direkt<br>Kalibration mit Fettsaeureestern		

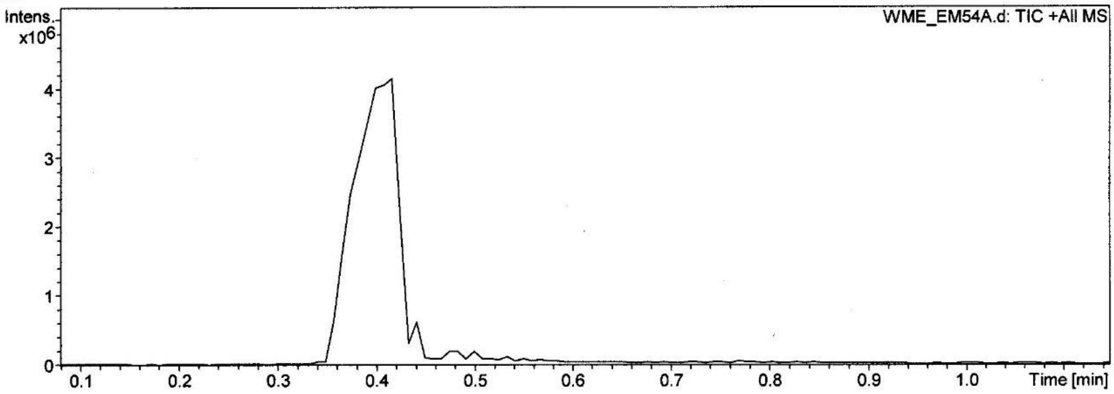

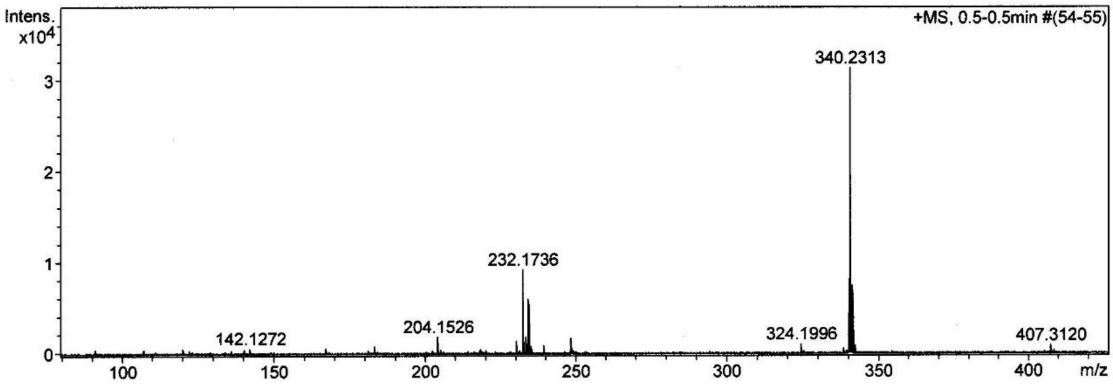

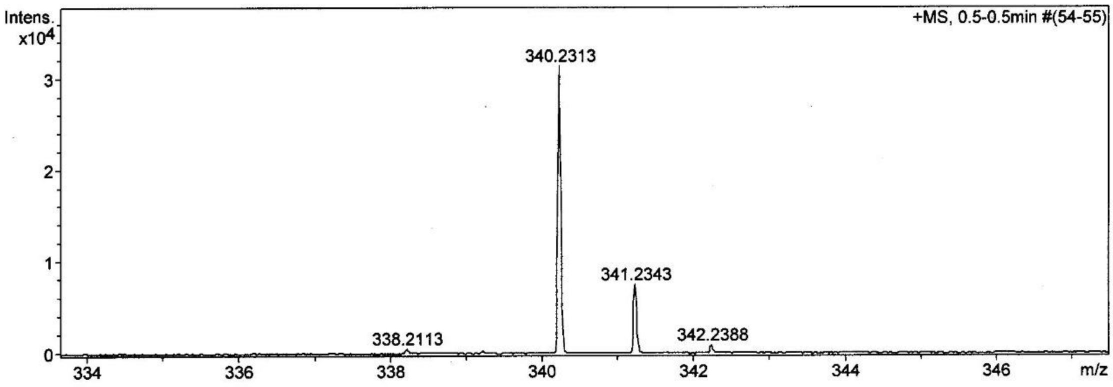

Mass Spectrum SmartFormula Report

Analysis Info

Analysis Name	D:\Data\IPMC\PharmChemie\Routine\2010_09\WME_EM54A.d	Acquisition Date	9/24/2010 9:47:40 AM
Method	directprobe_default.m	Operator	Schmidt
Sample Name	EM54A	Instrument / Ser#	micrOTOF-Q II 10252
Comment	Meineke<br>APCI-Direkt<br>Kalibration mit Fettsaeureestern		

Acquisition Parameter

Source Type	APCI	Ion Polarity	Positive	Set Nebulizer	1.0 Bar		
Focus	Not active	Set Capillary	4000 V	Set Dry Heater	200 °C		
Scan Begin	50 m/z	Set End Plate Offset	-500 V	Set Dry Gas	3.0 l/min		
Scan End	1000 m/z	Set Collision Cell RF	130.0 Vpp	Set Divert Valve	Waste		

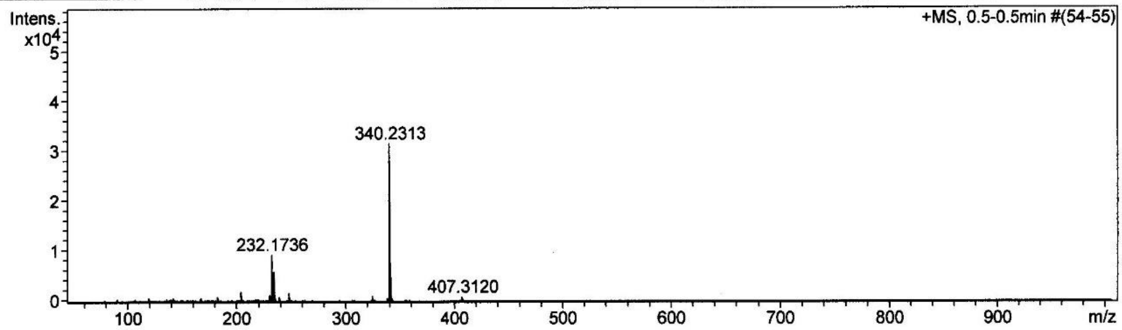

Meas. m/z	#	Formula	Score	m/z	err [mDa]	err [ppm]	mSigma	rdb	e⁻ Conf	N-Rule
340.2313	1	C 22 H 30 N O 2	19.08	340.2271	-4.2	-12.4	3.1	8.5	even	ok
	2	C 16 H 30 N 5 O 3	45.17	340.2343	3.0	8.8	27.2	4.5	even	ok
	3	C 15 H 34 N O 7	100.00	340.2330	1.7	4.9	39.7	-0.5	even	ok
	4	C 23 H 31 O 2	0.00	339.2319	10.2	29.9	725.9	8.5	even	ok
	5	C 19 H 27 N 6	0.00	339.2292	7.5	22.0	735.4	9.5	even	ok
	6	C 18 H 31 N 2 O 4	0.00	339.2278	6.1	18.1	742.9	4.5	even	ok
	7	C 15 H 28 N 7 O 2	0.00	338.2299	18.6	55.0	808.1	5.5	even	ok
	8	C 21 H 28 N 3 O	0.00	338.2227	11.4	33.7	809.7	9.5	even	ok

HPLC

Analyzed: 23.09.10 07:19

Data Path: D:\WIN32APP\HSM\Chromni\DATA\2179\
Application: Chromni
Sample Name: EM54A
Injection from this vial: 1 of 1

Reported: 23.09.10 15:13
Processed: 23.09.10 15:13

Series:2179
Vial Number: 17
Vial Type: UNK
Volume: 5,0 ul

Chrom Type: HPLC Channel : 1

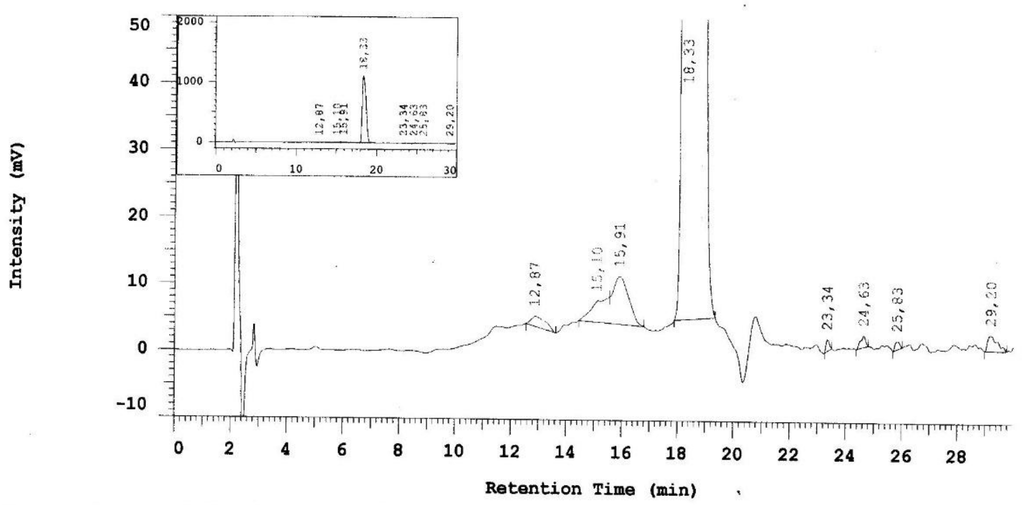

Acquisition Method: Chromni
Blank Subtr Sample Name: ACN
Column Type: 010
Solvent A: Wasser + 0,05%TFA

Developed by: Jens
Solvent B: ACN + 0,05%TFA

No.	RT	Area	Conc 1	BC
1	12,87	58235	0,166	MC
2	15,10	144187	0,411	MC
3	15,91	284930	0,812	MC
4	18,33	34515856	98,308	BB
5	23,34	14056	0,040	MC
6	24,63	21962	0,063	BB
7	25,83	13433	0,038	BB
8	29,20	57185	0,163	BB
		35109844	100,000	

Peak rejection level: 0

Spectroscopic and chromatographic data of compound **18b**

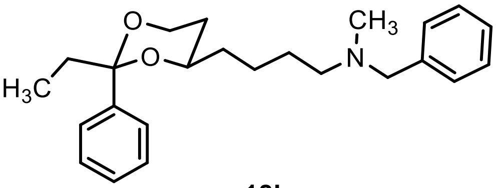

18b

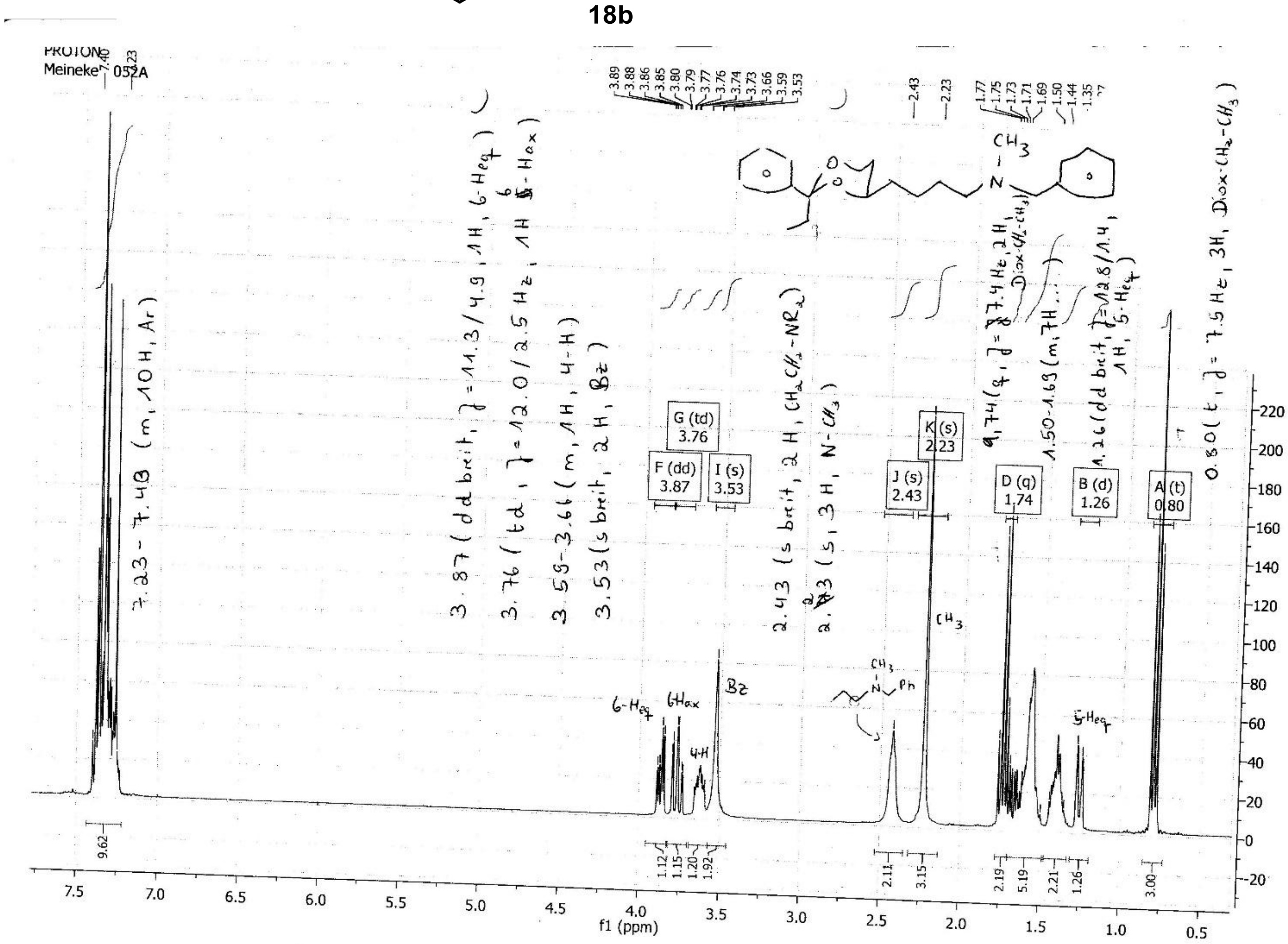

Generic Display Report

**Analysis Info**		Acquisition Date	9/17/2010 11:14:54 AM
Analysis Name	D:\Data\IPMC\PharmChemie\Routine\2010_09\WME_EM52A.d		
Method	directprobe_default.m	Operator	Schmidt
Sample Name	EM52A	Instrument	micrOTOF-Q II
Comment	Meineke<br>APCI-Direkt<br>Kalibration mit Fettsaeureestern		

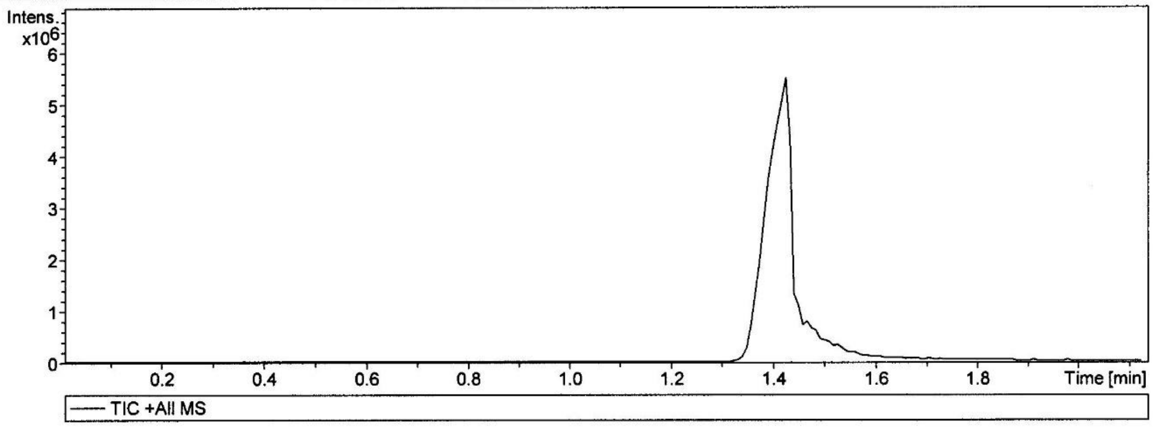

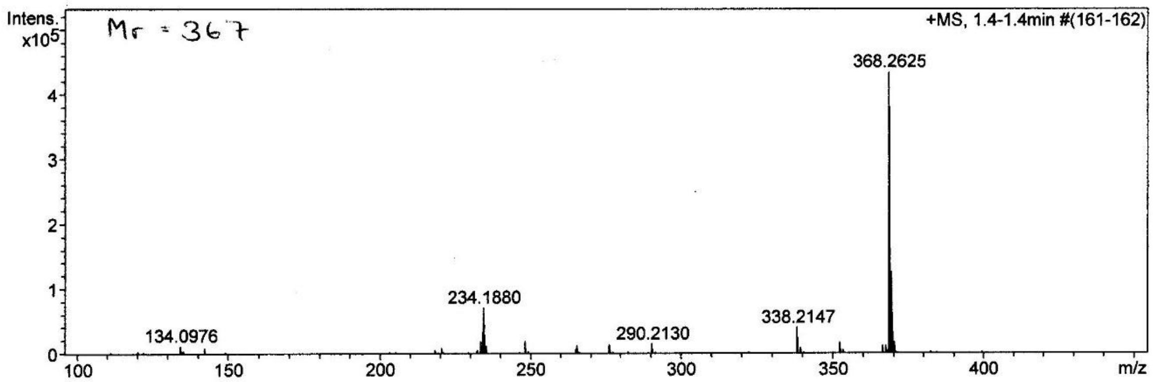

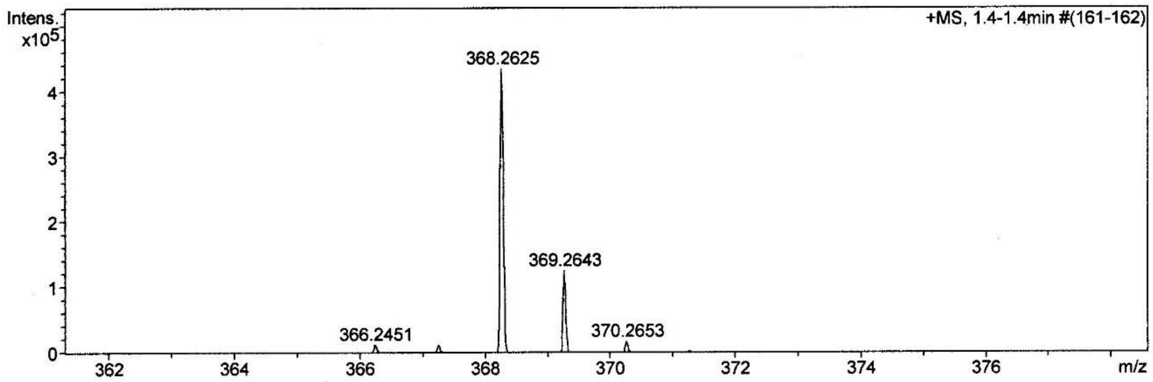

Mass Spectrum SmartFormula Report

Analysis Info

Analysis Name	D:\Data\IPMC\PharmChemie\Routine\2010_09\WME_EM52A.d	Acquisition Date	9/17/2010 11:14:54 AM
Method	directprobe_default.m	Operator	Schmidt
Sample Name	EM52A	Instrument / Ser#	micrOTOF-Q II 10252
Comment	Meineke<br>APCI-Direkt<br>Kalibration mit Fettsaeureestern		

Acquisition Parameter

Source Type	APCI	Ion Polarity	Positive	Set Nebulizer	1.0 Bar
Focus	Not active	Set Capillary	4000 V	Set Dry Heater	200 °C
Scan Begin	50 m/z	Set End Plate Offset	-500 V	Set Dry Gas	3.0 l/min
Scan End	1000 m/z	Set Collision Cell RF	130.0 Vpp	Set Divert Valve	Waste

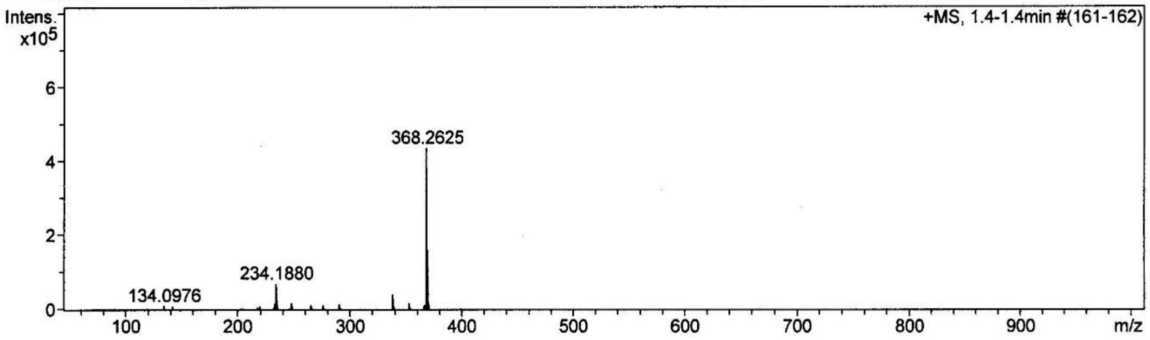

Meas. m/z	#	Formula	Score	m/z	err [mDa]	err [ppm]	mSigma	rdb	e⁻ Conf	N-Rule
368.2625	1	C 24 H 34 N O 2	100.00	368.2584	-4.1	-11.2	12.3	8.5	even	ok

HPLC

Analyzed: 22.09.10 21:38

Reported: 23.09.10 14:28
Processed: 23.09.10 14:28

Data Path: D:\WIN32APP\HSM\Chromni\DATA\2165\
Application: Chromni

Series:2165

Sample Name: EM52A
Injection from this vial: 1 of 1

Vial Number: 5
Vial Type: UNK
Volume: 5,0 ul

Chrom Type: HPLC Channel : 1

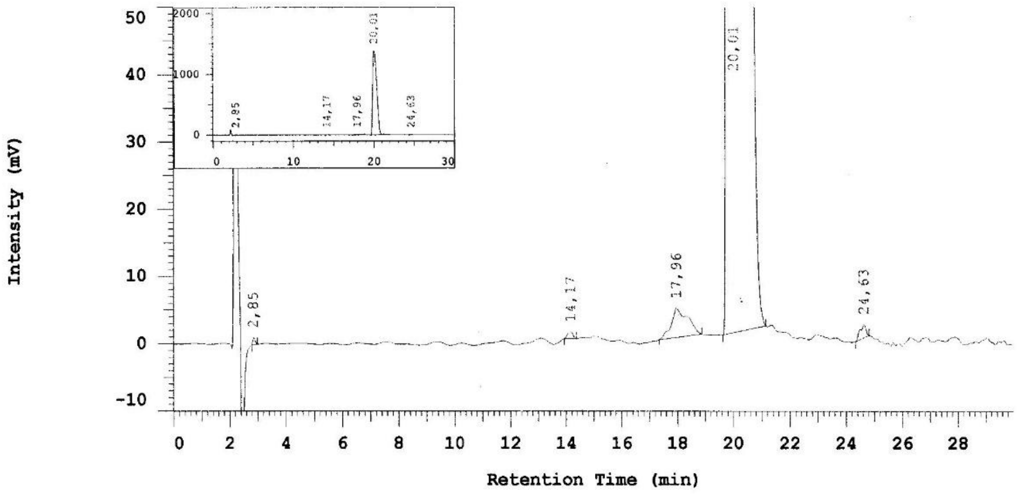

Acquisition Method: Chromni
Blank Subtr Sample Name: ACN
Column Type: 010

Developed by: Jens

Solvent A: Wasser + 0,05%TFA

Solvent B: ACN + 0,05%TFA

No.	RT	Area	Conc 1	BC
1	2,85	5027	0,011	MC
2	14,17	13543	0,029	BB
3	17,96	175602	0,378	MC
4	20,01	46186841	99,520	BB
5	24,63	28700	0,062	BB
		46409713	100,000	

Peak rejection level: 0

Spectroscopic and chromatographic data of compound **19a**

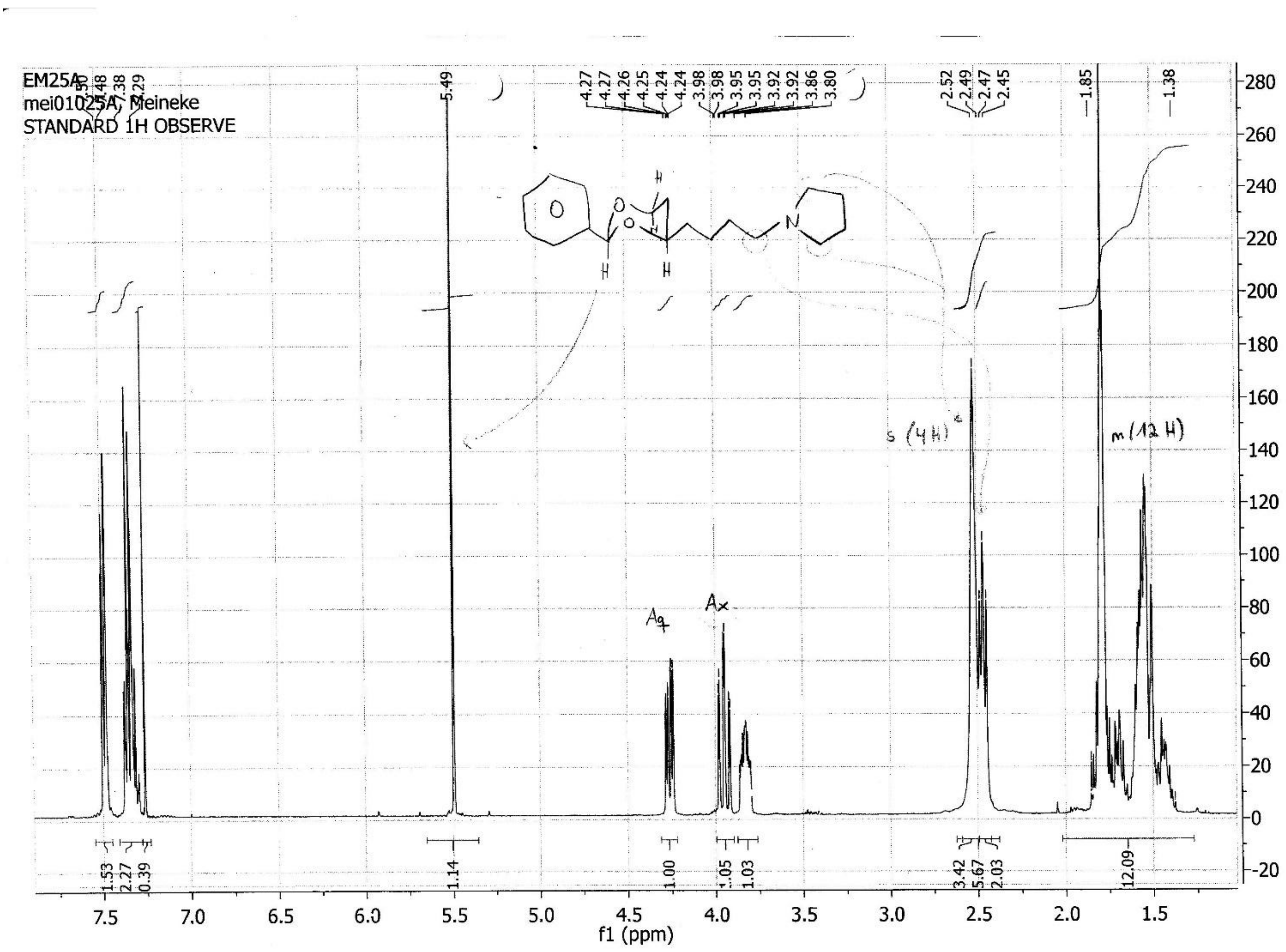

D:\Xcalibur\data\wme2260110 26.01.2010 14:01:15 EM25A

```
wme2260110
Type: Unknown ID: 1 Row: 1
Sample Name:              EM25A
Study:
Client:                   Meineke
Laboratory:               Meiners
Company:
Phone:
Instrument Method:        D:\Xcalibur\methods\400ei.meth
Processing Method:
Vial:                     1
Injection Volume (µl):    10,00
Sample Weight:            0,00
Sample Volume (µl):       0,00
ISTD Amount:              0,00
Dil Factor:               1,00
```

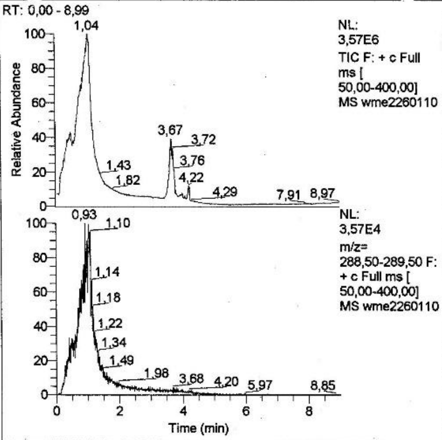

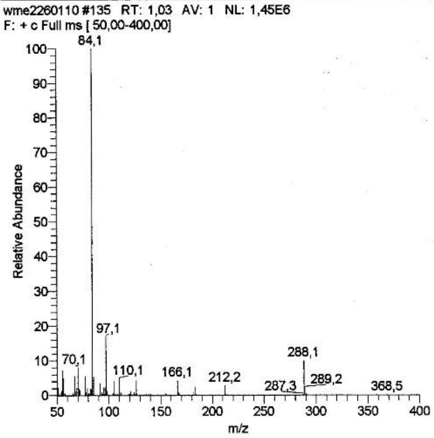

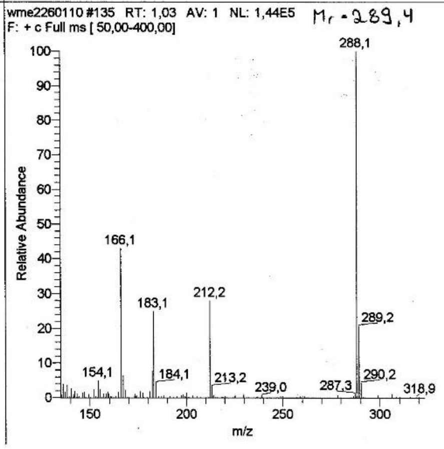

wme2260110#135 RT: 1,03
F: + c Full ms [50,00-400,00]

m/z	Intensity	Relative
50,9	32943,0	2,26
54,1	18912,0	1,30
55,1	106804,0	7,34
56,1	72563,0	4,99
65,1	10816,0	0,74
67,1	79570,0	5,47
68,1	17822,0	1,23
69,1	29865,0	2,05
70,1	115688,0	7,95
71,2	26929,0	1,85
72,2	20568,0	1,41
77,1	82159,0	5,65
78,2	12103,0	0,83
79,1	30638,0	2,11
82,1	26438,0	1,82
83,1	26001,0	1,79
84,1	1454462,0	100,00
85,2	76889,0	5,29
91,1	50529,0	3,47
93,1	13033,0	0,90
95,1	33551,0	2,31
96,1	33189,0	2,28
97,1	245905,0	16,91
98,1	19387,0	1,33
105,1	58936,0	4,05
110,1	69463,0	4,78
111,2	12637,0	0,87
112,1	9580,0	0,66
121,1	15458,0	1,06
124,1	11921,0	0,82
126,1	62064,0	4,27
127,2	9609,0	0,66
166,1	61766,0	4,25
167,2	9441,0	0,65
182,1	11166,0	0,77
183,1	35799,0	2,46
212,2	40140,0	2,76
288,1	143760,0	9,88
289,2	29927,0	2,06

HPLC

Analyzed: 27.01.10 23:28

Reported: 28.01.10 13:06
Processed: 28.01.10 13:06

Data Path: D:\WIN32APP\HSM\Chromni\DATA\1090\
Application: Chromni
Sample Name: EM25A
Injection from this vial: 1 of 1

Series:1090
Vial Number: 7
Vial Type: UNK
Volume: 5,0 ul

Chrom Type: HPLC Channel : 1

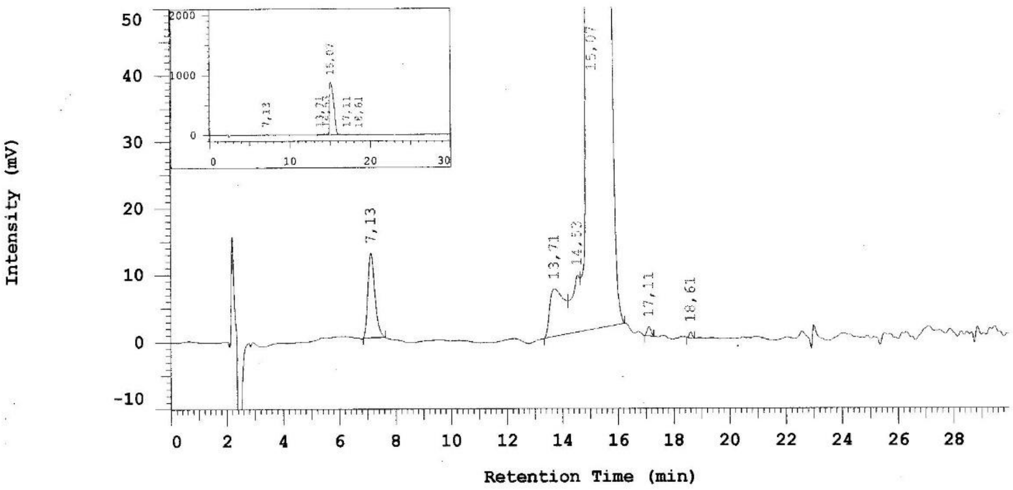

Acquisition Method: Chromni
Blank Subtr Sample Name: ACN
Column Type: 010
Developed by: Jens
Solvent A: Wasser + 0,05%TFA
Solvent B: ACN + 0,05%TFA

No.	RT	Area	Conc 1	BC
1	7,13	212220	0,659	MC
2	13,71	253607	0,788	BV
3	14,53	167850	0,521	MC
4	15,07	31543187	97,974	VB
5	17,11	12612	0,039	BB
6	18,61	6101	0,019	BB
		32195577	100,000	

Peak rejection level: 0

Spectroscopic and chromatographic data of compound **19b**

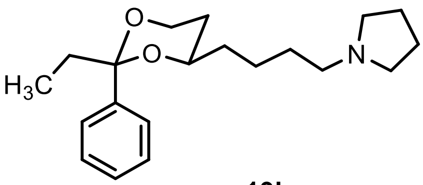

19b

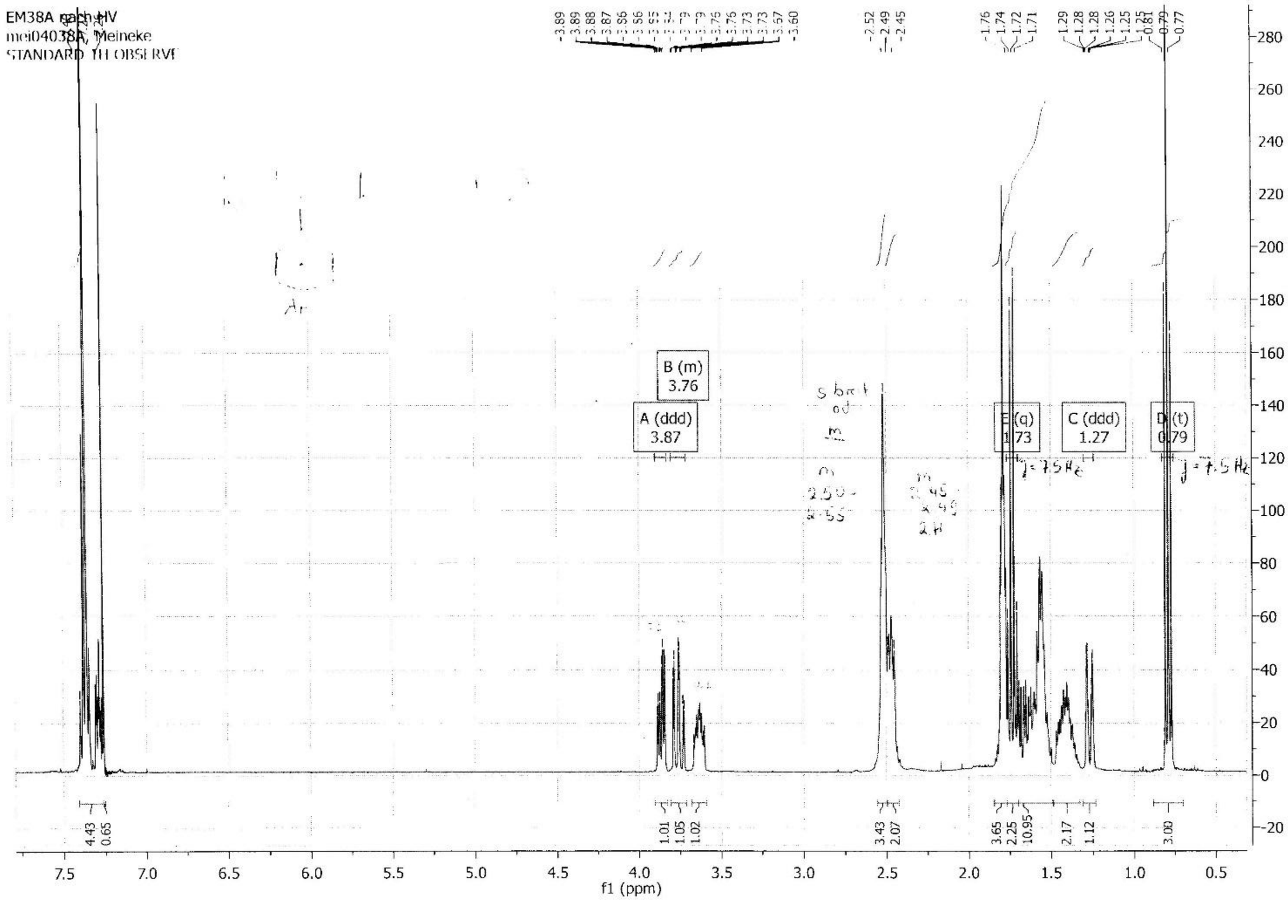

D:\Xcalibur\data\wme3220310 22.03.2010 11:47:13 EM38A

wme3220310
Type: Unknown ID: 1 Row: 1
Sample Name: EM38A
Study:
Client: Meineke
Laboratory: Meiners
Company:
Phone:
Instrument Method: D:\Xcalibur\methods\400ei.meth
Processing Method:
Vial: 1
Injection Volume (µl): 10,00
Sample Weight: 0,00
Sample Volume (µl): 0,00
ISTD Amount: 0,00
Dil Factor: 1,00

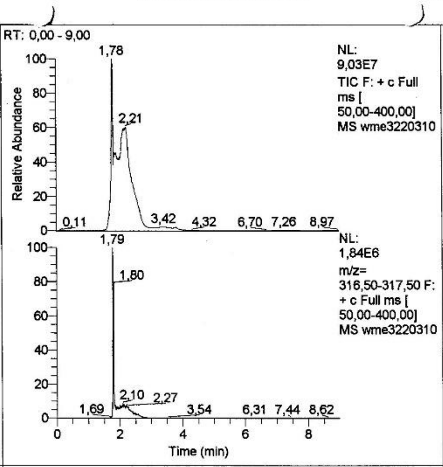

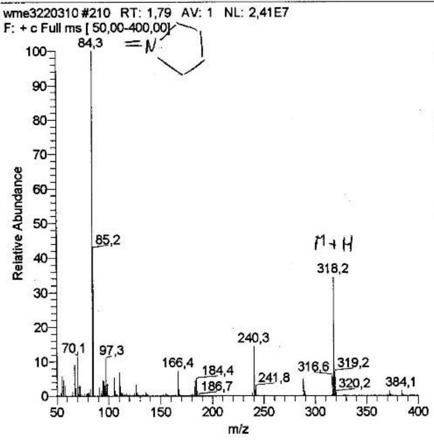

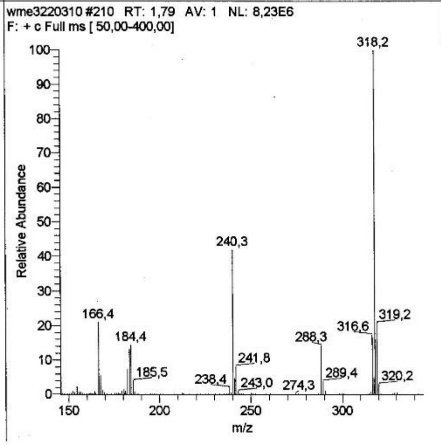

wme3220310#210 RT: 1,79
F: + c Full ms [50,00-400,00]

m/z	Intensity	Relative
55,2	1388856,0	5,77
56,2	1119825,0	4,65
57,5	762060,0	3,17
67,2	2201869,0	9,15
68,3	528774,0	2,20
70,1	2759071,0	11,46
71,3	729601,0	3,03
72,5	698732,0	2,90
82,1	475537,0	1,98
84,3	24067328,0	100,00
85,2	10296064,0	42,78
91,3	591032,0	2,46
93,2	379695,0	1,58
94,3	1067732,0	4,44
95,3	1059902,0	4,40
96,2	739768,0	3,07
97,3	2607041,0	10,83
98,0	345507,0	1,44
98,7	867115,0	3,60
105,3	1290356,0	5,36
106,3	357393,0	1,48
110,3	1657440,0	6,89
111,3	679711,0	2,82
126,4	786197,0	3,27
166,4	1723123,0	7,16
167,6	450452,0	1,87
182,3	602147,0	2,50
183,3	1087095,0	4,52
184,4	1182622,0	4,91
185,5	334397,0	1,39
240,3	3445986,0	14,32
241,2	390696,0	1,62
241,8	674706,0	2,80
288,3	1181221,0	4,91
316,6	1426948,0	5,93
317,5	417151,0	1,73
318,2	8233824,0	34,21
319,2	1706927,0	7,09
384,1	330367,0	1,37

HPLC

Analyzed: 18.03.10 21:05

Reported: 19.03.10 13:10
Processed: 19.03.10 13:09

Data Path: D:\WIN32APP\HSM\Chromni\DATA\1318\
Application: Chromni
Sample Name: EM38A
Injection from this vial: 1 of 1

Series:1318
Vial Number: 4
Vial Type: UNK
Volume: 5,0 ul

Chrom Type: HPLC Channel : 1

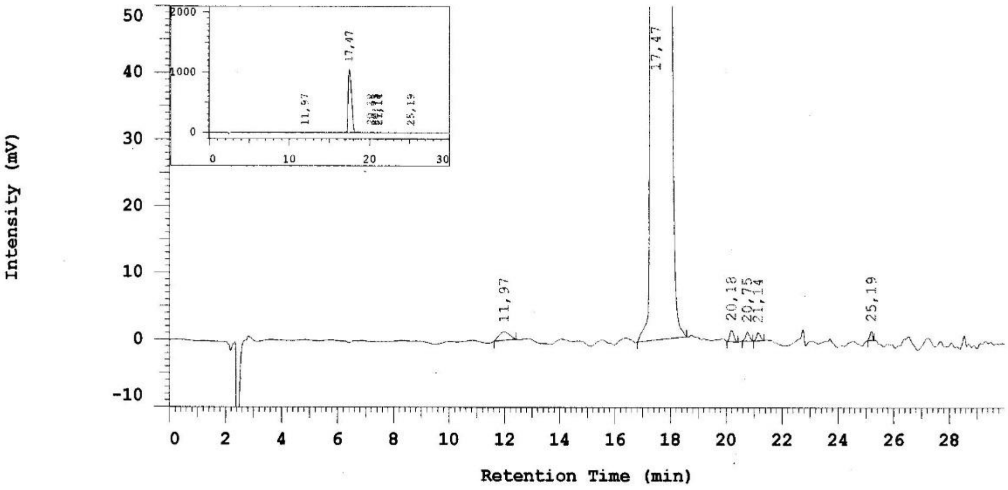

Acquisition Method: Chromni
Blank Subtr Sample Name: ACN
Column Type: 010
Solvent A: Wasser + 0,05%TFA

Developed by: Jens
Solvent B: ACN + 0,05%TFA

No.	RT	Area	Conc 1	BC
1	11,97	32749	0,112	MC
2	17,47	29099971	99,714	MC
3	20,18	17414	0,060	BB
4	20,75	13558	0,046	MC
5	21,14	11159	0,038	MC
6	25,19	8506	0,029	MC
		29183357	100,000	

Peak rejection level: 0

Spectroscopic and chromatographic data of compound **24a**

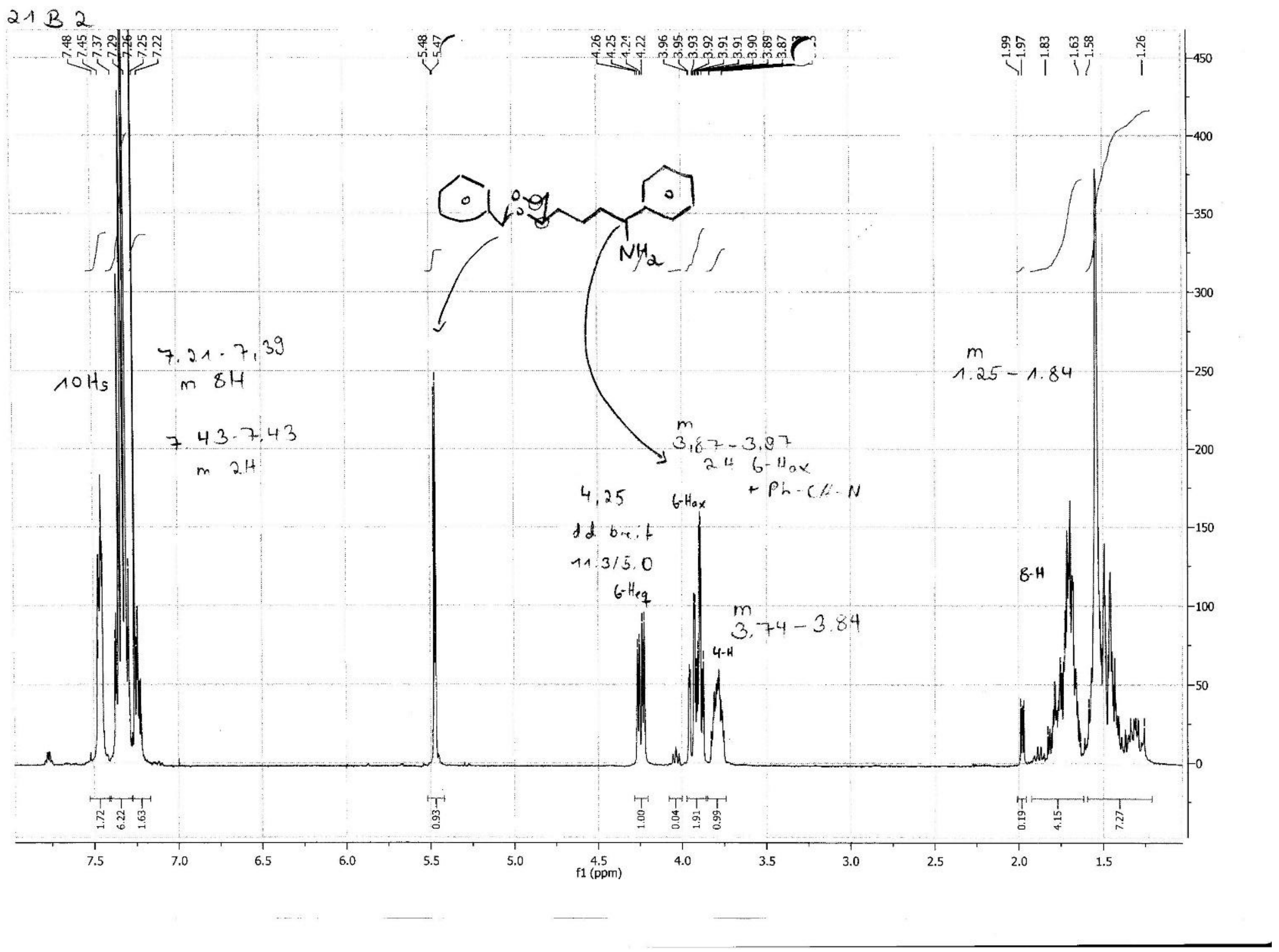

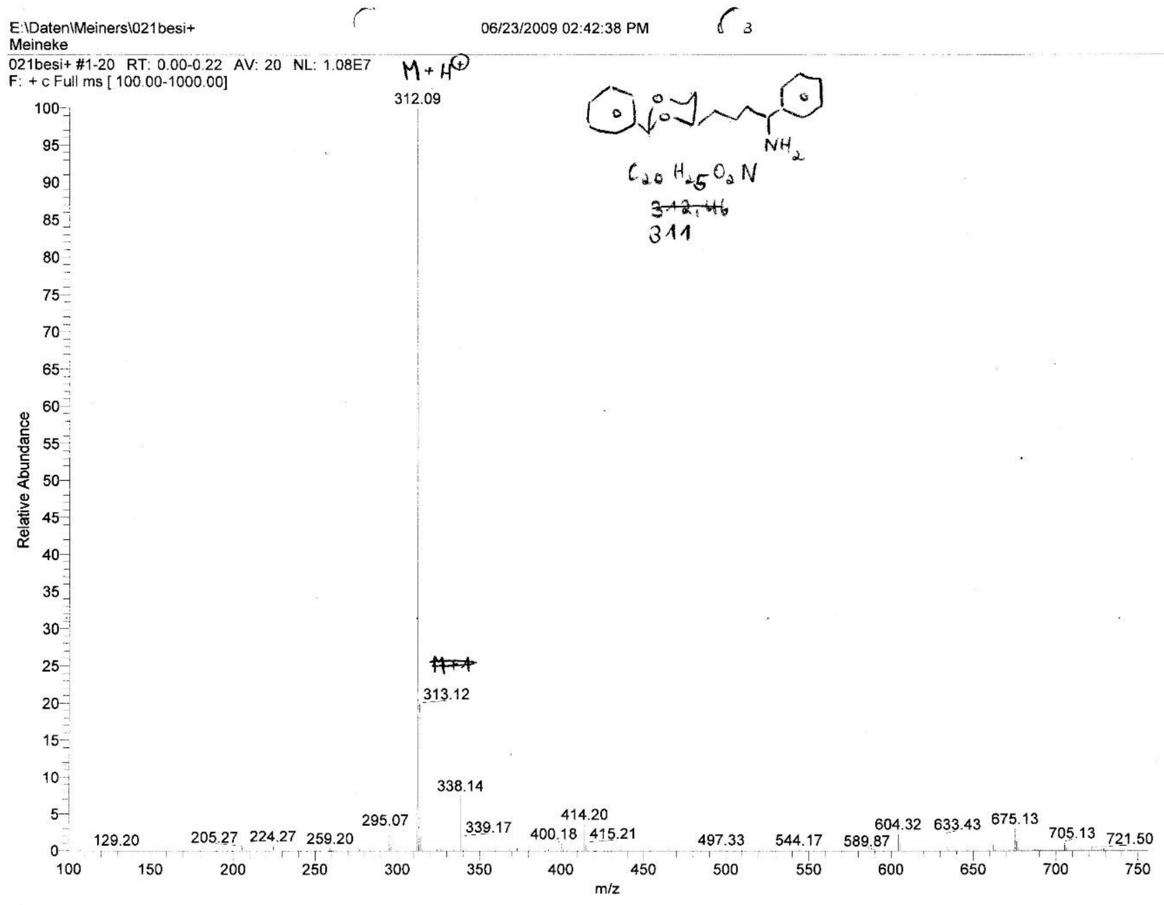

E:\Daten\Meiners\021besi+
Meineke
06/23/2009 02:42:38 PM
021besi+ #1-20 RT: 0.00-0.22 AV: 20 NL: 1.08E7
F: + c Full ms [100.00-1000.00]
M+H+
312.09
313.12
338.14
295.07
339.17
414.20
400.18
415.21
129.20
205.27
224.27
259.20
497.33
544.17
589.87
604.32
633.43
675.13
705.13
721.50
Relative Abundance
m/z
NH2
C20 H25 O2 N
311

nach 3 Säule
↳ Abgabe!

HPLC

Analyzed: 25.06.09 03:59

Reported: 25.06.09 08:54
Processed: 25.06.09 08:54

Data Path: D:\WIN32APP\HSM\Chromni\DATA\0339\
Application: Chromni
Sample Name: EM21B
Injection from this vial: 1 of 1

Series:0339
Vial Number: 11
Vial Type: UNK
Volume: 5,0 ul

Chrom Type: HPLC Channel : 1

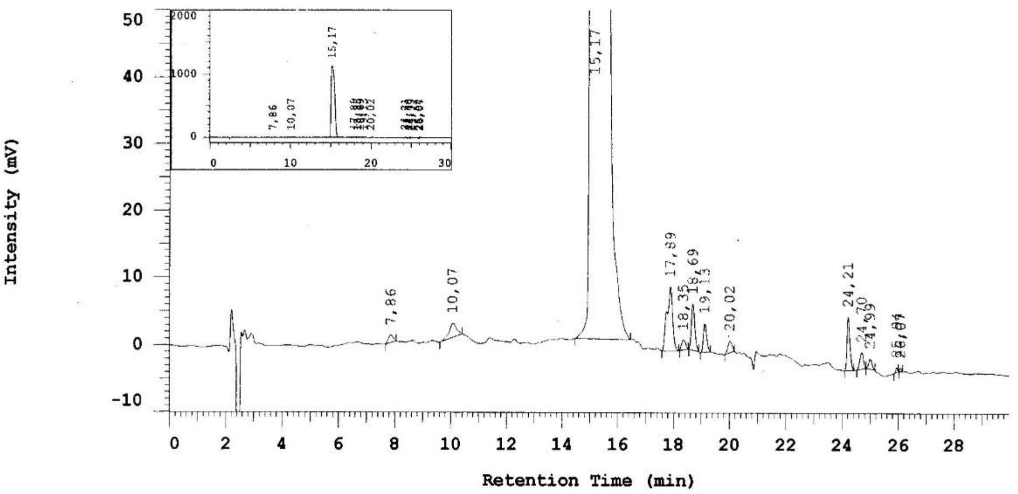

Acquisition Method: Chromni MeOH
Blank Subtr Sample Name: MeOH
Column Type: 010
Solvent A: Wasser + 0,05%TFA

Developed by: Christian
Solvent C: MeOH +0,05% TFA

No.	RT	Area	Conc 1	BC
1	7,86	12672	0,039	MC
2	10,07	37611	0,115	MC
3	15,17	32445267	98,785	MC
4	17,89	131294	0,400	BB
5	18,35	14466	0,044	MC
6	18,69	56707	0,173	MC
7	19,13	34628	0,105	BB
8	20,02	17159	0,052	MC
9	24,21	55237	0,168	MC
10	24,70	21783	0,066	MC
11	24,99	12570	0,038	MC
12	25,94	2749	0,008	MC
13	26,07	2205	0,007	MC
		32844348	100,000	

Peak rejection level: 0

Spectroscopic and chromatographic data of compound **24b**

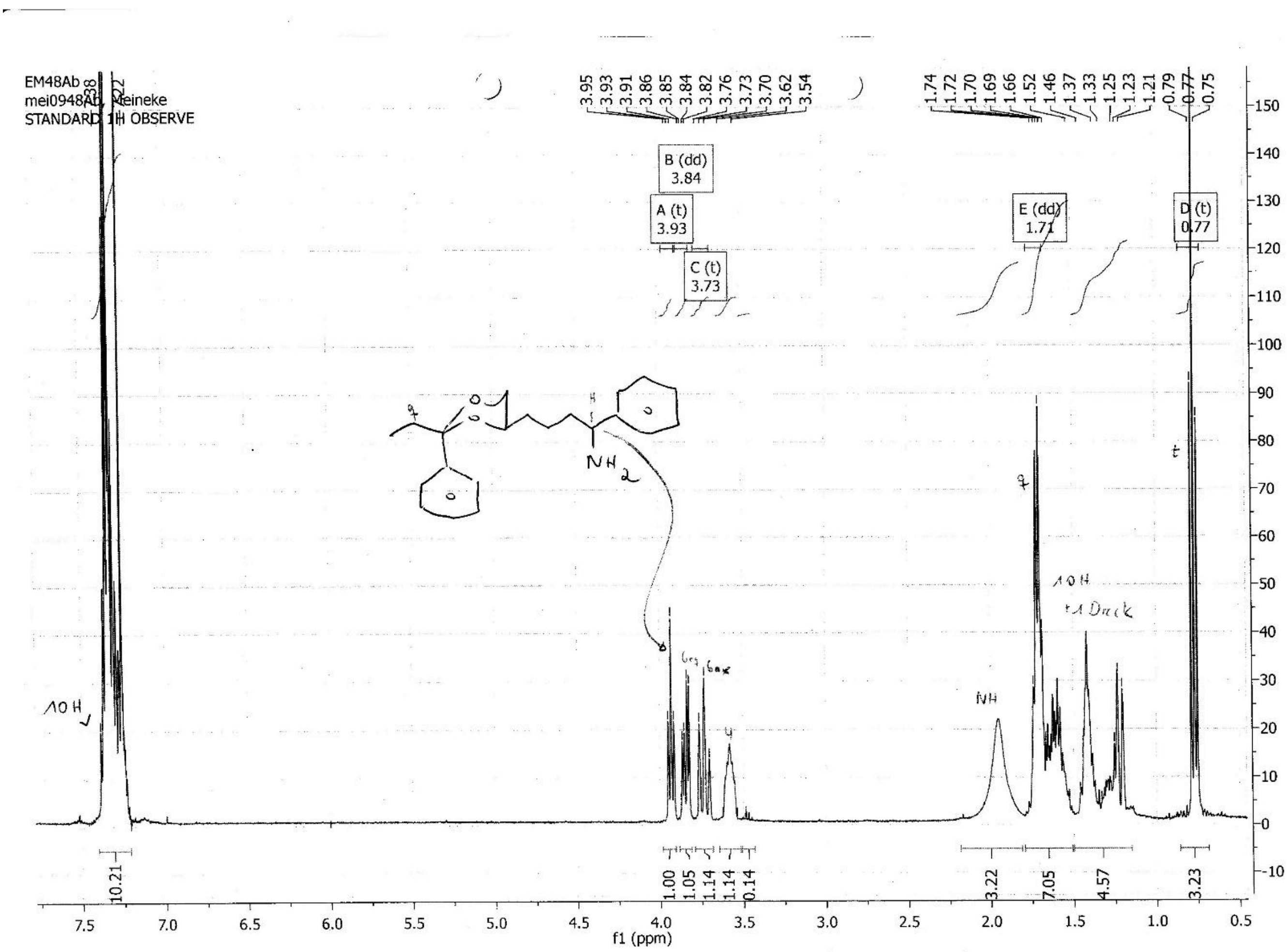

Mass Spectrum SmartFormula Report

Analysis Info

Analysis Name	D:\Data\IPMC\PharmChemie\Routine\2019_10\WME_EM48Ab.d	Acquisition Date	10/7/2010 1:26:10 PM
Method	directprobe_default.m	Operator	Meiners
Sample Name	EM48Ab	Instrument / Ser#	micrOTOF-Q II 10252
Comment	Meineke<br>APCI-Direkt<br>Kalibration mit APCI Calibrant Solution		

Acquisition Parameter

Source Type	APCI	Ion Polarity	Positive	Set Nebulizer	1.0 Bar
Focus	Not active	Set Capillary	4000 V	Set Dry Heater	200 °C
Scan Begin	50 m/z	Set End Plate Offset	-500 V	Set Dry Gas	3.0 l/min
Scan End	1000 m/z	Set Collision Cell RF	130.0 Vpp	Set Divert Valve	Waste

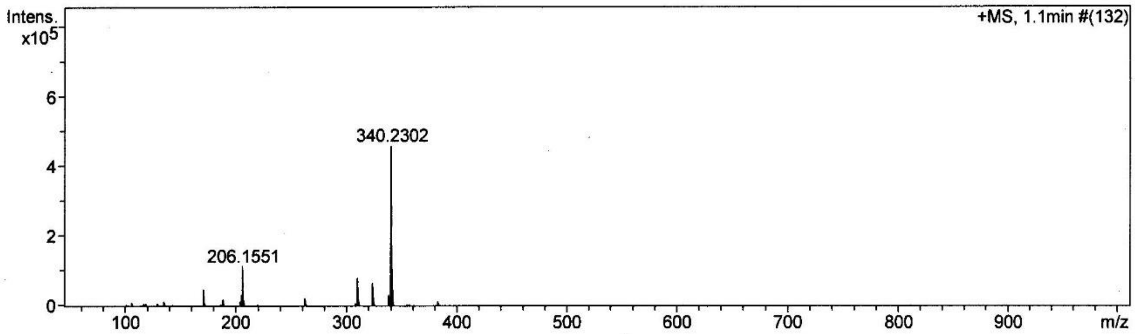

Meas. m/z	#	Formula	Score	m/z	err [mDa]	err [ppm]	mSigma	rdb	e⁻ Conf	N-Rule
340.2302	1	C 22 H 30 N O 2	100.00	340.2271	-3.0	-9.0	12.2	8.5	even	ok
	2	C 18 H 26 N 7	2.49	340.2244	-5.7	-16.9	24.8	9.5	even	ok

Generic Display Report

**Analysis Info**		Acquisition Date	10/7/2010 1:26:10 PM
Analysis Name	D:\Data\IPMC\PharmChemie\Routine\2019_10\WME_EM48Ab.d		
Method	directprobe_default.m	Operator	Meiners
Sample Name	EM48Ab	Instrument	micrOTOF-Q II
Comment	Meineke<br>APCI-Direkt<br>Kalibration mit APCI Calibrant Solution		

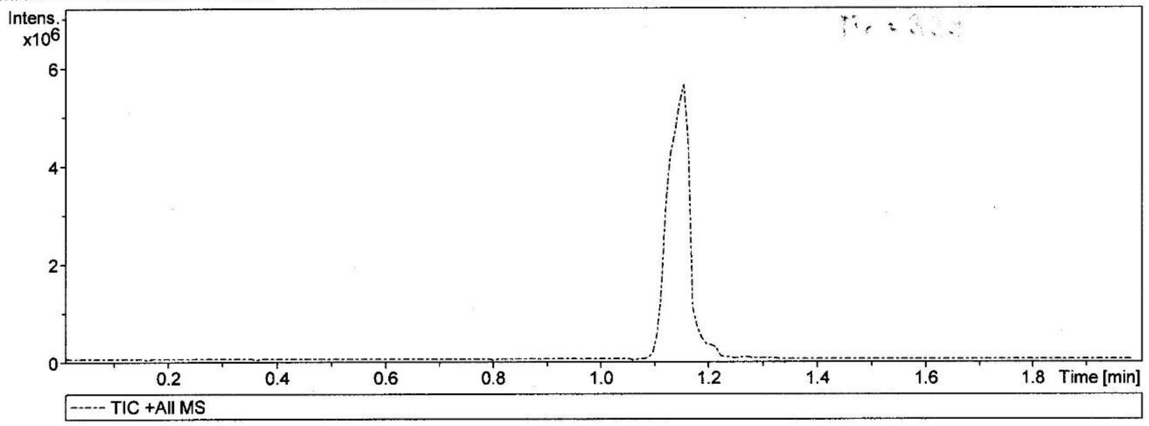

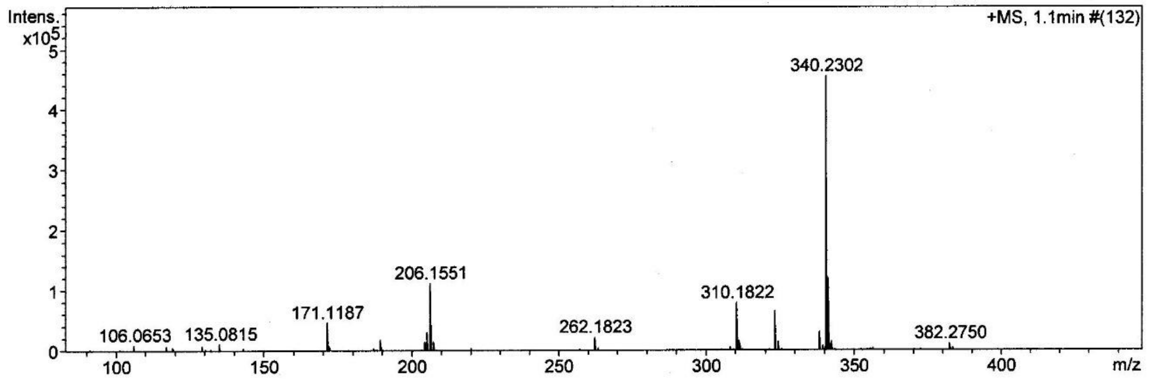

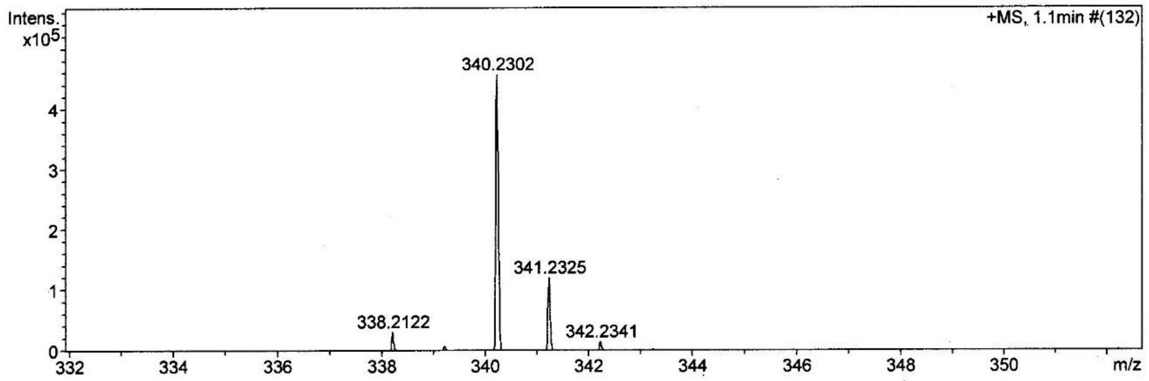

HPLC

Analyzed: 07.09.10 22:51

Reported: 08.09.10 14:45
Processed: 08.09.10 14:45

Data Path: D:\WIN32APP\HSM\Chromni\DATA\2081\
Application: Chromni
Sample Name: EM48Ab
Injection from this vial: 1 of 1

Series:2081
Vial Number: 7
Vial Type: UNK
Volume: 5,0 ul

Chrom Type: HPLC Channel : 1

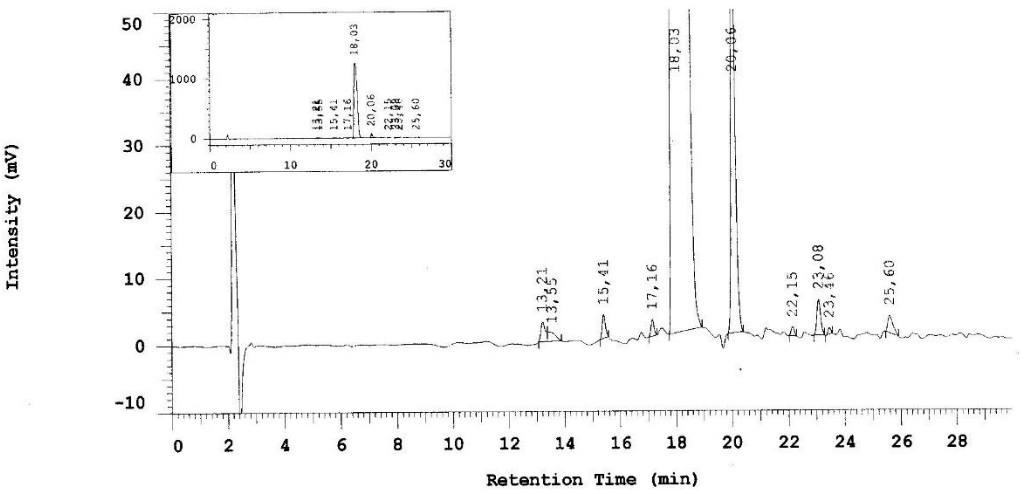

Acquisition Method: Chromni
Blank Subtr Sample Name: ACN
Column Type: 010
Developed by: Jens
Solvent A: Wasser + 0,05%TFA
Solvent B: ACN + 0,05%TFA

No.	RT	Area	Conc 1	BC
1	13,21	32090	0,096	MC
2	13,55	26391	0,079	MC
3	15,41	27613	0,082	MC
4	17,16	18315	0,055	MC
5	18,03	32630406	97,326	MC
6	20,06	694447	2,071	MC
7	22,15	10385	0,031	MC
8	23,08	48691	0,145	MC
9	23,46	6085	0,018	BB
10	25,60	32592	0,097	MC
		33527015	100,000	

Peak rejection level: 0

Spectroscopic and chromatographic data of compound **25a**

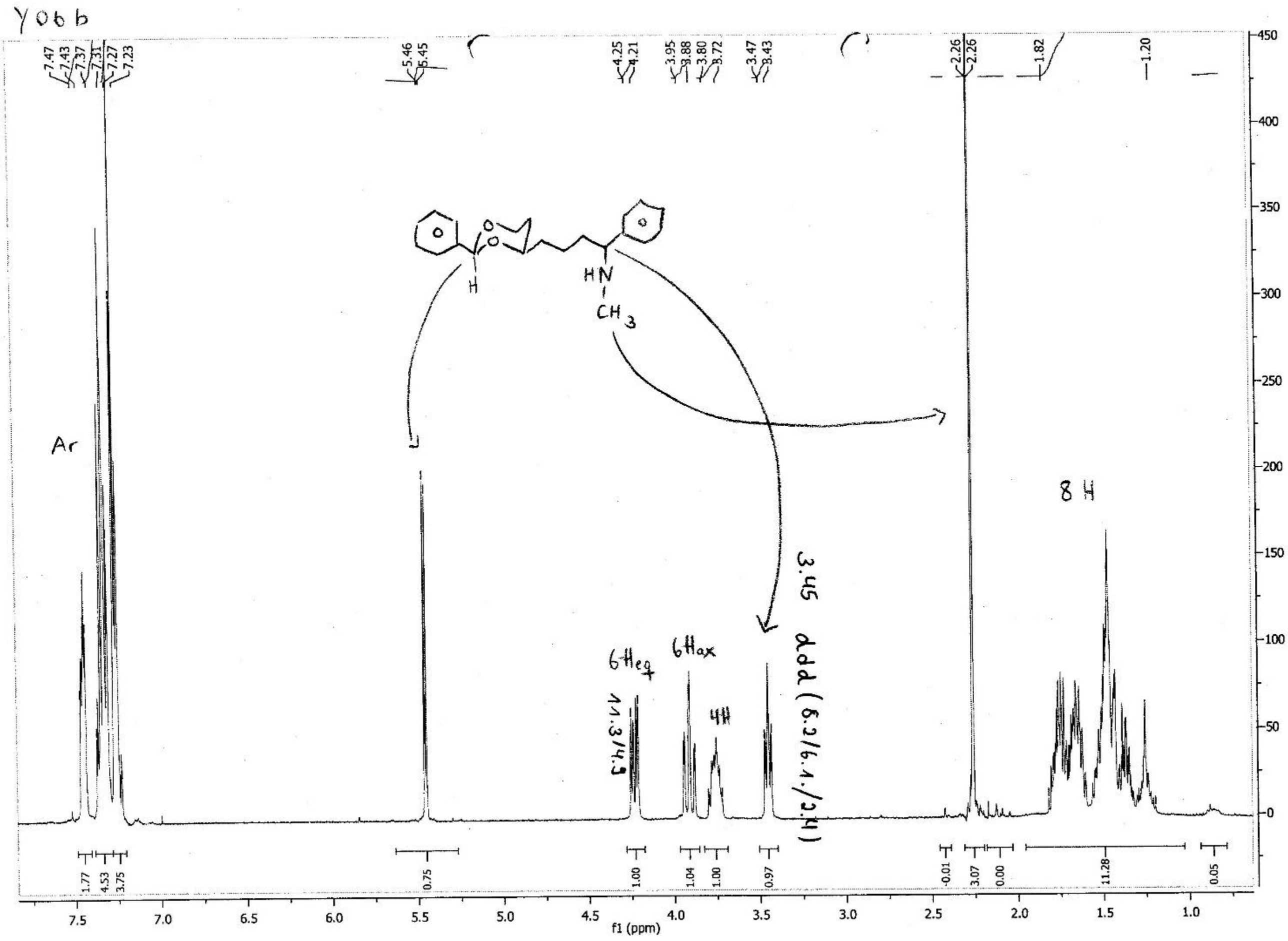

D:\Xcalibur\data\wme1020309 02.03.2009 13:28:51 Y06b

```
wme1020309
Type: Unknown ID:  Row: 1
Sample Name:          Y06b
Study:
Client:               Meineke
Laboratory:           Meiners
Company:
Phone:
Instrument Method:    D:\Xcalibur\methods\400ei.meth
Processing Method:
Vial:                 6
Injection Volume (µl): 2,00
Sample Weight:        0,00
Sample Volume (µl):   0,00
ISTD Amount:          0,00
Dil Factor:           0,00
```

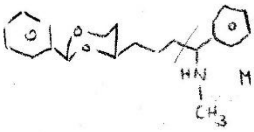

M_r = 325,46

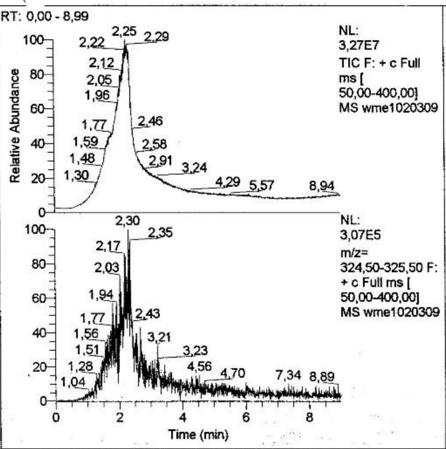

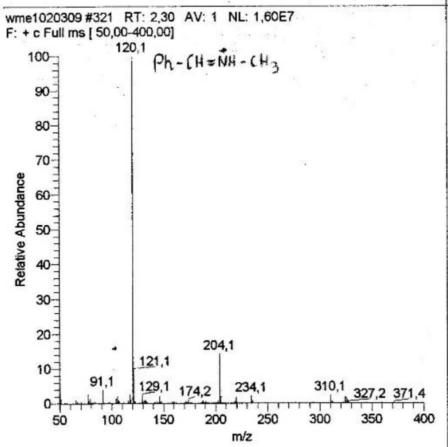

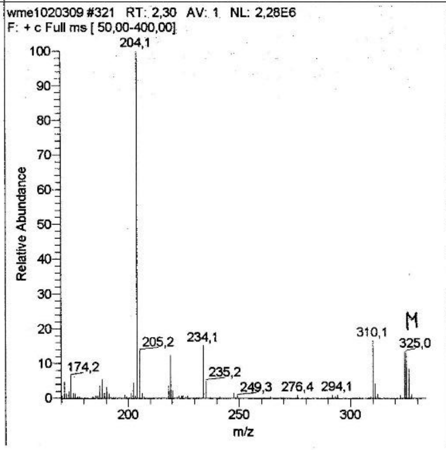

wme1020309#321 RT: 2,30
F: + c Full ms [50,00-400,00]

m/z	Intensity	Relative
50,9	213133,0	1,33
65,1	157348,0	0,98
67,2	82070,0	0,51
70,2	90102,0	0,56
77,2	415408,0	2,59
78,1	130123,0	0,81
79,2	230734,0	1,44
91,1	621890,0	3,88
104,1	202869,0	1,27
105,1	327521,0	2,04
106,2	139856,0	0,87
115,2	136746,0	0,85
117,2	412838,0	2,58
118,2	174224,0	1,09
120,1	16027392,0	100,00
121,1	1544230,0	9,63
122,0	106364,0	0,66
129,1	394581,0	2,46
131,2	114770,0	0,72
132,2	170222,0	1,06
146,2	321932,0	2,01
148,2	99160,0	0,62
159,1	89557,0	0,56
171,2	105963,0	0,66
174,2	147335,0	0,92
187,1	81265,0	0,51
188,1	123432,0	0,77
202,2	101750,0	0,63
204,1	2278709,0	14,22
205,2	312943,0	1,95
218,3	81877,0	0,51
219,2	280188,0	1,75
234,1	344572,0	2,15
235,2	113854,0	0,71
310,1	379155,0	2,37
311,2	98894,0	0,62
324,3	305083,0	1,90
325,0	307038,0	1,92
326,2	195418,0	1,22

nach 1. Säule

HPLC

Analyzed: 05.03.09 01:32

Reported: 05.03.09 11:15
Processed: 05.03.09 11:15

Data Path: D:\WIN32APP\HSM\Christoph\DATA\2082\
Application: Christoph

Series:2082

Sample Name: Y006
Injection from this vial: 1 of 1

Vial Number: 8
Vial Type: UNK
Volume: 5,0 ul

Chrom Type: HPLC Channel : 1

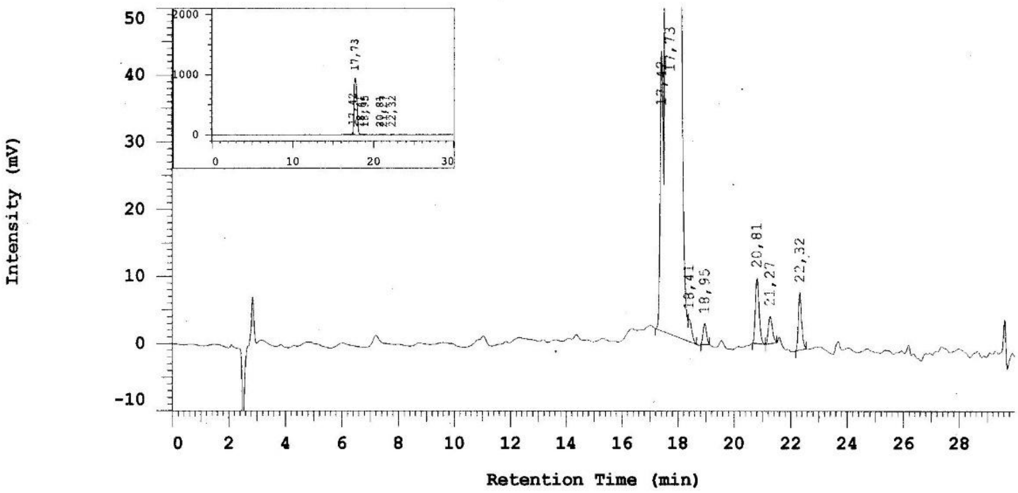

Acquisition Method: Chromni
Blank Subtr Sample Name: ACN
Column Type: 010
Developed by: Jens
Solvent A: Wasser + 0,05%TFA
Solvent B: ACN + 0,05%TFA

No.	RT	Area	Conc 1	BC
1	17,42	302478	1,353	MC
2	17,73	21793300	97,518	MC
3	18,41	28117	0,126	MC
4	18,95	25761	0,115	MC
5	20,81	88576	0,396	MC
6	21,27	39679	0,178	MC
7	22,32	69996	0,313	MC
		22347907	100,000	

Peak rejection level: 0

Spectroscopic and chromatographic data of compound **25b**

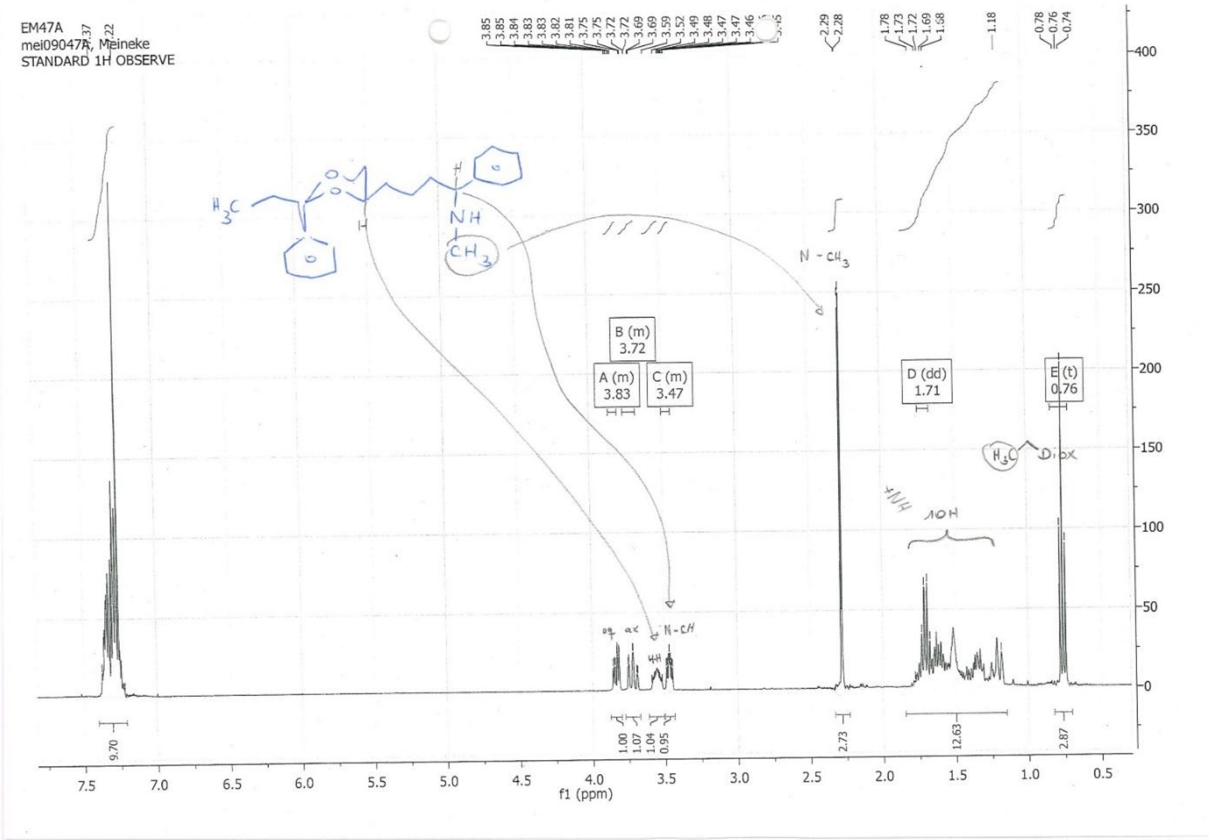

Generic Display Report

Analysis Info Acquisition Date 9/6/2010 11:08:11 AM

Analysis Name	D:\Data\IPMC\PharmChemie\Routine\2010_09\WME_EM47A.d		
Method	directprobe_default.m	Operator	Meiners
Sample Name	EM47A	Instrument	micrOTOF-Q II
Comment	Meineke<br>EM47A<br>APCI-Direkt<br>Kalibration mit Fettsaeureestern		

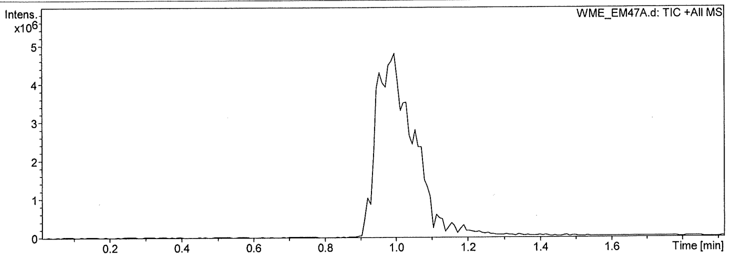

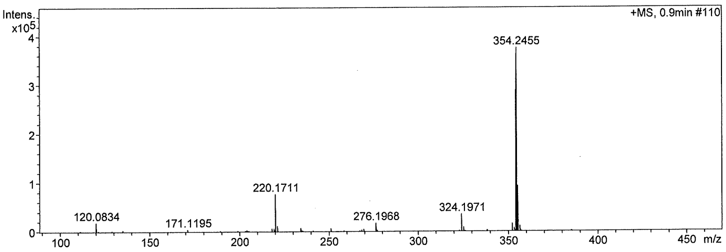

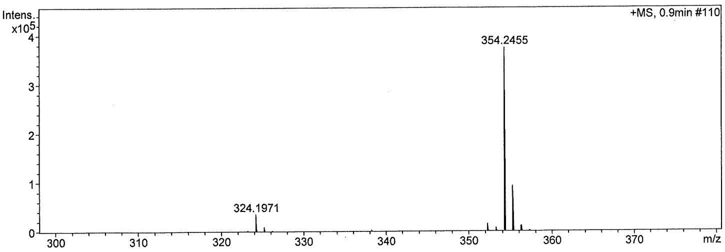

Mass Spectrum SmartFormula Report

Analysis Info

Analysis Name	D:\Data\IPMC\PharmChemie\Routine\2010_09\WME_EM47A.d	Acquisition Date	9/6/2010 11:08:11 AM
Method	directprobe_default.m	Operator	Meiners
Sample Name	EM47A	Instrument / Ser#	micrOTOF-Q II 10252
Comment	Meineke<br>EM47A<br>APCI-Direkt<br>Kalibration mit Fettsaeureestern		

Acquisition Parameter

Source Type	APCI	Ion Polarity	Positive	Set Nebulizer	1.0 Bar
Focus	Not active	Set Capillary	4000 V	Set Dry Heater	200 °C
Scan Begin	50 m/z	Set End Plate Offset	-500 V	Set Dry Gas	3.0 l/min
Scan End	1000 m/z	Set Collision Cell RF	130.0 Vpp	Set Divert Valve	Waste

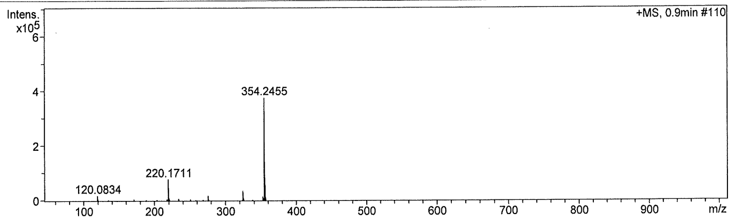

Meas. m/z	#	Formula	Score	m/z	err [mDa]	err [ppm]	mSigma	rdb	e⁻ Conf	N-Rule
354.2455	1	C 23 H 32 N O 2	100.00	354.2428	-2.7	-7.7	4.2	8.5	even	ok
	2	C 19 H 28 N 7	3.43	354.2401	-5.4	-15.3	11.3	9.5	even	ok
	3	C 17 H 32 N 5 O 3	9.84	354.2500	4.5	12.6	25.4	4.5	even	ok
	4	C 16 H 36 N O 7	31.69	354.2486	3.1	8.8	39.7	-0.5	even	ok

HPLC

Analyzed: 02.09.10 12:43

Reported: 02.09.10 18:03
Processed: 02.09.10 18:03

Data Path: D:\WIN32APP\HSM\Chromni\DATA\2067\
Application: Chromni
Sample Name: EM 47 A
Injection from this vial: 1 of 1

Series:2067
Vial Number: 24
Vial Type: UNK
Volume: 5,0 ul

Chrom Type: HPLC Channel : 1

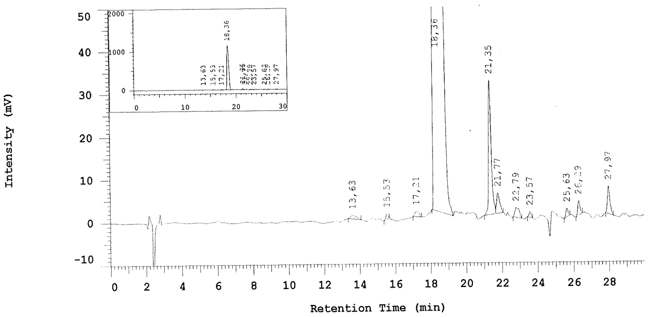

Acquisition Method: Chromni
Blank Subtr Sample Name: ACN
Column Type: 010

Developed by: Jens

Solvent A: Wasser + 0,05%TFA

Solvent B: ACN + 0,05%TFA

No.	RT	Area	Conc 1	BC
1	13,63	19108	0,069	MC
2	15,53	10566	0,038	BB
3	17,21	20267	0,073	MC
4	18,36	26994521	97,860	MC
5	21,35	325334	1,179	MC
6	21,77	58435	0,212	MC
7	22,79	39240	0,142	MC
8	23,57	10534	0,038	MC
9	25,63	15978	0,058	MC
10	26,29	28227	0,102	BB
11	27,97	62761	0,228	MC
		27584971	100,000	

Peak rejection level: 0

Spectroscopic and chromatographic data of compound **26a**

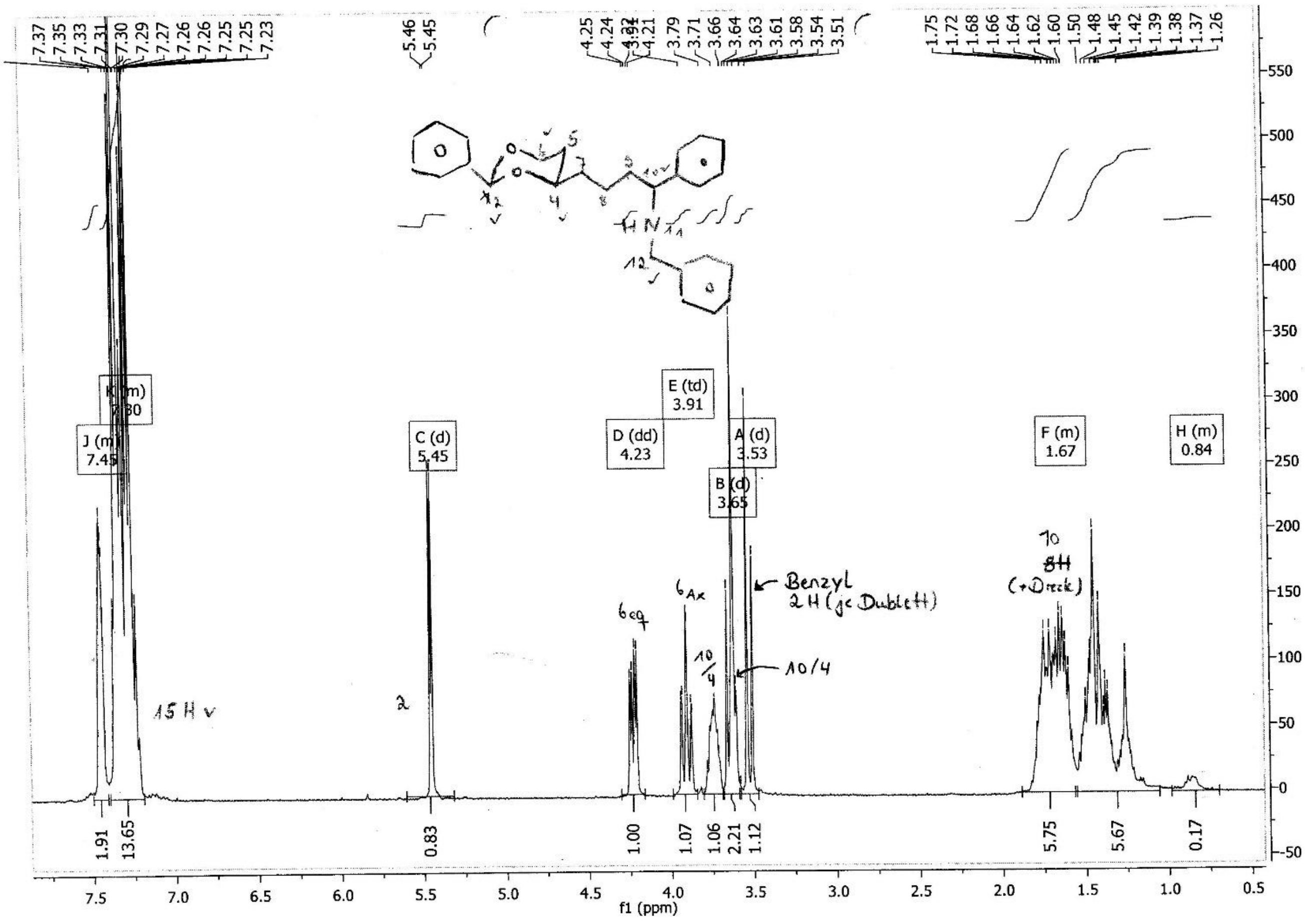

D:\Xcalibur\data\wme1181208 18.12.2008 14:42:33 EM14F

```
wme1181208
Type: Unknown ID:  Row: 1
Sample Name:            EM14F
Study:
Client:                 Meineke
Laboratory:             Meiners
Company:
Phone:
Instrument Method:      D:\Xcalibur\methods\500ei.meth
Processing Method:
Vial:                   1
Injection Volume (µl):  1,00
Sample Weight:          0,00
Sample Volume (µl):     0,00
ISTD Amount:            0,00
Dil Factor:             0,00
```

M = 401
(401,55)

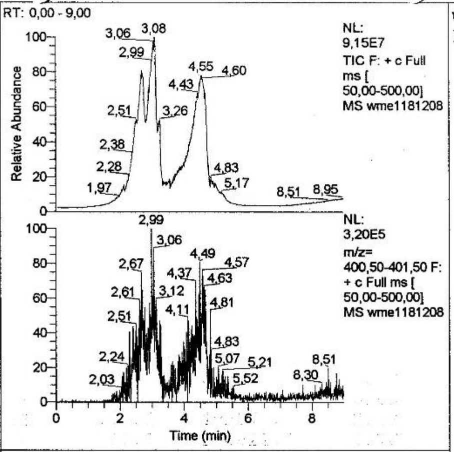

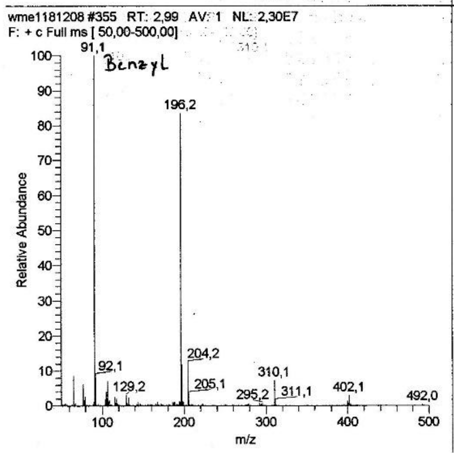

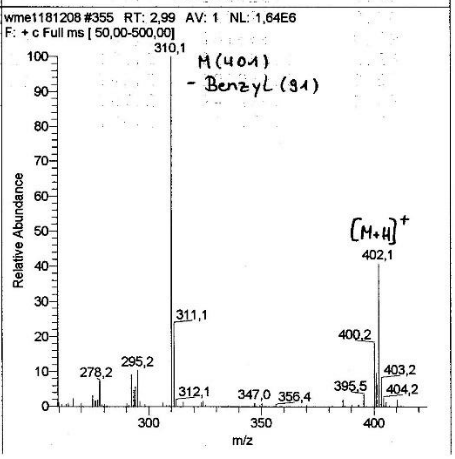

wme1181208#355 RT: 2,99
F: + c Full ms [50,00-500,00]

m/z	Intensity	Relative
50,9	300579,0	1,30
65,1	1961197,0	8,51
67,2	194934,0	0,85
77,1	1386356,0	6,01
78,2	361123,0	1,57
79,2	597241,0	2,59
90,5	266452,0	1,16
91,1	23048704,0	100,00
92,1	2075788,0	9,01
104,1	491795,0	2,13
105,2	921510,0	4,00
106,2	1641670,0	7,12
107,2	190984,0	0,83
108,1	348353,0	1,51
115,2	552965,0	2,40
116,1	159149,0	0,69
117,2	492077,0	2,13
118,1	174922,0	0,76
129,2	715120,0	3,10
130,1	181868,0	0,79
132,2	524593,0	2,28
143,2	220969,0	0,96
167,2	249418,0	1,08
186,1	217871,0	0,95
187,1	258671,0	1,12
188,3	210507,0	0,91
194,2	285317,0	1,24
195,2	249185,0	1,08
196,2	19255296,0	83,54
197,2	2716337,0	11,79
198,3	273345,0	1,19
204,2	2871886,0	12,46
205,1	866724,0	3,76
295,2	170431,0	0,74
310,1	1644757,0	7,14
311,1	389473,0	1,69
400,2	298760,0	1,30
401,2	158498,0	0,69
402,1	671897,0	2,92

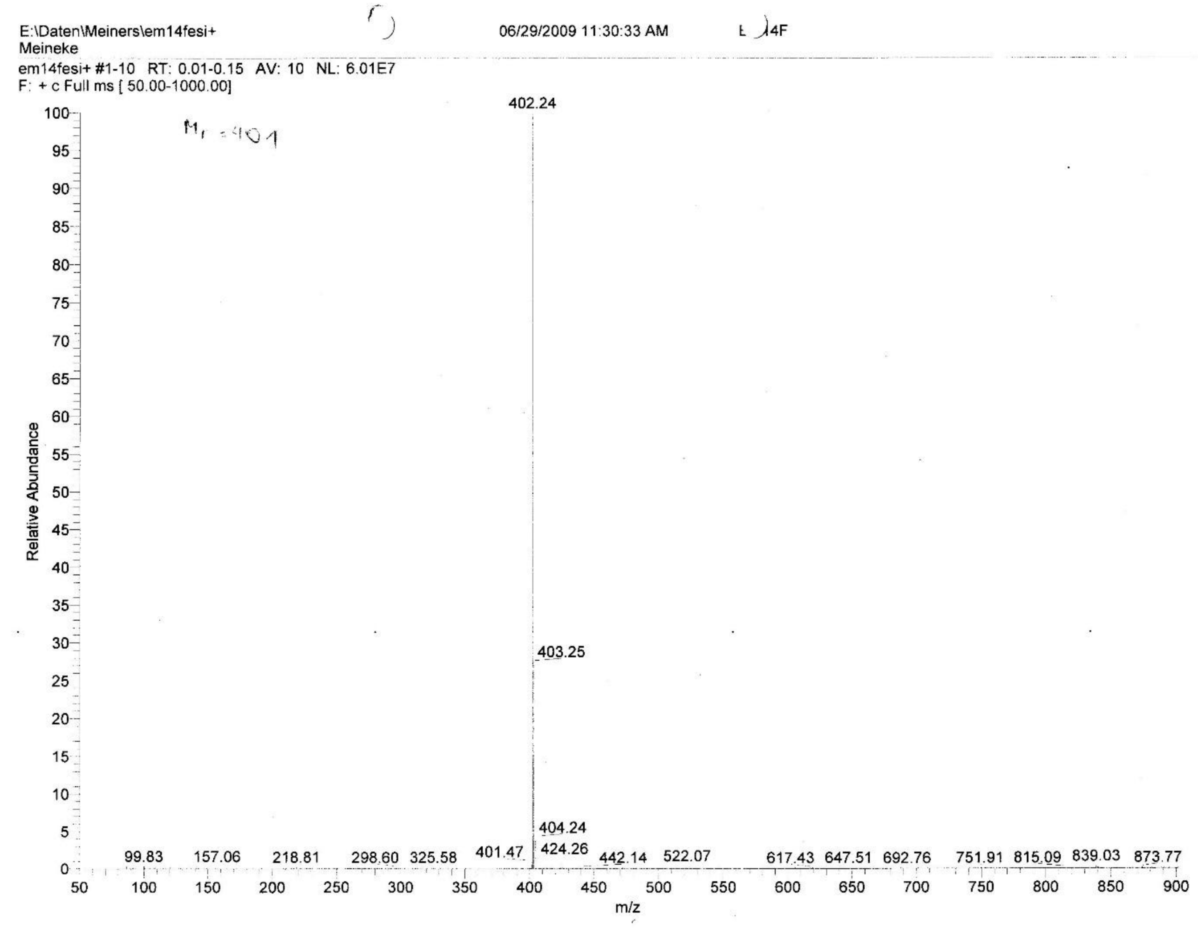
E:\Daten\Meiners\em14fesi+
Meineke
06/29/2009 11:30:33 AM
em14fesi+ #1-10 RT: 0.01-0.15 AV: 10 NL: 6.01E7
F: + c Full ms [50.00-1000.00]
Relative Abundance
m/z
402.24
403.25
404.24
424.26
401.47
99.83
157.06
218.81
298.60
325.58
442.14
522.07
617.43
647.51
692.76
751.91
815.09
839.03
873.77

HPLC

Analyzed: 21.01.09 22:30

Reported: 22.01.09 09:49
Processed: 22.01.09 09:48

Data Path: D:\WIN32APP\HSM\Christoph\DATA\1951\
Application: Christoph

Series:1951

Sample Name: EM14H

Vial Number: 5

Injection from this vial: 1 of 1

Vial Type: UNK
Volume: 5,0 ul

Chrom Type: HPLC Channel : 1

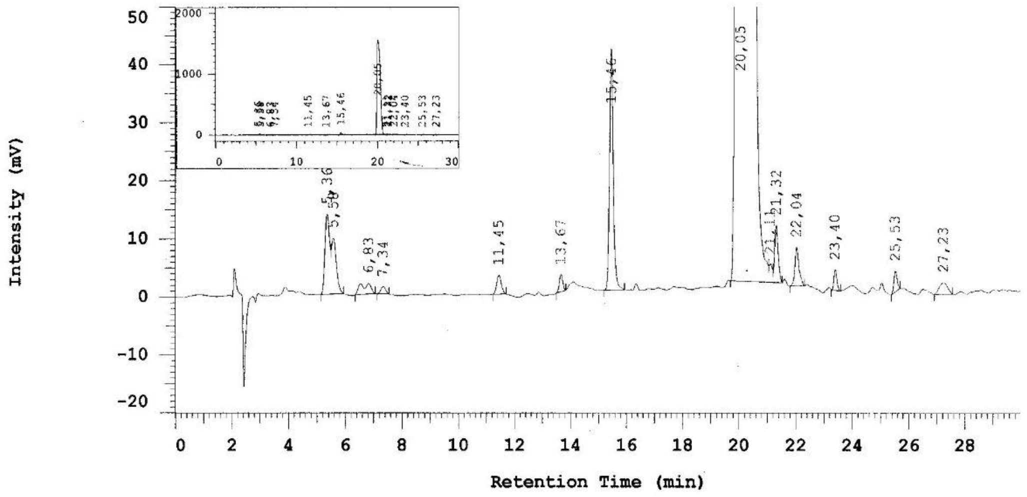

Acquisition Method: Chromni
Blank Subtr Sample Name: ACN
Column Type: 010

Developed by: Jens

Solvent A: Wasser + 0,05%TFA

Solvent B: ACN + 0,05%TFA

No.	RT	Area	Conc 1	BC
1	5,36	155348	0,359	MC
2	5,58	115633	0,267	MC
3	6,83	46917	0,109	MC
4	7,34	13632	0,032	MC
5	11,45	36251	0,084	MC
6	13,67	24570	0,057	BB
7	15,46	375172	0,868	MC
8	20,05	42196665	97,603	MC
9	21,11	24594	0,057	MC
10	21,32	83254	0,193	MC
11	22,04	65116	0,151	MC
12	23,40	26392	0,061	MC
13	25,53	27369	0,063	BB
14	27,23	41822	0,097	MC
		43232735	100,000	

Peak rejection level: 0

Spectroscopic and chromatographic data of compound **26b**

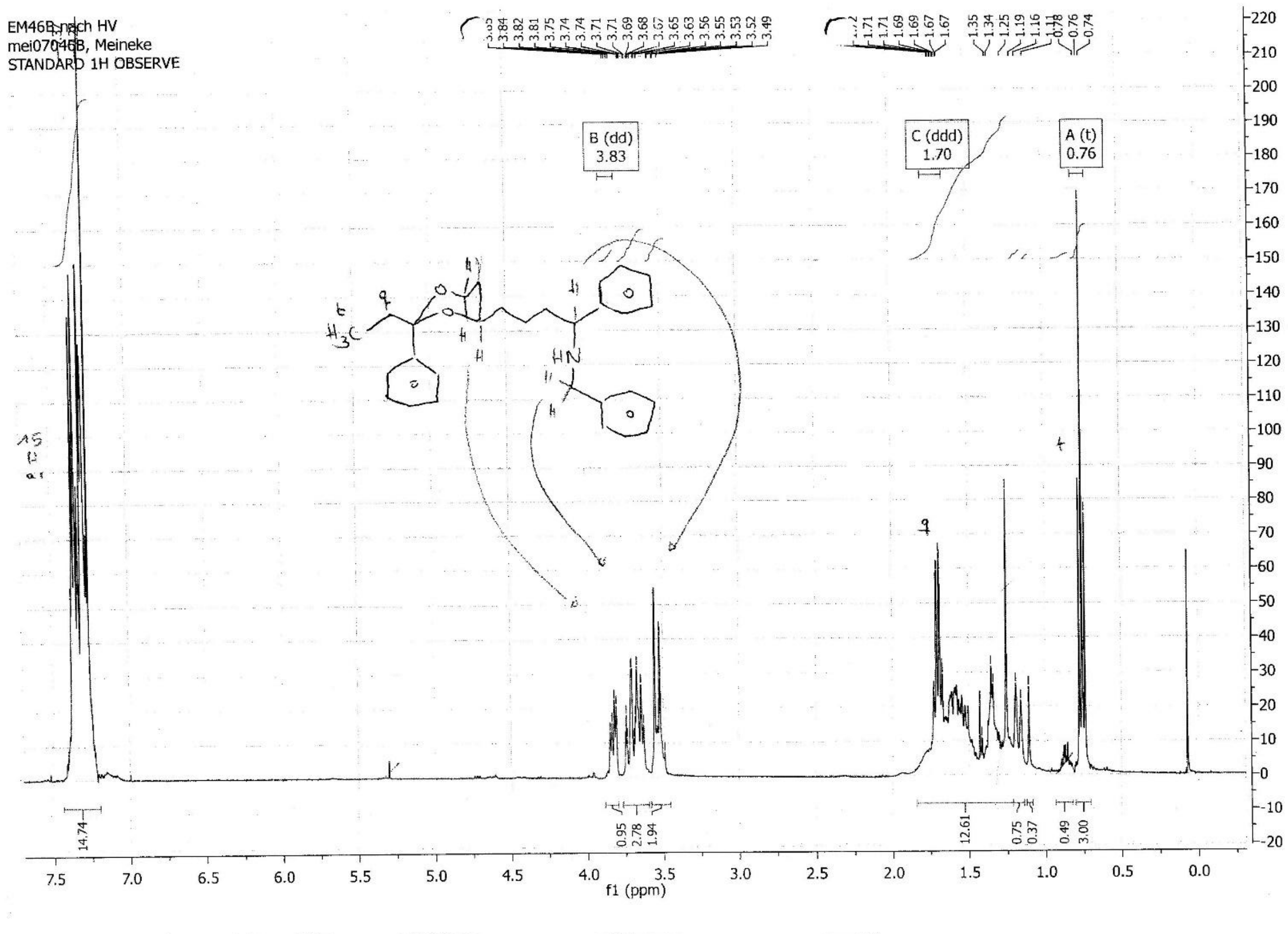

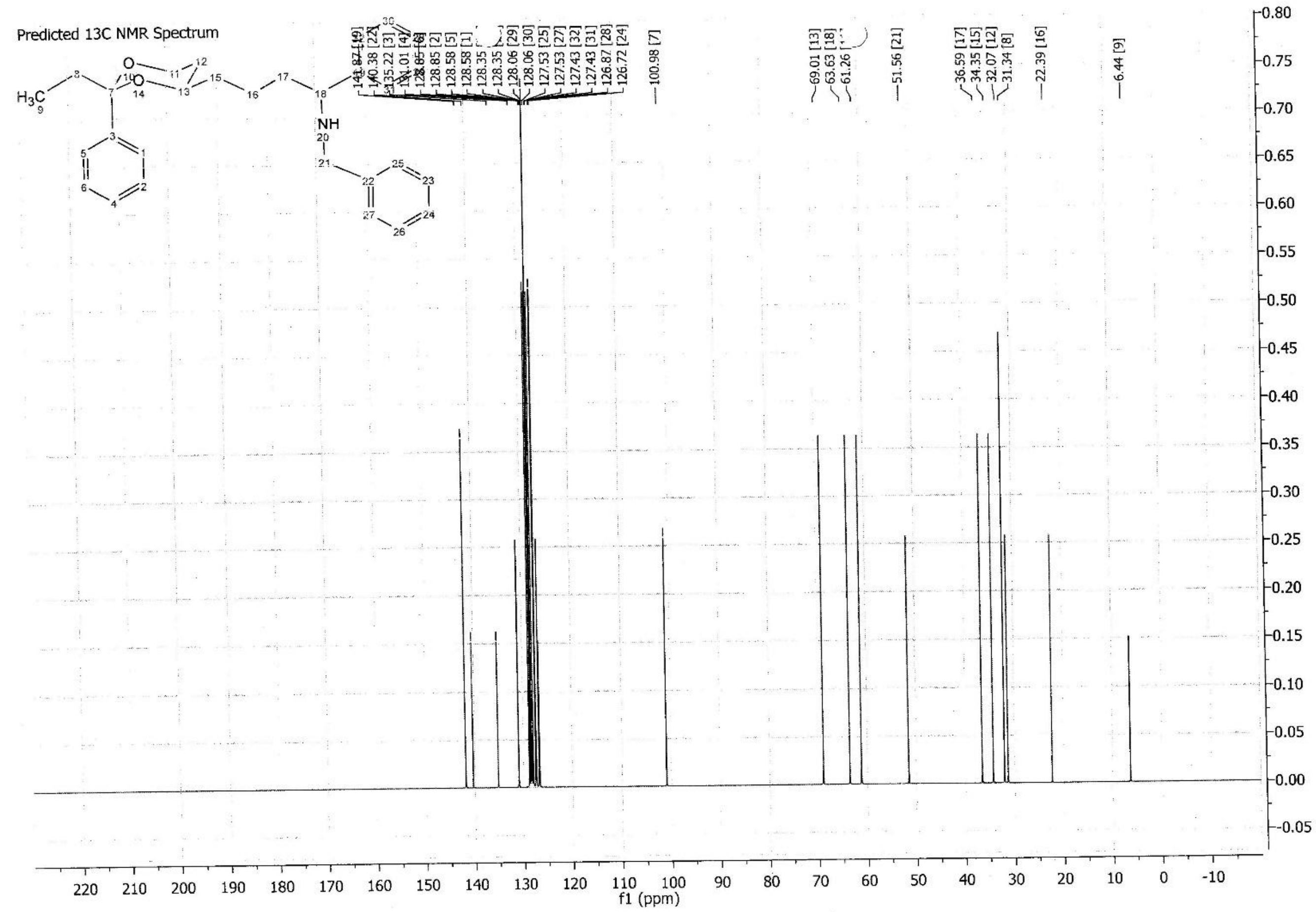
Predicted 13C NMR Spectrum
141.87 [19]
140.38 [22]
135.22 [3]
131.01 [4]
128.85 [2]
128.58 [5]
128.58 [1]
128.35
128.35
128.06 [29]
128.06 [30]
127.53 [25]
127.53 [27]
127.43 [32]
127.43 [31]
126.87 [28]
126.72 [24]
100.98 [7]
69.01 [13]
63.63 [18]
61.26
51.56 [21]
36.59 [17]
34.35 [15]
32.07 [12]
31.34 [8]
22.39 [16]
6.44 [9]
f1 (ppm)

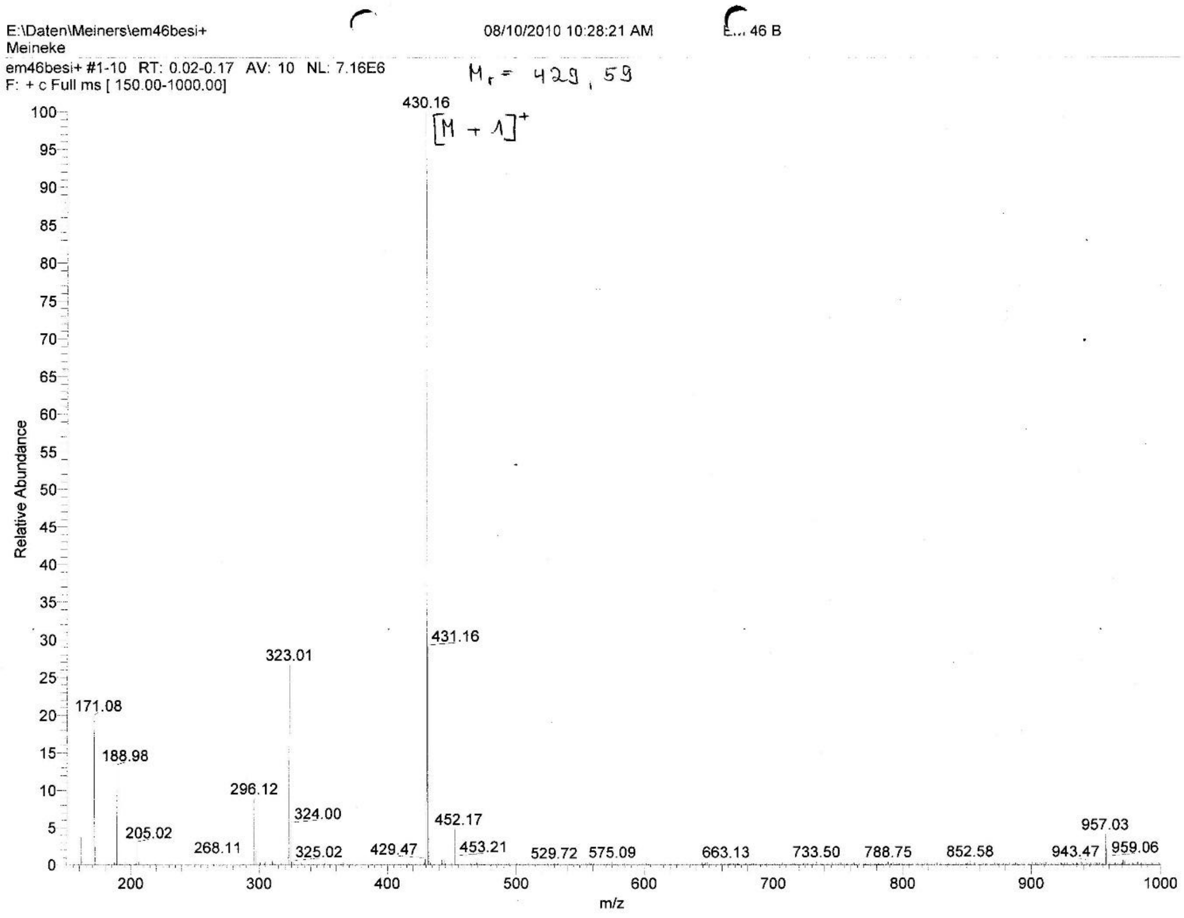
E:\Daten\Meiners\em46besi+
Meineke
08/10/2010 10:28:21 AM
E... 46 B
em46besi+ #1-10 RT: 0.02-0.17 AV: 10 NL: 7.16E6
F: + c Full ms [150.00-1000.00]
M_r = 429,59
[M + 1]^+
430.16
431.16
323.01
171.08
188.98
296.12
324.00
205.02
268.11
325.02
429.47
452.17
453.21
529.72
575.09
663.13
733.50
788.75
852.58
943.47
957.03
959.06
Relative Abundance
m/z

HPLC

Analyzed: 22.07.10 13:59

Reported: 26.07.10 08:35
Processed: 26.07.10 08:35

Data Path: D:\WIN32APP\HSM\Chromni\DATA\1834\
Application: Chromni
Sample Name: EM46B
Injection from this vial: 1 of 1

Series:1834
Vial Number: 25
Vial Type: UNK
Volume: 5,0 ul

Chrom Type: HPLC Channel : 1

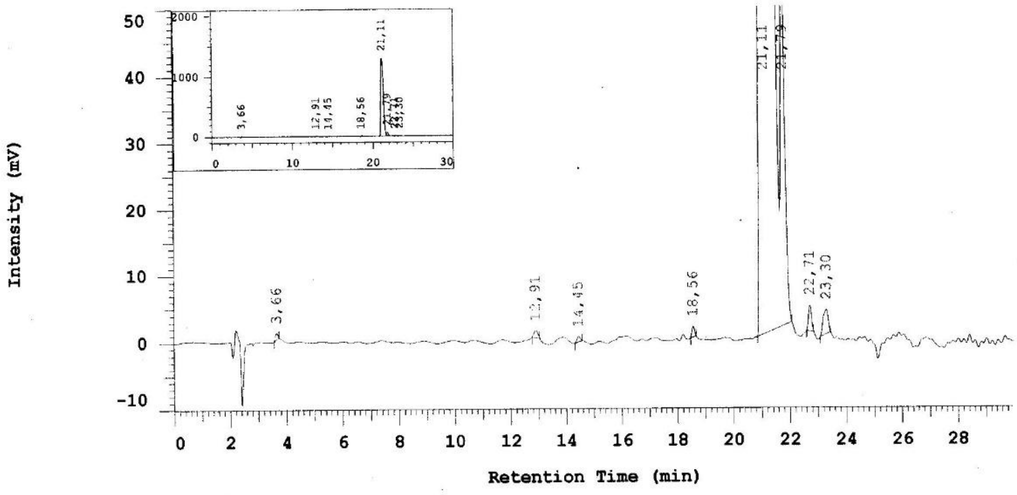

Acquisition Method: Chromni
Blank Subtr Sample Name: ACN
Column Type: 010
Developed by: Jens
Solvent A: Wasser + 0,05%TFA
Solvent B: ACN + 0,05%TFA

No.	RT	Area	Conc 1	BC
1	3,66	5030	0,018	MC
2	12,91	9844	0,034	BB
3	14,45	6147	0,021	BB
4	18,56	9731	0,034	MC
5	21,11	27867648	97,405	MC
6	21,79	640496	2,239	MC
7	22,71	27308	0,095	MC
8	23,30	43911	0,153	MC
		28610115	100,000	

Peak rejection level: 0

Spectroscopic and chromatographic data of compound **27a**

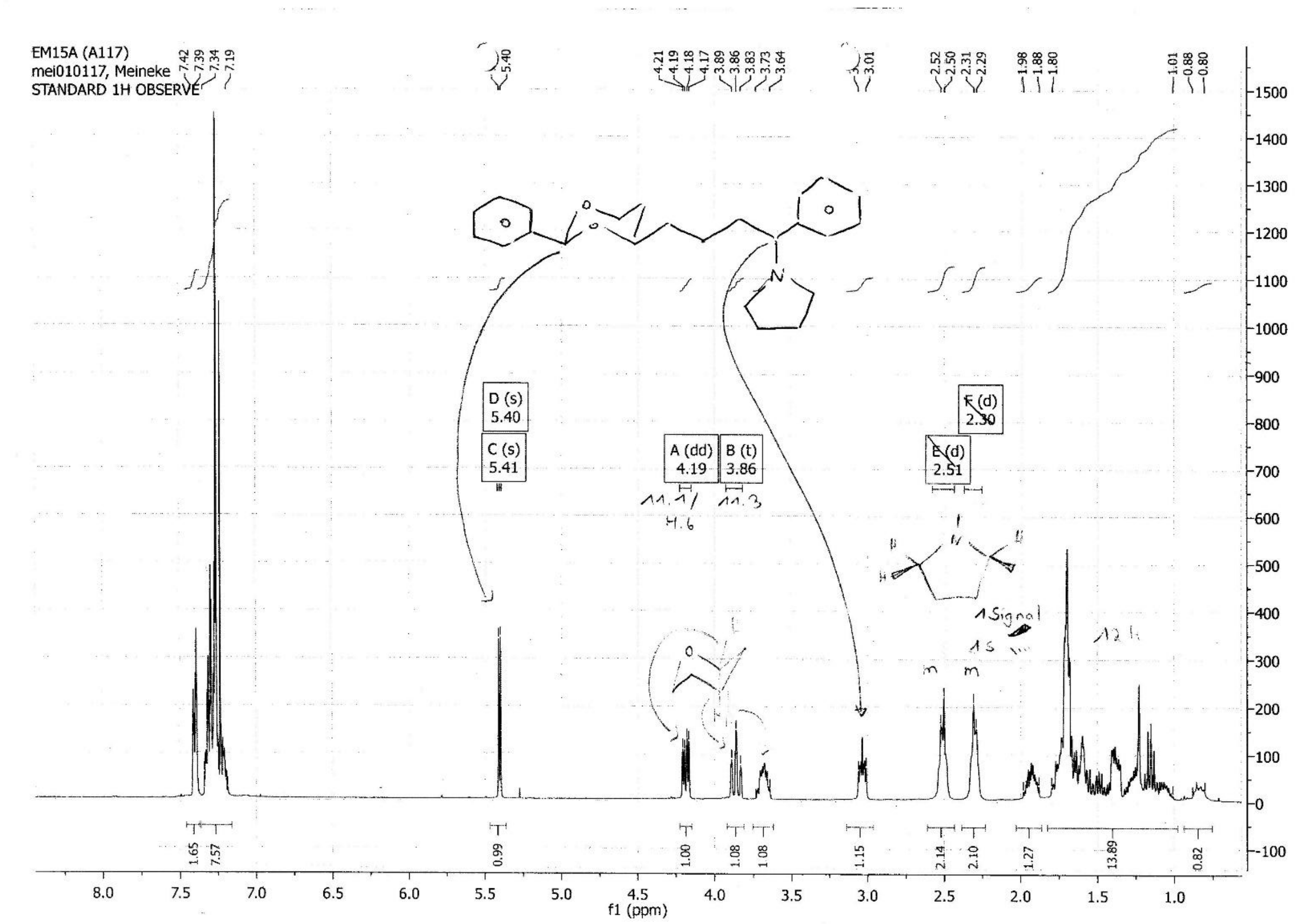

D:\Xcalibur\data\wme1280109 28.01.2009 11:52:55 A117

wme1280109
Type: Unknown ID: Row: 1
Sample Name: A117
Study:
Client: Meineke
Laboratory: Meiners
Company:
Phone:
Instrument Method: D:\Xcalibur\methods\500ei.meth
Processing Method:
Vial: 1
Injection Volume (µl): 1,00
Sample Weight: 0,00
Sample Volume (µl): 0,00
ISTD Amount: 0,00
Dil Factor: 0,00

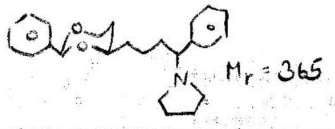

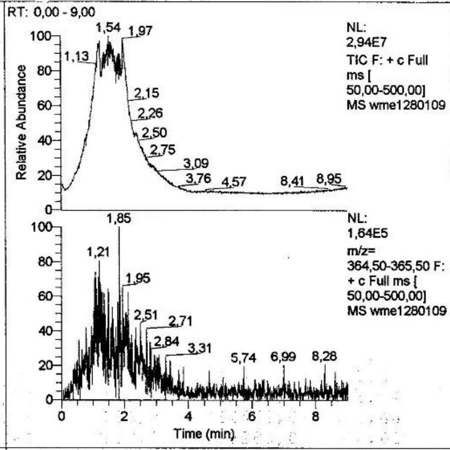

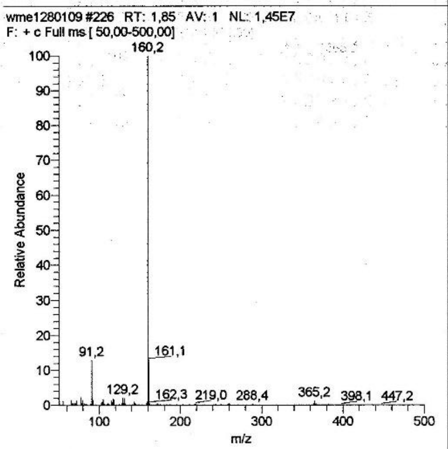

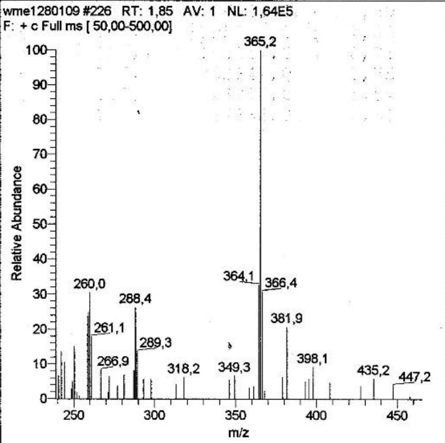

wme1280109#226 RT: 1,85
F: + c Full ms [50,00-500,00]

m/z	Intensity	Relative
50,9	152393,0	1,05
55,1	170226,0	1,17
65,1	198800,0	1,37
67,2	87847,0	0,61
70,2	111239,0	0,77
72,2	185172,0	1,28
77,1	317145,0	2,19
78,2	93345,0	0,64
79,2	206824,0	1,43
81,2	95634,0	0,66
89,1	60842,0	0,42
91,2	1861424,0	12,85
92,2	201648,0	1,39
103,2	109211,0	0,75
104,2	144417,0	1,00
105,2	252061,0	1,74
106,2	64452,0	0,44
110,2	88280,0	0,61
111,2	56523,0	0,39
115,1	195228,0	1,35
116,1	103017,0	0,71
117,2	252842,0	1,75
118,2	223287,0	1,54
128,2	87835,0	0,61
129,2	298395,0	2,06
130,2	81435,0	0,56
131,2	294834,0	2,04
132,2	75458,0	0,52
143,2	134001,0	0,92
144,2	81117,0	0,56
145,2	58820,0	0,41
158,2	68949,0	0,48
159,4	143846,0	0,99
160,2	14487552,0	100,00
161,1	1896902,0	13,09
162,3	85016,0	0,59
171,2	68999,0	0,48
172,1	57331,0	0,40
365,2	164239,0	1,13

HPLC

Analyzed: 05.02.09 03:45

Reported: 05.02.09 09:19
Processed: 05.02.09 09:19

Data Path: D:\WIN32APP\HSM\Christoph\DATA\2008\
Application: Christoph

Series:2008

Sample Name: EM177

Vial Number: 15
Injection from this vial: 1 of 1

Vial Type: UNK
Volume: 5,0 ul

Chrom Type: HPLC Channel : 1

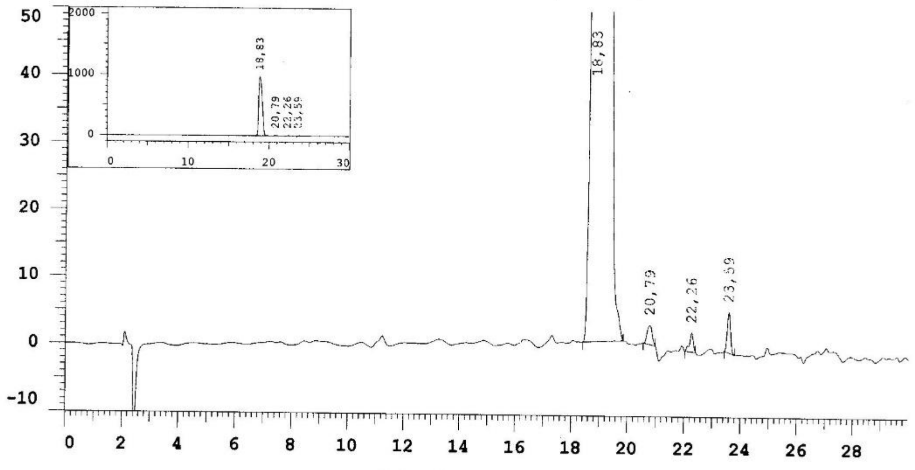

Acquisition Method: Chromni
Blank Subtr Sample Name: ACN
Column Type: 010

Developed by: Jens

Solvent A: Wasser + 0,05%TFA

Solvent B: ACN + 0,05%TFA

No.	RT	Area	Conc 1	BC
1	18,83	27378483	99,609	MC
2	20,79	34416	0,125	MC
3	22,26	25496	0,093	MC
4	23,59	47572	0,173	BB
		27485967	100,000	

Peak rejection level: 0

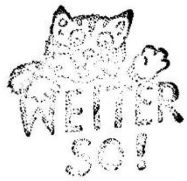

Spectroscopic and chromatographic data of compound **27b**

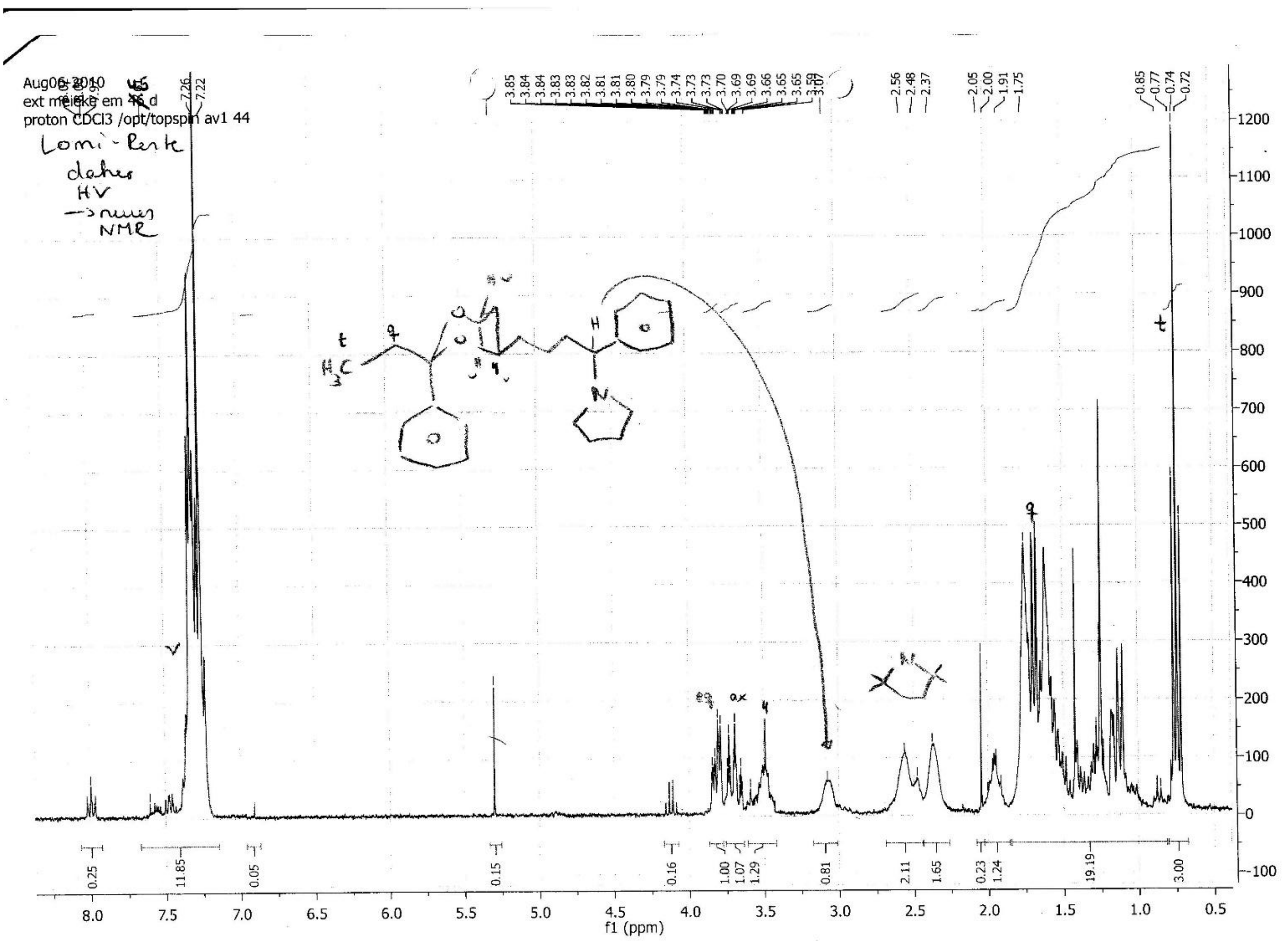

MicroTof (Bruker Daltonics) OC-Münster Abt. Massenspektrometrie

Analysis Info

Analysis Name	D:\Data\aktuell\Wuensch EM 46 D_Tray1-12_01_119423.d	Acquisition Date	8/6/2010 6:04:36 PM
Sample Name	Wuensch EM ~~46 D~~ 45 D	Method	oa_ms_pos_lm_ot.m
Comment	MeOH pur		

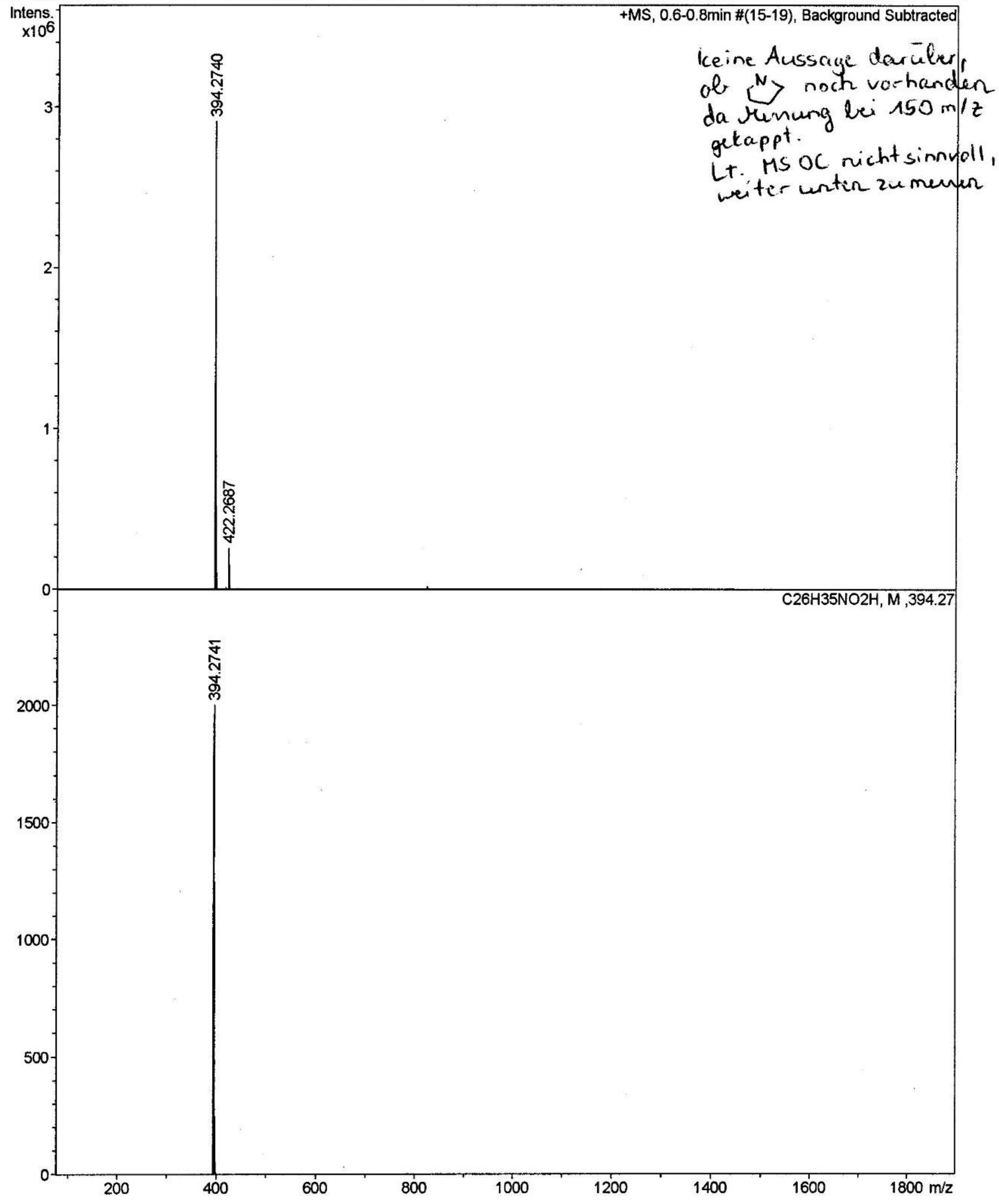

HPLC

Analyzed: 05.08.10 08:23

Reported: 05.08.10 14:45
Processed: 05.08.10 14:45

Data Path: D:\WIN32APP\HSM\Chromni\DATA\1898\
Application: Chromni

Sample Name: EM45D

Injection from this vial: 1 of 1

Series:1898
Vial Number: 19
Vial Type: UNK
Volume: 5,0 ul

Chrom Type: HPLC Channel : 1

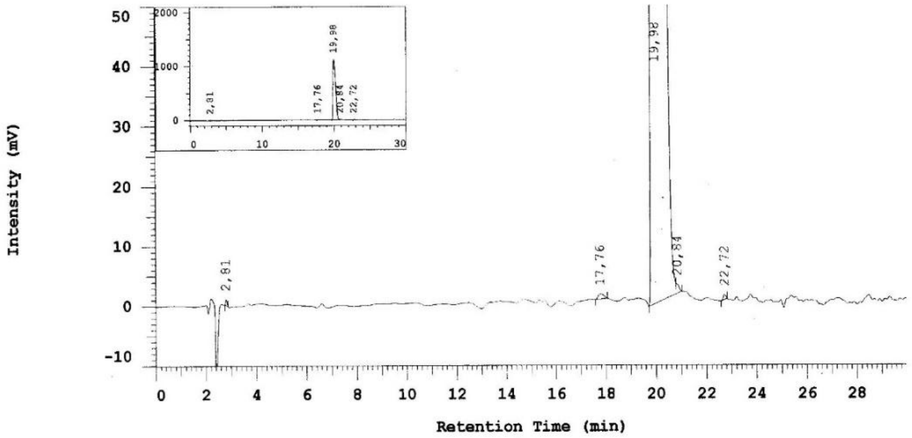

Acquisition Method: Chromni
Blank Subtr Sample Name: ACN
Column Type: 010
Developed by: Jens
Solvent A: Wasser + 0,05%TFA
Solvent B: ACN + 0,05%TFA

No.	RT	Area	Conc 1	BC
1	2,81	3853	0,014	MC
2	17,76	13081	0,048	MC
3	19,98	27026960	99,871	MC
4	20,84	12675	0,047	MC
5	22,72	5412	0,020	BB
		27061981	100,000	

Peak rejection level: 0
